# Organic Solvents as Risk Factor for Autoimmune Diseases: A Systematic Review and Meta-Analysis

**DOI:** 10.1371/journal.pone.0051506

**Published:** 2012-12-19

**Authors:** Carolina Barragán-Martínez, Cesar A. Speck-Hernández, Gladis Montoya-Ortiz, Rubén D. Mantilla, Juan-Manuel Anaya, Adriana Rojas-Villarraga

**Affiliations:** 1 Center for Autoimmune Diseases Research (CREA), School of Medicine and Health Sciences, Universidad del Rosario, Bogota, Colombia; Wadsworth Center, United States of America

## Abstract

**Background:**

Genetic and epigenetic factors interacting with the environment over time are the main causes of complex diseases such as autoimmune diseases (ADs). Among the environmental factors are organic solvents (OSs), which are chemical compounds used routinely in commercial industries. Since controversy exists over whether ADs are caused by OSs, a systematic review and meta-analysis were performed to assess the association between OSs and ADs.

**Methods and Findings:**

The systematic search was done in the PubMed, SCOPUS, SciELO and LILACS databases up to February 2012. Any type of study that used accepted classification criteria for ADs and had information about exposure to OSs was selected. Out of a total of 103 articles retrieved, 33 were finally included in the meta-analysis. The final odds ratios (ORs) and 95% confidence intervals (CIs) were obtained by the random effect model. A sensitivity analysis confirmed results were not sensitive to restrictions on the data included. Publication bias was trivial. Exposure to OSs was associated to systemic sclerosis, primary systemic vasculitis and multiple sclerosis individually and also to all the ADs evaluated and taken together as a single trait (OR: 1.54; 95% CI: 1.25–1.92; p-value<0.001).

**Conclusion:**

Exposure to OSs is a risk factor for developing ADs. As a corollary, individuals with non-modifiable risk factors (i.e., familial autoimmunity or carrying genetic factors) should avoid any exposure to OSs in order to avoid increasing their risk of ADs.

## Introduction

Autoimmune diseases (ADs) are initiated by the loss of immune tolerance and mediated through T or B cell activation leading to tissue damage. ADs share clinical signs and symptoms, physiopathological mechanisms, and genetic factors [1]. They are complex diseases caused by the interaction between genetic, epigenetic, and environmental factors over time [2], [3].

Despite the difficulties in defining environmental risk factors that lead to immunopathology, the number of candidates proposed for specific ADs is continuously growing as new evidence is reported for infectious agents, chemicals, physical factors, adjuvants, and hormones [4]–[15]. A significant body of research has pointed out that, for autoimmunity to occur, the genetic background warrants to be combined with environmental injuries and novel associations has been described as the case of the air pollution [5], [16]. However these environmental factors often explain only a small number of cases, and, on their own, they are not sufficient to cause the disease [5].

Solvents are liquids that dissolve a solid, liquid or gas. They can be broadly classified into two categories: organic and inorganic. Organic solvents (OSs) are compounds whose molecules contain carbon. They may be broken down further into aliphatic-chain compounds, such as n-hexane, and aromatic compounds with a 6-carbon ring, such as benzene or xylene. OSs arose in the latter half of the 19th century from the coal-tar industry. Common uses for OSs are: dry cleaning (e.g., tetrachloroethylene), paint thinner (e.g., toluene, turpentine), nail polish removers and glue solvents (acetone, methyl acetate, ethyl acetate), spot removers (e.g., hexane, petrol ether), detergents (citrus turpenes), perfumes (ethanol), nail polish and chemical synthesis [17]. In contrast, the use of inorganic solvents (other than water) is typically limited to research in chemistry and some technological processes.

The applications of OSs became more diversified in both developed and developing countries. Research in this area began in 1957 when the first patients developing a scleroderma-like syndrome after exposure to vinyl chloride, epoxy resins, trichloroethylene (TCE), perchloroethylene and other mixed solvents were reported [18], [19]. Nevertheless, few published studies have analyzed the wide spectrum of ADs in subjects exposed to OSs. Therefore, we aimed to analyze the evidence of an association between the exposure to OSs and the development of AD through a systematic literature review and a meta-analysis. In addition, a comprehensive review concerning the mechanisms by which OSs exposure induces immunological alterations is presented.

## Methods

### Literature Search

The search was done using the following databases: PubMed, SCOPUS, SciELO and LILACS and took into account articles published up to February 2012. We followed the PRISMA guidelines for meta-analysis of observational studies [20] in our data extraction, analysis, and reporting (Text S1).

The most relevant terms regarding OSs exposure were suggested by an expert chemical engineer specialist in industrial hygiene. The following Medical Subject Heading (MeSH) terms were used: “systemic vasculitis,” “vasculitis,” “rheumatoid arthritis,” “lupus,” “multiple sclerosis,” “scleroderma,” “systemic sclerosis,” “antiphospholipid syndrome,” “Sjögren's syndrome,” “dermatomyositis,” “polymyositis,” “myasthenia gravis,” “Churg-Strauss syndrome,” “giant cell arteritis,” “microscopic polyangiitis,” “cryoglobulinemia,” “polyarteritis nodosa,” “Wegener granulomatosis,” “inflammatory bowel diseases,” “anemia, pernicious,” “thyroiditis, autoimmune,” “celiac disease,” “juvenile rheumatoid arthritis,” “vitiligo,” “primary biliary cirrhosis,” “biliary cirrhosis,” “primary sclerosing cholangitis,” “autoimmune hepatitis,” “transverse myelitis,” “relapsing polychondritis,” “Addison disease,” “glomerulonephritis,” “idiopathic thrombocytopenic purpura,” “psoriatic arthritis,” “spondylitis, ankylosing,” “sarcoidosis,” “Raynaud's disease,” “connective tissue disease,” and “autoimmune disease.” Each one of them was cross-referenced with the following MeSH terms: “solvent,” “tetrachloroethylene,” “trichloroethylene,” “trichloroethane,” “perchlorethylene,” “toluene,” “vinyl chloride,” “acetone,” “ethylacetate,” “turpentine,” “benzene,” “5-hydroxytryptophan,” “diethylpropion,” “fenfluramine,” and “hair dye.” Furthermore, we used ‘text words’ if there was no MeSH term such as in the cases of “hexane,” “white spirit,” “urea formaldehyde,” and “nail polish.”

In addition, each MeSH term was translated into DeCS (Health Sciences Descriptors), the tool that permits navigation between records and sources of information through controlled concepts and organized in Portuguese, Spanish and English, in order to search the SciElo and LILACS databases. No limits regarding language, period of publication, or publication type were taken into account. Those references from the articles that seemed to be relevant for our review were hand-searched. Authors of publications to which full text access was unavailable were contacted via e-mail.

In addition, a systematic search was done in the PubMed database up to February 2012 looking for the molecular mechanisms by which OSs may alter immune responses and induce the developing of ADs. The search was restricted to: (1) studies in humans and mice, (2) restricted by title and (3) English language, (4) articles published in the last 20 years, (5) studies in ADs (6) studies including the term autoimmunity, (7) studies including the term “immune system”. All of the search strategies included MeSH terms: “Tetrachloroethylene”, “Trichloroethylene”, “Trichloroethanes”, “Perchlorethylene”, “Toluene”, “Vinyl Chloride”, “Acetone”, “Ethylacetate”, “Turpentine”, “ Benzene ”, “5-Hydroxytryptophan”, “Diethylpropion”, “Fenfluramine”, “Hair Dyes”, “Hexane”, “Immune System”, and “autoimmunity”. Furthermore, we used key words if there was no MeSH term such as in the cases of “white spirit”, “urea formaldehyde”, “nail polish and “autoimmune diseases”. The exclusion criteria were the following: 1) Articles related to immune alterations due to allergic responses in solvent exposure, 2) articles related to cytotoxic and genotoxic effects of solvent in cancer progression, 3) articles in a language other than English, 4) reviews, 5) comments and case reports that did not report any biological implication related to solvent exposure.

### Study Selection, Data Extraction, and Quality Assessment

Inclusion criteria for the systematic review were the following: any types of study that used accepted classification criteria for ADs and had information about exposure to OSs explicitly listed as a category.

Articles were excluded from the analysis if they included the same data that were published in another study.

Abstracts and full text articles were reviewed in the search for eligible studies. Two reviewers did the search independently while applying the same selection criteria. The two resulting databases were compared and disagreements resolved by consensus. For articles in languages other than English or Spanish, translations of abstracts or full text articles were reviewed to determine eligibility.

Each eligible study was classified as: review, case report, case series, cohort, or case-control. Inclusion criteria for the meta-analysis were applied to publications that provided epidemiologic data on risk factors [relative risks (RR) and odds ratios (OR) with confidence intervals (CI)] or that provided information that let us calculate these data. For cohort studies, the requirements were the number of subjects exposed, the number unexposed, and the number of subjects who developed the disease in each of the two cases. For case-control studies, the requirements were the number of subjects with AD that were exposed and not exposed, and the number of controls that were exposed and not exposed. In those instances where the study had not reported the number of subjects in each group, either the RR or the OR with the CI, at least, must have been reported in order for them to be included in the meta-analysis calculations.

Studies were excluded from the meta-analysis if the groups were not clearly defined, e.g. case- controls studies with likely AD diagnosis in control subjects or exposed cohorts with low specificity for OS.

For each eligible study, the type of exposure and exposure assessment was analyzed regarding the source of information (census, database, interview, mailed questionnaires, etc.) and classified as follows: “qualitative” if it was stated by the subject or interviewer on questionnaires measured by the quality of exposure rather than its quantity, “quantitative” if it was related to a number or quantity, and “semi-quantitative” if it was expressed as a quantity susceptible of measurement but was not related to a number. Quantitative assessment was sub-classified in “indirect quantitative” if it was defined by an estimate from a register of specific jobs at risk or calculated using a job-exposure matrix formula, and “direct quantitative” if the OS was directly measured in the environment or as a biomarker in the subject. Furthermore it was extracted the information that described the condition of exposure (e.g occupation, living characteristics.)

The quality and strength of scientific evidence was evaluated supporting an etiologic relationship between ADs and the proposed risk factor. In this investigation, a quantitative scoring system based on the Bradford Hill criteria was used [21]. The quantitative Bradford Hill score (qBHs) is divided into categorical ratings of the overall strength of causal association as follows: 0 to 6 points was considered poor or no causal association; 7 to 14 points was considered moderate or inconclusive causal association, and 15 to 21 points was considered a strong causal association. No study was excluded from the review based on this assessment.

### Meta-analysis

Data were analyzed using the Comprehensive Meta-Analysis version 2 program (Biostat, Englewood, NJ, 2004). Calculations were carried out for the whole group of articles depending on the binary data available for any AD: number of subjects and risk data (OR and RR with the corresponding 95% CI). Effect size was calculated based on studies that only showed the OR and respective 95% CI and the raw data from case-control and cohort studies. A second effect size was calculated independently with studies that only showed the RR and the respective 95% CI and the raw data from cohort studies. Different study designs were used to compute the same effect size since the effect size had the same meaning in all studies and were comparable in relevant aspects. Thus, this study was able to transform all values to log values (log odds ratio and standard error), which were used in the pooled analysis. This approach prevented the omission of studies that used an alternative measure.

A sensitivity analysis was done in which the meta-analysis results of the studies as a whole was compared to the same meta-analysis with one study excluded in each round to determine how robust the findings were. It was also done to evaluate the impact of decisions that lead to different data being used in the analysis and whether the conclusions reached might differ substantially if a single study or a number of studies were omitted.

Additional meta-analyses were done for studies with complex data structure and non-cumulative results if the information for the different effects was not totally independent. Thus, articles showing multiple independent subgroups within a study were considered in these analyses (i.e. different definitions of the disease, gender differences, toxic exposure or more than one comparison group within a study). To compare effects across subgroups we typically use subgroup as the unit of analysis in an independent meta-analysis.

Supplementary analyses were done for the association between each specific AD and OSs exposure. Additional analyses were also done grouping the data according to the exposure assessment category.

ORs were grouped by weighing individual ORs by the inverse of their variance. For each analysis, the final effect OR and 95% CI were obtained by means of both random and fixed effect models. The selection of the computational model was done based on the expectation that the studies shared a common effect size. The random effect model was preferred because it accepts that there is a distribution of true effect sizes rather than one true effect and assigns a more balanced weight to each study. It was also used because all the studies were considered to be unequal in terms of specific ADs.

Heterogeneity was calculated by means of Cochran's (Q) and Higgins's (*I*2) tests. The *I*2 test showed the proportion of observed dispersion that was real rather than spurious and was expressed as a ratio ranging from 0% to 100%. *I*2 values of 25%, 50%, and 75% were qualitatively classified as low, moderate, and high respectively. A significant Q-statistic (p<0.10) indicated heterogeneity across studies. Publication bias was determined using Funnel plots and Egger's regression asymmetry tests, and additional tests were applied if it was found.

## Results

**Figure 1:**
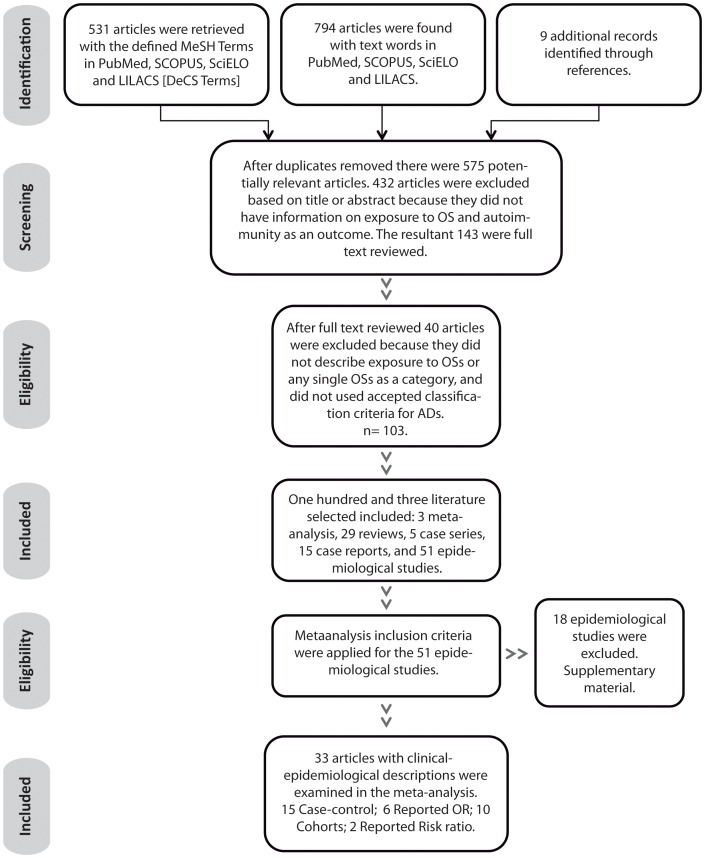
Systematic Review Results. Footnote: OSs: organic solvents; AD: autoimmune disease; OR: odds ratio.

The search with the defined MeSH Terms in PubMed, SCOPUS, SciELO, and LILACS [DeCS Terms] retrieved 531 articles. Using text words, 794 articles were found in PubMed, SCOPUS, SciELO and LILACS. Nine additional records were identified through references (Figure 1).

After duplicates were removed, there were 575 potentially relevant articles. Based on title or abstract, 143 were chosen for full text review. Six full text publications were not found and the authors of these publications or authors of publications that referenced them (e.g. [22]) were contacted. Of these, 4 articles were sent via e-mail [23]–[26] and one by post mail [27]. It was not possible to get full text access to one article [28]. One hundred and three articles described exposure to OSs as a category and used accepted classification criteria for ADs. Of these, 3 were meta-analysis [29]–[31], 29 were reviews, 5 case series [32]–[36], 15 case reports [37]–[51], and 51 epidemiological studies. Thirty-three of the epidemiological studies were finally included in the meta-analysis (Table 1) [7], [23], [26], [27], [52]–[80]. Because of lack of information, 18 epidemiological studies [25], [81]–[97] were not included in the meta-analysis (Table S1). Eight studies were not included because lack of information about the number of subjects with confirmed AD; two case-control studies were not included because lack of information about exposure in control subjects; one case-control study was excluded because control subjects had likely an AD diagnosis; three cohort studies were not included because lack of information about the number of unexposed and how many developed AD. Four studies were excluded because exposure data had low specificity for OS.

**Table 1:** Characteristics of the studies examined in the meta-analysis.

First author and year of publication	AD	Study population and control group	Assessment of exposure	Outcome	qBHs (max. score 21)
		**-Country**	**- Method used to get the information (questionnaire, database)**		
		**-Method used to identify cases and controls-Number (N).**	**- Terms of the exposure estimate (qualitative, semi-quantitative, quantitative indirect)**		
		**-Study design**	**-Type of exposure**		
Lundberg et al. 1994 [52]	AS RA	Sweden. From the computer-based census was extracted a cohort according to the occupational status ten years before the observation. N = 375,035 men and 140,139 women. Using the hospital discharge register the study population was observed from 1981–1983 and rheumatoid diseases cases extracted. N = 896 males and 629 females for RA; 79 males and 13 females for AS; for myositis 23 males and 14 females; for SSc 47 males and 24 females; 36 males and 57 females for SLE, 31 males and 6 females for vasculitis. Study design: retrospective cohort.	Job-exposure matrix with two categories of intensity for OS (semi-quantitative). Substantial use designated occupations where the average amount of solvent handled per year was estimated to 100 litters or more, e.g. house painters. Limited use designed occupations in which average amount of solvent handled was estimated 1–99 litters, e.g. house carpenters.	Increased relative risk for workers in occupations with substantial use of OS. Painters showed increased isk for AS.	16
Fored et al. 2004 [53]	GN	Sweden. Cases identified from the NPR (1996–1998) men and women whose creatinine level exceeded 300 mmol/L and 250 mmol/L, respectively, physicians at each hospital treating patients determined case eligibility by reviewing medical records N = 217. Control subjects randomly selected from the NPR and frequency-matched according to age and gender. Study design: population based case-control study.	Face to face interview by occupational hygienists (not blinded). Senior occupational hygienist estimated the intensity and cumulative lifetime exposure to OS using an accepted rating and arithmetic method (quantitative indirect). Seventy percent of the case patients and 62% of the control subjects reported exposure during manufacturing work, largest group: metal workers.	Adjusted OR 0.96 (95% CI 0.68–1.34)	12
Sesso et al. 1990 [54]	GN	Brazil. Cases: 17 patients with rapidly progressive GN associated with OS exposure and 34 matched hospital controls. Renal histologic findings suggest immune complex mediated injury. Study design: Case-control.	Self-report OS exposure (qualitative) was defined as 1 hour or more weekly for 3 consecutive months or longer. Increased risk was detected in exposed to fuels.	Relative risk = 5 (95% IC 1.14–22)	15
Flodin et al. 2003 [55]	MS	Sweden. Cases were identified among The Nurse Union through an appeal published in the union magazine. The occupational group they focused was Nurse anesthetists. Among the subjects who replied, Nurse anesthetists with MS diagnosis were identified. To confirm MS medical files were requested N = 10. Study design: retrospective cohort.	A questionnaire requested qualitative information about years and kind of work tasks, and type of anesthetics they had used.	Increased MS risk was detected in nurse anesthetists.	17
Stenager et al. 2003 [26]	MS	Denmark. In a nationwide study on the isk of MS in Danish nurses were identified nurse anesthetists with confirmed cases of MS. Expected number of MS was calculated in an age- and sex-matched population. Study design: retrospective cohort.	Qualitative data was assessed using a national register of nurses and the Danish Multiple Sclerosis Register.	Nurse anesthetist had no increased risk	11
Amaducci et al. 1982 [56]	MS	Italy. Cases were identified from a register of MS patients and interviewed to establish their last occupation before the disease onset. Five cases of MS were employed in shoe and leather industry. Relative risk was calculated between shoe and leather workers and the general population of Florence. Study design: retrospective cohort.	Data on general population and people employed in the shoe and leather industry of Florence were obtained from Census.	Increased risk among shoe and leather industry workers (4.81)	13
Nelson et al. 1994 [57]	MS	USA. Cases identified by reviewing medical records of employees taking medical disability at any of eight automobile assembly plants located in Michigan and Ohio N = 20. Controls chosen from employees who took disability retirement for disorders other than those of interest. Study design: Case- control.	Semi-quantitative exposure indices were assigned by two industrial hygienists and expressed in years working in solvent exposed tasks.	OR 2.0 (95% CI 0.6–6.9)	15
Grønning et al. 1993 [58]	MS	Norway. Cases were patients living in Hordaland with clinical MS onset between 1976–86 N = 139. Controls comprised patients admitted to the hospital with other diagnosis matched on age, sex and residence N = 161. Distributions among type of work and type of OS were similar among cases and controls. Study design: case- control.	Face to face questionnaire that elicited detailed information about exposure to OS and number of years of exposure. Exposure index was calculated using Ravnskov formula (quantitative indirect). Main occupational exposure to OS occurred at mechanical, oil, textile, wood-working, paint, printing, plastic and rubber industries.	OR 1.55 (95% CI 0.83–2.90)	15
Zorzon et al. 2003 [59] ^bc^	MS	Italy. Cases were consecutive patients who were being seen in routine follow-up at the Multiple Sclerosis Center of the University of Trieste N = 140. Control group was formed by sex and age matched blood donors N = 131. Controls were similar to cases in area of residence and ethnicity. Study design: case- control.	Face to face interview (blinded) collected detailed information related to occupational exposures (qualitative). Exposure was considered when lasting >5 years.	OR 0.8 (95% CI 0.5–1.4)	14
Juntunen et al. 1989 [60]	MS	Finland. From the Finish Twin Cohort 21 cases of MS were extracted and records reviewed by a neurologist. Only 2 pairs were concordant. The comparison group was formed by the co-twins. Study design: Nested case control.	Face to face interview by a specialist on occupational medicine. Exposure was estimated and roughly classified (semi-quantitative).	No statistically significant association of exposure.	13
Flodin U et al. 1988 [27]	MS	Sweden. Cases were obtained from the patient files of the neurologic clinics of the General Hospital of Linköping N = 83. Control group was already available as primarily utilized for cancer studies, and randomly drawn from the population registers N = 467. Study design: Case- control.	Mailed questionnaire. A minimum criterion of one year was required for exposure time. Semi-quantitative score was obtained based on Ravnskov method.	Mantel-Haenezel Rate Radio 2 (90% CI 0.9–4)	14
Koch-Henriksen et al. 1989 [61]	MS	Canada. Case group N = 187. Control group N = 187. Case-ascertainment and selection of controls: NA. Study design: population based case-control study.	Questionnaire about industrial OS exposure (qualitative).	No significant association	14
Casetta et al. 1994 [23]	MS	Italy. Cases: definite MS patients living in the province of Ferrara N = 104. Two matched control groups, one from hospitalized patients and the other from general population. Study design: Case- control.	Face to face blind interview about occupational history before the age of onset (qualitative).	OR 4 (95% CI 1.48–11.11)	14
Landtblom et al. 1993 [62]	MS	Sweden. Cases collected from the files of the neurological department of the hospitals Jönköping and Kalmar N = 91. Controls were randomly drawn from population registers of the administrative provinces of Jönköping and Kalmar N = . Study design: population based case-control study.	Mailed questionnaire focused on occupational exposures. A minimum criterion of one year was required for exposure time. Semi-quantitative score was obtained based on Ravnskov method.	Mantel-Haenszel Rate Radio 2.8 (95% CI 0.9–8)	15
Landtblom et al. 2006 [63]	MS	Sweden. From the 1985 census were identified nurse anesthetists (N = 907) and compared to other nurses (N = 39,703) and teachers (N = 20,053). Age restriction to the interval of 35–50. Cases: MS diagnosis or disability pension due to MS (N = 168). Study design: retrospective cohort.	In the census each person had to declare which occupation held. Based on this information nurse anesthetists, potentially exposed to OS, were identified (qualitative).	Cumulative incidence rate ratio was increased in female nurse anesthetists (statistically no significant)	15
Mortensen et al. 1998 [64]	MS	Denmark. From the 1970 census “solvent-exposed men” group was extracted N = 124,766. “Unexposed men” cohort comprised electricians, bricklayers, and butchers N = 87,501. After a 20 years of follow-up 87 MS cases presented among presumed to be exposed. Study design: cohort.	Census contains information of the occupational status and qualitative data of exposure. House painters, carpenters, and typographers are the occupations with the longest exposure.	There was no important difference in the standardize morbidity ratio between occupational groups.	14
Riise et al. 2002 [65]	MS	Norway. Three cohorts of 11,542 painters; 36,899 construction workers and 9,314 food-processing were followed from 1970 to1986. A total of 9 painters, 12 construction workers and 6 food-workers received pension because of MS. Study design: cohort.	Workers potentially exposed to OS (qualitative data) were identified by the 1970 national census.	The relative risk 95% CI for painters compared with workers not exposed to OS was 2	15
Riise et al. 2011 [66]	MS	Norway. From the Registry of Employers and Employees was extracted a cohort of 27,900 offshore petroleum workers, 42,657 onshore petroleum workers and 365,805 referents. The total cohort was linked to the nationwide Norwegian MS registry including all cases of MS with onset after the start of their working engagement. Study design: retrospective cohort.	Offshore workers of the petroleum industry with suspected OS exposure (qualitative data) included technicians, laboratory engineers and control operators involved in the production process and ‘drilling and well maintenance offshore’.	There was no increased risk of MS among offshore workers	12
Gershwin et al. 2005 [67]	PBC	USA. Between November 1999 and June 2004 a total of 1,090 patients with PBC were refereed from 23 referral medical centers. Referring physicians re-evaluated anonymized clinical information in 100 randomly selected enrolled patients. Controls were selected by random digit dialing and matched for sex, age, race, and geographical area. Study design: case-control.	Telephone interview included over 180 questions and 300 sub-questions, including exposure data (semi-quantitative). PBC patients reported to use hair dye 38±50 times/year and nail polish 29±65 times/year.	A history of nail polish was associated with PBC AOR 1.002 (95% CI 1.00–1.003).	15
Lane et al. 2003 [68]	PSV	UK. Cases: PSV diagnosed between May 1988 and july 2000 identified from a prospective vasculitis register. Case notes were reviewed for clinical and laboratory details N = 103. Controls N = 220 hospital inpatients and outpatients with non-inflammatory musculoskeletal disease, matched to the age of the case at the time of interview. Controls were excluded if personal history of AD. Study design: case –control.	Face to face structured questionnaire. To defined occupational OS exposure was used the Steenland job exposure matrix (quantitative indirect).	A history of OS was associated with PSV; OR 2.7 (95%IC 1.1–6.6) and WG 3.4 (1.3–8.9)	15
De Roos et al. 2005 [69]	RA	USA. The Agricultural Health Study is a cohort of licensed pesticide applicators and their spouses. From this cohort RA diagnosis were validated among women who had self-reported RA, N = 136 physician-confirmed cases were matched by age to 5 controls. Controls were selected from among women in the cohort except those who had reported any AD. Study design: Nested case-control.	Information was available from the Agricultural Health Study questionnaires (qualitative).	Risk of RA was not associated with OS; OR 0.6 (95% CI 0.3–1.5)	14
Purdie et al. 2011 [70]	RD	New Zeeland. Technicians, scientist, and laboratory assistants working in medical laboratories were assessed for RD using the UK Scleroderma Study group questionnaire. RD rates in solvent-exposed histology, cytology, and transfusion medicine (N = 301) were compared with unexposed medical laboratory workers from transfusion medicine. Study design: retrospective cohort.	Laboratory workers self-report history of exposure to OS including frequency and years working (semi-quantitative). Higher rates of RD, particularly, for those who had worked with xylene, toluene or acetone.	OR 8.8 (95% CI 1.9–41.1)	16
Cooper et al. 2004 [71]	SLE	USA. Cases: Carolina Lupus Study included resent diagnosed patients identified through community based rheumatologist practices in the study area N = 265. Population based controls were identified through driver license records and were frequency matched to the cases by age, sex, and state N = 355. Study designed: case-control.	In 60 minutes in person interview, job history and specific questions about potential exposure were asked. Three reviewers (one industrial hygienist and 2 epidemiologist) reviewed the job and task description and qualified the exposure in 5 categories (likely-high, possible-high, possible-moderate, indirect, none).	There was no evidence of association between occupational exposure to OS and SLE	11
Cooper et al. 2010 [72]	SLE	Canada. Cases: recruited from 11 rheumatology centers across Canada N = 258. Control randomly selected from phone number listings and matched by age, sex, and area of residence N = 263. Study design: case-control.	30–45 minutes telephone interview assessed work history and specific potential exposures (qualitative). Strong associations were seen with work as an artist working with paints, dyes or developing film (1), and applying nail polish (2).	(1) OR 3.9 (95% 1.3–12.3)(2) OR 10.2 (95%CI 1.3–81.5)	15
Finckh et al. 2006 [7]	SLE	USA. cases: women with SLE were identified through both community screening and hospital data-bases in 4 predominant African American neighborhoods N = 95. Controls were female residents of the same area who participated in one of the screening events but were negative for SLE N = 191. Study design: population based case-control.	Data were collected using in-person interviews (qualitative). A detailed lifetime work history followed by a structured checklist involving specific jobs likely to involve exposure to solvents (e.g. wood finishes, paints, dry-cleaning, pottery).	OR 1.04 (95% 0.34–3.2)	13
Nietert et al. 1998 [73]	SSc	USA. Cases: From March 1995 to February 1997 were included patients being seen in the rheumatology clinics N = 178. Controls diagnosed with osteoarthritis, gout and fibromyalgia were selected during the same time period N = 200.Study design: case-control.	Data were collected using in-person nterviews. Semi-quantitative score were assigned by computerized method, based on the occupational and industrial classification.	OR 2.9 (95% CI 1.1–7.1)	15
Bovenzi et al. 1995 [74]	SSc	Italy. Cases: from the computerized admission files of all of the local hospitals from 1976 to 1991 N = 21. Clinical records were obtained and verify. With the same database system, for each case, 2 age and gender matched referents. Study design: case-control.	Blinded interview with a structured questionnaire, which included exposure to OS. The subjects were allocated into the various exposure categories (semi-quantitative) by 2 occupational physicians who were blinded. Minimum criterion of six months was required for exposure duration.	OR 9.28 (95% CI 0.48–74.1) for men; 2.11 (0.20–22.0) for women	16
Bovenzi et al. 2004 [75]	SSc	Italy. Cases: N = 55 (46 female, 9 male) recruited at Institute of Internal Medicine of University of Hospital Verona, patients affected with localized scleroderma were not included. Controls N = 171 among patients admitted in the same study period to the Institute of Orthopedics with other diagnosis different to any AD. Study design: case-control.	In-person interview and structured questionnaire focused on occupational history. Explicit questions referenced to potential exposure to OS. The occupational history was reviewed and qualified (semi-quantitative) by an expert in industrial hygiene and occupational health, blind to case-control condition.	Female teachers OR 3.4 (95% CI 1.2–10.1) Textile workers OR 2.1 (95% CI 1.0–4.6)	14
Diot et al. 2002 [76]	SSc	France. Cases admitted consecutively to the department of Internal Medicine from 1998 to 2000 N = 80. For each case, two age, gender, and smoking habits (frequency and quantity) matched controls. Study design: case-control.	A semi-quantitative score were calculated, by a committee of six experts, based on probability, intensity, daily frequency, and duration of exposure for each period of employment. Final cumulative score was obtained.	Significant associations were observed for TCE, toluene, aromatic solvents, ketones, white spirit, epoxy resins and welding fumes.	15
Maître et al. 2004 [77]	SSc	France. Cases: 10 men and 83 women diagnosed between 1995 and 1999 and 206 age and sex matched controls, randomly selected from general population. Study design: case-control.	Mailed questionnaire and phone interview. The questions included occupational details and suspect forms of exposure. Every job was classified by a group of experts according to the potential level of exposure on a 3-level scale (semi-quantitative). The criteria used for this classification were likelihood, duration, intensity and percentage of working time exposed.	Solvents were linked to SSc OR 3.2 (95% CI 1.5–6.6) in both men and women	15
Thompson et al. 2002 [78]	SSc	Canada. Cases: N = 91 patients identified in a rheumatology outpatient practice. Controls N = 154 derived from the same practice, same rheumatologist, and matched by age and sex, did not have SSc. Study design: case-control.	Mail questionnaire with specific questions of employment information and chemical exposure (semi-quantitative) at workplace or home. Exposure rates in both groups were low.	There were no differences in type of employment between the 2 groups.	11
Silman et al. 1992 [79]	SSc	UK. Cases recruited from registers of patients with SSc from different centers, N = 56 men. Controls: (1) patients were asked to provide three male friends within 5 years of age and (2) general practitioner was approached to provide the names of three male patients, age matched. Study design: case-control.	Postal questionnaire for self-report exposure. Occupational exposure was assessed in two ways. First, detailed history of occupations was assessed, blinded to the case or control status, by an experienced occupational hygienist and scored (semi-quantitative) for nil, possible, or probable exposure. Secondly the subjects were asked to recall exposure to a detailed list of solvents.	No significant increase in exposure to OS was noted.	13
Czirják et al. 1989 [80]	SSc	Hungary. Cases from hospital data N = 61, with controls matched for age and sex. Study design: case-control.	Self-reported exposure (qualitative) to: benzene, isopropyl alcohol, ethyl acetate. Risk related to length of exposure but not data on risk by intensity.	OR 23.18 (95% CI 2.97–180.79)	17

AD: autoimmune disease, qBHs: Quantitative score of the Bradford Hills Criteria for causation. AS: ankylosing spondylitis. RA: rheumatoid arthritis. SSc: systemic sclerosis. SLE: systemic lupus erythematous, OS: Organic solvent. GN: glomerulonephritis,NPR: National population Register; N: number of subjects in each group. OR 95% CI: Odds ratio with 95% confident interval. MS: multiple sclerosis. NA: not available data. PBC: primary biliary cirrhosis. AOR: Adjusted odds ratio. PSV: primary systemic vasculitis, WG: Wegener's granoulomatosis. RD: Raynaud's disease. TCE: trichloroethylene.

Types of exposure and exposure assessments are described in table 1 for each study. The average qBHs for the total publications included in the meta-analysis was 14.25 points (SD, 1.586; range, 11–17 points; 99% CI, 13.528–14.972) reflecting a categorical rating of moderate relationship.

**Figure 2:**
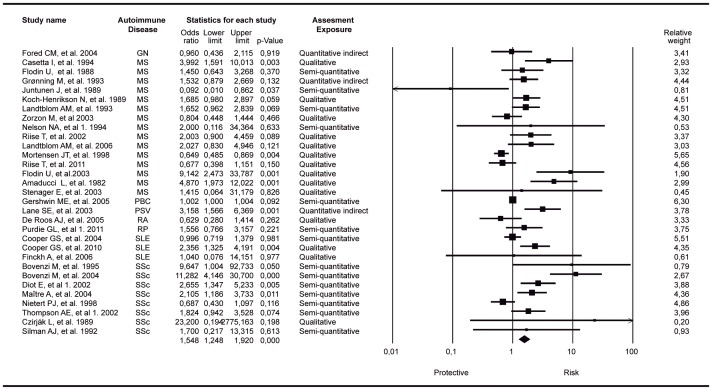
Forest plot of studies meta-analyzed: association between organic solvents and autoimmune disease as a trait. Footnote: final common effect size based on a random model. Odds Ratio (95%CI) with raw data from case control and cohort designed studies were included. The most relevant outcome per author was included. The relative weight of each study is included. GN: glomerulonephritis; MS: multiple sclerosis; PBC: primary biliary cirrhosis; PSV: primary systemic vasculitis; RA: rheumatoid arthritis; RP: Raynaud disease; SLE: systemic lupus erythematosus; SSc: systemic sclerosis. Diot, et al 1: organic solvent as a whole; Thompson AE, et al 1: turpentine exposure (the most significant result); Nelson NA, et al 1. 1994: disabled population; Purdie GL, et al 1. 2011 confirmed RP population.

We found a significant association between OSs exposure and the increased risk of developing an autoimmune trait by evaluating all ADs as a single group. Figure 2 shows the forest plot corresponding to the meta-analysis including the most relevant outcome per author where the final common effect size based on a random model was statistically significant (OR: 1.54; 95% CI: 1.25–1.92; p-value<0.001). The results of different measures for heterogeneity calculated for the analysis showed in Figure 2 were as follows: Q-value: 132.1; degree of freedom (Q):30; p-value<0.0001; *I*-squared: 77.3%; Tau-Squared 0.19. The relative weight of each study is included in the forest plot (Figure 2)

There were 5 studies showing complex data structure with different and non-cumulative results where the information for the different effects was not totally independent [52], [57], [70], [76], [78]. Then, 22 additional meta-analyses including 30 articles and the different outcomes of four of the above mentioned studies were calculated independently [57], [70], [76], [78]. These analyses included five from Diot et al. 2002 [76] (different toxic exposure measured: chlorinate, ketones, aromatic, toluene, TCE), one from Nelson et al. 1994 [57] (control not disabled population) and Purdie et al. 2011 [70] (a different cutoff point to disease criteria) and fifteen from Thompson et al. 2002 [78] (different toxic exposure measured: toluene, benzene, white spirit, perchlorethylene, TCE, trichlorethane, vinyl chloride, urea formaldehyde, meta-phenylenediamene, bicromade, aromatic hydrocarbons, aliphatic hydrocarbons, fenfluramine, diethylpropion, L5 OH-tryptophan). In these meta-analyses, the studies that provided uniquely RR data were not included [52], [54] for statistical reasons. All these additional meta-analyses showed a significant association between the exposure to OSs and ADs as a trait (Figures S1, S2, S3, S4, S5, S6, S7, S8, S9, S10, S11, S12, S13, S14, S15, S16, S17, S18, S19, S20, S21, S22). After doing a sensitivity analysis excluding one study at a time, the results were similar to the cumulative analysis (Figures S23 and S24).

A second effect size was calculated based on data from two studies showing RR data [52], [54] with raw data from cohort studies [26], [55], [56], [63]–[66], [70]; this effect size was not significant (OR: 1.62; 95% CI: 0.99–2.65; p-value:0.051) (Figure S25). The results of different measures for heterogeneity calculated for the analysis showed in Figure S25 were as follows: Q-value: 42.01; degree of freedom (Q):11; p-value<0.001; *I*-squared: 73.8%; Tau-Squared 0.40.

**Figure 3:**
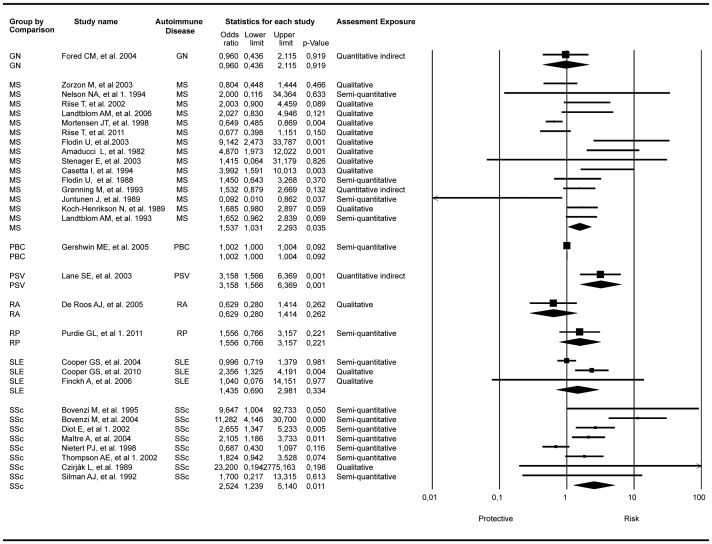
Forest plot of studies meta-analyzed grouping by comparison of specific autoimmune diseases. Footnote: random effect model showing significant association between SSC and OSs exposure. PBC and PSV included only one study (100% of the weight). Q value for SSc analysis: 33.7, *I* ^2^:79,2, Degree of freedom (Q):7, p-value<0,0001. GN: glomerulonephritis; MS: multiple sclerosis; PBC: primary biliary cirrhosis; PSV: primary systemic vasculitis; RA: rheumatoid arthritis; RP: raynaud disease; SLE: systemic lupus erythematosus; SSc: systemic sclerosis. Diot, et al 1: organic solvent as a whole; Thompson AE, et al 1: turpentine exposure (the most significant result); Purdie GL, et al 1: confirmed RP population; Nelson NA, et al 1. 1994: disabled population.

Additional analysis limited to the association between each specific AD and OSs exposure presented significant associations in the random model. For MS, the OR was 1.53 with 95& CI 1.03–2.29 and p value: 0.035, with fifteen studies included. For primary systemic vasculitis (PSV), the OR was 3.15 with 95% CI: 1.56–6.36 and p-value: 0.001, with one study included in the cumulative analysis for this disease. Systemic sclerosis (SSc) showed these results OR: 2.54; 95% CI: 1.23–5.14; p-value: 0.011, with eight studies included (Figure 3). Primary biliary cirrhosis (PBC) was positively associated but not statistically significant (OR: 1.002; 95% CI: 1–1.004; p-value: 0.092).

The analyses according to the exposure assessment category are shown in figure S26. There were three groups included. Two of them were not significant: qualitative (OR: 1.29; 95% CI: 0.84–1.98; p-value 0.231) and quantitative indirect (OR: 1.69; 95% CI: 0.90–3.16; p-value 0.101) and one was significant: semi-quantitative [(OR: 1.48; 95% CI: 1.17–1.87; p-value 0.001). Heterogeneity: Q-value: 56.7; degree of freedom (Q):13; p-value<0.0001; I-squared: 77%; Tau-Squared 0.19]. The total heterogeneity between the three groups for the random effects analysis was not significant [Q-value: 0.60; degree of freedom (Q):2; p-value: 0.741]

Evidence of significant publication bias was identified using the Egger test (p-value 2-tailed: 0.002; intercept: 1.09) for the meta-analysis which included studies that report OR with its respective 95% CI and raw data from case control and cohort designed studies. The Funnel plot showing the standard error on the Y axis is shown in Figure S27. Therefore, a second analysis was run in a search for publication bias. The classic fail-safe analysis indicated that 279 missing studies would give a p-value of >0.05. Begg and Mazumdar rank correlation was not significant (p-value 2-tailed: 0.16) and the trim and fill adjustment did not suggest a lower risk than the original analysis [adjusted values (11 studies trimmed) point estimate 1.03 (0.83–1.28), Q value: 227]. Based on all the analyses for publication bias, we consider the impact of bias in the present meta-analyses trivial.

Since 1977, 20 publications including case-reports and case series (Table S2) have reported 37 cases of AD possibly being triggered by OSs. We also found 3 previous meta-analyses. The first was published in 1996 by Landtblom AM et al [29] and concerned OSs exposure as a cause of Multiple Sclerosis (MS). They found 13 studies and reported an overall RR of 1.7 (with a 95% CI of 1.1–2.4). Later in 2001, Aryal BK et al [30] published a meta-analysis of SSc and solvent exposure. Eight studies met inclusion criteria, and the RR was reported to be 2.91 (with a 95% CI of 1.60 to 5.30). Kettaneh A et al in 2007 [31] published the most recent meta-analysis about occupational exposure to solvents and gender-related risk of SSc, they found a statistically significant association of SSc with OS exposure (OR 2.4; 95% CI 1.7–3.4: P = 0.002) and concluded that whereas SSc affects women predominantly, among subjects with occupational exposure to OS, men are at a higher risk of developing the disease than women. All the studies included in these publications were examined in our analysis. No meta-analysis evaluating ADs as a trait was found.

Regarding the systematic search for the OSs molecular mechanisms related to responses of immune system and ADs, with defined MeSH Terms and text words, retrieved 893 articles. After duplicates were removed, we obtained 827 articles of which 86 were included according to the inclusion criteria. The results are described in detail in Tables S3 and S4 and inclusion/exclusion criteria are described on Figure S28. Table 2 shows selected articles, representing main molecular processes related with OSs exposure and their potential implication on immune system or autoimmune pathologies. We found that the effects of OSs on the immune system include lymphoproliferation, autoantibody production, Th1 and Th17 responses, oxidative stress, protein modification as well as effects on gene expression.

**Table 2:** Effects of the exposition to organic solvents on experimental models.

OS	EFFECTS ON PHENOTYPE	REFERENCES
TCE - DCAD	Lymphoproliferative reaction, ANAs and anti-cardiolipin autoantibodies production (Mouse).	[100]
TCE, PCE	Protein modification, TCE binds covalently with some proteins or lipids and form adducts (Rat, Mouse).	[101] [102] [103] [104] [105]
TCE	Splenocytes of mice treated with TCE secreted higher levels of IL-17 and IL-21. Protein adducts stimulate the activation of Th17 cells.	[125]
TCE	Production of ROS and NO, activation T cells; cellular infiltration (Mouse, Human Keratinocytes).	[109] [111] [126]
TCE	The low exposure to TCE generates a Th1-like cytokine responses and ANAs production; High-Exposure, increase CD44high T cells subsets with ability of IFN-γ secretion and cellular infiltration (Mouse).	[107]
TCE	Increase of CD4+ and CD8+ T cells population (Mouse).	[110]
TCE	Inhibition of cellular apoptosis of naive CD4+ and CD8+ T cells subset; anti-histone autoantibodies production (Mouse).	[111]
TCE-TCAH	Inhibition of lymphocyte apoptosis in the thymus through decrease FasL or peripheral lymphocyte (Mouse).	[112]
TCE + HgCl2	Anti-Hsp90 autoantibodies and antibodies against liver proteins production (Mouse).	[114]
TCE−DCVC	High-doses cause cellular necrosis. Low-doses produces changes in the transcription of several genes involved in apoptosis and cellular proliferation (Human).	[115]
TCE	Low-doses cause DNA-hypermetylation on cardiac myoblasts (Rat).	[116]
Benzene	Decrease of T cells population (cellular immunity) (American kestrels Birds).	[117]
Benzene	Decrease in number of CD4+ and CD8+ T cells, B cells, granulocyte and platelets (Human).	[118]
Benzene	Increase of ROS production and induction of DNA-fragmentation (Human).	[119]
Benzene	Changes in gene transcription involved in apoptosis, oxidative stress, cellular cycle and cytokine production (Mouse).	[121]
Toluene	High-doses affect the IFN-γ, IL.-4 and IL-13 production by T cells and increases TNF-α expression (Human PBMCs).	[122]
Toluene-hexane- Methyl ethyl Ketone	Produce oxidative stress (Human Jurkat Cells).	[123]
TCE –Benzene- HgCl2	Changes in gene expression with effects on cellular proliferation, apoptosis and tissue-specific function (Rat).	[124]

In parenthesis is shown the model where the effect was studied. OS: Organic solvent; TCE: Trichloroethylene; DCAC: dichloroacetyl chloride; TCAH: Trichloroacetaldehyde hydrate; HgCl2: Mercuric Chloride; DCVC: dichlorovinyl-l-cysteine; PCE: Perchloroethylene; ROS: Reactive Oxygen Species; NO: Nitric Oxide; ANA: Anti-Nuclear Antibodies; TNF-α: Tumor Necrosis Factor alpha; IFN-γ: Interferon Gama; IL-4: Interleukin 4.

## Discussion

Our results indicate that OS exposure is a risk factor for developing ADs. Even though the individual meta-analyses (i.e. each AD considered separately) disclosed significant association for MS, PSV and SSc (Figure 3), the direction and significance of this association did not change when all ADs, considered as a single trait, were analyzed (Figure 2).Different combinations of factors involved in the generation of autoimmunity produce diverse clinical pictures within the wide spectrum of ADs (mosaic of autoimmunity) [2]. Our study, which takes into account both OSs as a whole and each solvent separately, reinforces this as well as the fact that ADs might share several common mechanisms(i.e., the autoimmune tautology) [98]. However, the term “separately,” which is used to refer to the studies that analyze only one solvent, is not the most biologically appropriate because most of the solvents are a mixture [99].

Our meta-analysis with ORs as the measure of association including 31 articles regarding 8 ADs showed a significant relationship of OSs exposure with ADs (OR: 1.54; 95% CI: 1.25–1.92; p-value<0.001) and that with RRs as the measure of association including 10 articles and 5 ADs showed a near significant relationship (OR: 1.62; 95% CI: 0.99–2.65; p-value:0.051). When each AD was considered individually, there were also significant results with MS, PSV, SSc and PBC, although the latter was positively associated but not statistically significant.

A systematic and comprehensive review of the effects of OSs on the immune system is shown in Table 2. OSs are capable of altering cellular proliferation, apoptosis and tissue-specific function [100]–[126]. Both the amount and duration of OSs exposure are essential in pathology causation. Chronic exposure to OSs might lead to deposits in an organ and consequently to immune infiltration, similar to what is observed in ADs. The self-proteins that are modified by OSs may become immunogenic, recognized as foreign, and then initiate an inflammatory response and tissue injury. In this regard and according with our results, there are similar pathways operating on the incidence of ADs, but there are also specific mechanism that could lead to the particular manifestations of each AD; for instance, lymphocyte infiltration and immunoglobulin's deposits in SLE, and enzymatic alteration and scleroderma-specific antibody subsets in SSc [86], [106], [109].

Ketones are the most common OS used by the general population. Acetone, the simplest example of the ketones, is a commonly used solvent and is the active ingredient in nail polish remover and some paint thinners. It has been suggested that nail polish use may be associated with PBC [67]. These data are intriguing in view of the xenobiotic hypothesis proposed for the development of PBC with specific halogenated compounds. These compounds could increase the immunogenicity of mitochondrial proteins and induce anti-mitochondrial antibodies in animal models [127]. In fact, only one clinical study was included in the meta-analysis regarding PBC and nail polish exposure disclosing a positive associated but not statistically significant [67]. More studies involving PBC patients searching for this association could be useful.

Long term exposure to OSs seems to foster massive hepatic mononuclear infiltration leading to autoimmune hepatitis although it is important to highlight that this infiltration is the first step in the immunopathogenesis of not only autoimmune hepatitis but also the rest of the ADs [108]. As shown by Cai et al [109], lymphocyte infiltration was found in the pancreas, lungs, and kidneys in addition to the liver.

In autoimmune thyroid disease, it is probable that solvents may interfere with iodine transportation and induce oxidative stress that leads to an inflammatory response to the thyroid gland [128].

The relevance of our results rely in the fact that relation between SSc and environmental exposure, especially involving OSs is significant. Mice MRL+/+, an autoimmunity susceptible strain, when exposed to TCE increase the total IgG serum concentration, antinuclear antibodies (ANAs) and anticardiolipin autoantibodies [100]. On the other hand, in an in vitro model of human epidermal keratinocytes, was possible to determinate that TCE not only stimulates reactive oxygen species release, but also it stimulates nitric oxide synthesis by nitric oxide synthase. These cellular changes may contribute to the physiopathological process that lead to skin injury such as shown in SSc [106]. The biological mechanisms by which OSs may induce the development of ADs support the results observed trough the meta-analysis.

Concerning MS, when an independent analysis was done for each disease, MS show a significant association with OSs exposure. These results are like those reported by Landtblom et al [29], in their 1996 meta-analysis. Landtblom et al implemented a Mantel-Haenszel RR calculation. The main differences between their analysis and ours rely on the statistical approach because the Mantel-Haenszel method for combining OR is an alternative to the fixed-effect inverse variance method and we developed a random effect model. Our meta-analysis included 15 MS studies, 7 new to the previous meta-analysis [26], [55], [59], [63]–[66] published between 1994 and 2012.

**Figure 4:**
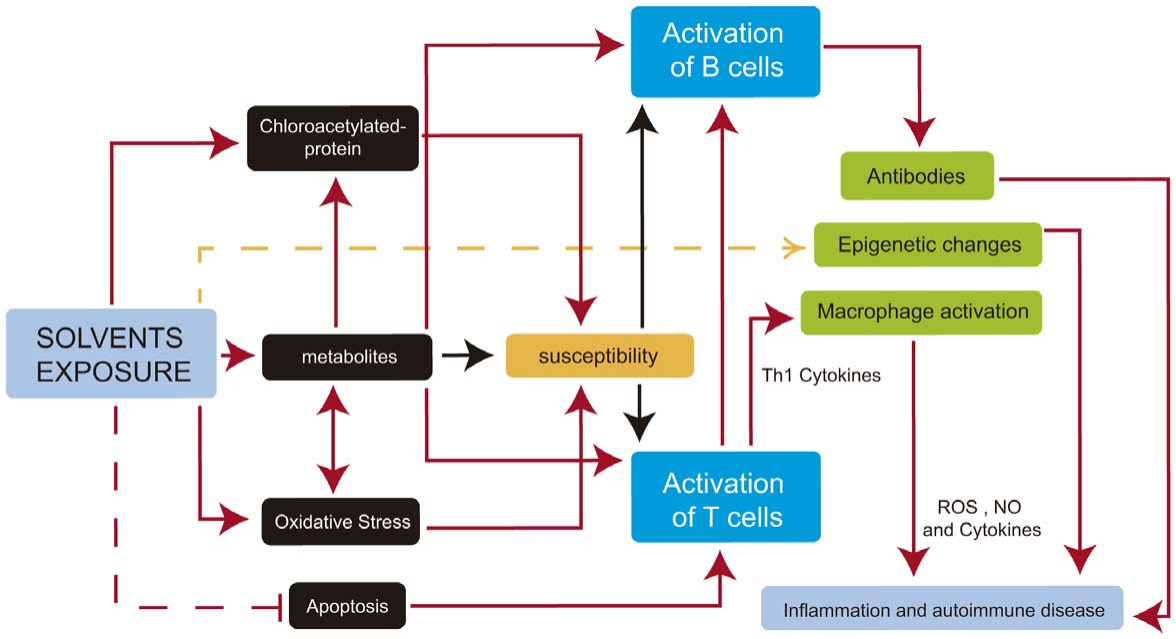
Potential molecular mechanisms implicated in solvent autoimmune disease development. Footnote: Solid red arrows represent known paths. Yellow dashed arrow represents hypothetical mechanisms (warranting future research), and red dashed line represents an inhibited process. In susceptible individuals, activation paths are stronger (black arrows). See text for details. ROS: Reactive oxygen Species; NO: Nitric Oxide.

The precise mechanisms responsible for the development of environmentally-induced autoimmune disorders are unknown. Although many hypotheses for the occurrence of autoimmune phenomena after various environmental exposures have been proposed, none of the hypotheses is completely supported by direct causal evidence. Also, mechanisms thought to be involved in the initiation of the disease process might differ from the mechanisms believed to exacerbate an established illness. However, the experimental approaches have been able to identify different environmental factors that use the same toxicity paths and mechanisms and either individually or jointly can have strong effects on molecular signaling pathways, immune responses or regulation mechanisms actively involved in health and disease (Figure 4 and Table 2).

It could be suggested that, as described for autoimmune/inflammatory syndrome induced by adjuvants, the toxic effect influences the appearance of these conditions only in subjects who are genetically susceptible [13].

### Study Limitations

Significant differences between case-control and cohort models were found. This fact can be explained by the limitations of each of these methodologies [129]. The following are the limitations in case-control studies. (I) the information about exposure is primarily based on interviews and may be subject to recall bias. (II) Validating the information on exposure can be difficult or even impossible. (III) By definition, case-control studies evaluate a single disease. (IV) The selection of an appropriate control group might be difficult. Most of the studies ignored the common origin of ADs and this generates the possibility of including patients with an underlying autoimmune condition as controls.

Most of the cohort studies included in our meta-analysis were retrospective. This implied that: (I) data was collected before the research hypothesis was defined leading to inaccurate data for the research. (II) The crude information was taken from databases or census. Therefore, the report on the exposure is not a direct quantification of the exposure. (III) The outcome information came from databases or medical records, but the subjects were not examined and this can lead to misdiagnosis. The explanation for why the result in the meta-analysis that included studies that reported the RR and raw data from cohort studies was not significant could be based on the abovementioned information as well as the low power due to a small sample size.

Exposure misclassification is a major problem when assessing the roll of environmental factors in complex diseases. Most individuals are not aware of the specific agents to which they have been exposed, and databases do not provide further information. None of the studies included in this meta-analysis employed an objective method of exposure assessment. Only two studies retrieved in this search [83], [85] reported a direct-quantitative measure of exposure (i.e. TCE in concentrations from 6 to over 500 parts-per-billion [83]). After performing the analyses according to the exposure assessment category (figure S26) the final common effect size remained significantly associated as risk factor.

A significant effort is necessary to determine the proper way to test the causal factors for autoimmunity. Nevertheless, we believe that identifying the causal pathways of toxics already known to be associated with generating autoimmunity is a breakthrough. Standardizing the pathways as validated biomarkers would lead to more accurate studies. Future research on environmental exposure will enhance our knowledge of the common mechanisms associated with ADs.

In conclusion, an association between OSs exposure and ADs was observed. This approach could be applied to any study of the association between exposure to other toxics and ADs. Although OSs exposure has not yet been sufficiently investigated, in order to clarify their roles in ADs pathogenesis, there is a need to study their relationship with genes associated, whether involved in protection or susceptibility to each AD and their effects on development of the autoimmune process.

## Supporting Information

Figure S1 **Forest plot of supplementary meta-analyses.** Footnote: final common effect size based on a random model. Odds Ratio (95%CI) with raw data from case control and cohort designed studies were included. Studies that provided uniquely RR data were not included for statistical reasons. Each different outcome of the studies with complex data structure was included.GN: glomerulonephritis; MS: multiple sclerosis; PBC: primary biliary cirrhosis; PSV: primary systemic vasculitis; RA: rheumatoid arthritis; RP: Raynaud disease; SLE: systemic lupus erythematosus; SSc: systemic sclerosis. The complex data structure and non-cumulative results of articles showing multiple independent or dependent subgroups included in the analysis was S1 Diot E, et al.2 2002 Exposition to chlorinate.
(TIF)

Figure S2 **Forest plot of supplementary meta-analyses.** Final common effect size based on a random model. The studies included and abbreviations are the same as in Figure S1 with the exception of Diot E, et al.3 2002. Exposition to ketones.
(TIF)

Figure S3 **Forest plot of supplementary meta-analyses.** Final common effect size based on a random model. The studies included and abbreviations are the same as in Figure S1 with the exception of Diot E, et al.4 2002. Exposition to aromatic.
(TIF)

Figure S4 **Forest plot of supplementary meta-analyses.** Final common effect size based on a random model. The studies included and abbreviations are the same as in Figure S1 with the exception of Diot E, et al.5 2002. Exposition to toluene.
(TIF)

Figure S5 **Forest plot of supplementary meta-analyses.** Final common effect size based on a random model. The studies included and abbreviations are the same as in Figure S1 with the exception of Diot e, et al.6. Exposition to TCE.
(TIF)

Figure S6 **Forest plot of supplementary meta-analyses.** Final common effect size based on a random model. The studies included and abbreviations are the same as in Figure S1 with the exception of Nelson NA, et al 2. 1994 control population.
(TIF)

Figure S7 **Forest plot of supplementary meta-analyses.** Final common effect size based on a random model. The studies included and abbreviations are the same as in Figure S1 with the exception of Purdie GL, et al 2. 2011 including confirmed and possible Raynaud.
(TIF)

Figure S8 **Forest plot of supplementary meta-analyses.** Final common effect size based on a random model. The studies included and abbreviations are the same as in Figure S1 with the exception of Thompson AE, et al. 2002 10. Exposition to Bicromade.
(TIF)

Figure S9 **Forest plot of supplementary meta-analyses.** Final common effect size based on a random model. The studies included and abbreviations are the same as in Figure S1 with the exception of Thompson AE, et al. 2002 11. Exposition to Toluene.
(TIF)

Figure S10 **Forest plot of supplementary meta-analyses.** Final common effect size based on a random model. The studies included and abbreviations are the same as in Figure S1 with the exception of Thompson AE, et al. 2002 12. Exposition to Aromatic hydrocarbons.
(TIF)

Figure S11 **Forest plot of supplementary meta-analyses.** Final common effect size based on a random model. The studies included and abbreviations are the same as in Figure S1 with the exception of Thompson AE, et al. 2002 13. Exposition to Aliphatic hydrocarbons.
(TIF)

Figure S12 **Forest plot of supplementary meta-analyses.** Final common effect size based on a random model. The studies included and abbreviations are the same as in Figure S1 with the exception of Thompson AE, et al. 2002 14. Exposition to Fenfluramine.
(TIF)

Figure S13 **Forest plot of supplementary meta-analyses.** Final common effect size based on a random model. The studies included and abbreviations are the same as in Figure S1 with the exception of Thompson AE, et al. 2002 15. Exposition to Diethylpropion.
(TIF)

Figure S14 **Forest plot of supplementary meta-analyses.** Final common effect size based on a random model. The studies included and abbreviations are the same as in Figure S1 with the exception of Thompson AE, et al. 2002 16. Exposition to L5 Ohtryptophan.
(TIF)

Figure S15 **Forest plot of supplementary meta-analyses.** Final common effect size based on a random model. The studies included and abbreviations are the same as in Figure S1 with the exception of Thompson AE, et al. 2002 2. Exposition to Benzene.
(TIF)

Figure S16 **Forest plot of supplementary meta-analyses.** Final common effect size based on a random model. The studies included and abbreviations are the same as in Figure S1 with the exception of Thompson AE, et al. 2002 3. Exposition to White spirit.
(TIF)

Figure S17 **Forest plot of supplementary meta-analyses.** Final common effect size based on a random model. The studies included and abbreviations are the same as in Figure S1 with the exception of Thompson AE, et al. 2002 4. Exposition to Perchlorethylene.
(TIF)

Figure S18 **Forest plot of supplementary meta-analyses.** Final common effect size based on a random model. The studies included and abbreviations are the same as in Figure S1 with the exception of Thompson AE, et al. 2002 5. Exposition to Trichlorethylene.
(TIF)

Figure S19 **Forest plot of supplementary meta-analyses.** Final common effect size based on a random model. The studies included and abbreviations are the same as in Figure S1 with the exception of Thompson AE, et al. 2002 6. Exposition to Trichlorethane.
(TIF)

Figure S20 **Forest plot of supplementary meta-analyses.** Final common effect size based on a random model. The studies included and abbreviations are the same as in Figure S1 with the exception of Thompson AE, et al. 2002 7. Exposition to vinyl chloride.
(TIF)

Figure S21 **Forest plot of supplementary meta-analyses.** Final common effect size based on a random model. The studies included and abbreviations are the same as in Figure S1 with the exception of Thompson AE, et al. 2002 8. Exposition to Urea formaldehyde.
(TIF)

Figure S22 **Forest plot of supplementary meta-analyses.** Final common effect size based on a random model. The studies included and abbreviations are the same as in Figure S1 with the exception of Thompson AE, et al. 2002 9. Exposition to Meta-phenylenediamene.
(TIF)

Figures S23 **Sensitivity analysis.** Footnote: Odds Ratio (95%CI) excluding one study at a time. CI: confidence interval. Diot, et al 1: organic solvent as a whole; Thompson AE, et al 1: turpentine exposure (the most significant result); Purdie GL, et al 1: confirmed RP population; Nelson NA, et al 1. 1994: disabled population.
(TIF)

Figures S24 **Cumulative analysis.** Footnote: Odds Ratio (95%CI) The most relevant outcome per author was included. CI: confidence interval. Diot, et al 1: organic solvent as a whole; Thompson AE, et al 1: turpentine exposure (the most significant result); Purdie GL, et al 1: confirmed RP population; Nelson NA, et al 1. 1994: disabled population.
(TIF)

Figure S25 **Forest plot of studies showing RR data and raw data from cohort studies.** Footnote: final common effect size based on a random model. Risk Ratio (95%CI). CI: confidence interval; AS: Ankylosing spondylitis; GN: glomerulonephritis; MS: multiple sclerosis; RA: rheumatoid arthritis; RP: Raynaud disease; SLE: systemic lupus erythematosus; SSc: systemic sclerosis. Lundberg I, et al. 1 1994 painters AS; Lundberg I, et al. 3 1994 Substantial RA men; Lundberg I, et al. 5 1994 substantial RA women; Purdie GL, et al 1: confirmed RP population.
(TIF)

Figure S26 **Forest plot of studies showing OR data according to the exposure assessment category.** Footnote: Odds Ratio (95%CI). The most relevant outcome per author was included. GN: glomerulonephritis; MS: multiple sclerosis; PBC: primary biliary cirrhosis; PSV: primary systemic vasculitis; RA: rheumatoid arthritis; RP: Raynaud disease; SLE: systemic lupus erythematosus; SSc: systemic sclerosis. Diot, et al 1: organic solvent as a whole; Thompson AE, et al 1: turpentine exposure (the most significant result); Purdie GL, et al 1: confirmed RP population; Nelson NA, et al 1. 1994: disabled population.
(TIF)

Figure S27 **Funnel Plot of standard error by log odds ratio.** Footnote: X-axis: Log odds ratio. Y-axis: Standard Error.
(TIF)

Figure S28 **Systematic Review Results for OSs molecular mechanisms related to responses of immune system and ADs.** Footnote: ADs: Autoimmune Diseases.
(TIF)

Text S1 **PRISMA 2009 Checklist.** PRISMA: Preferred Reporting Items for Systematic Reviews and Meta-Analyses.
(DOCX)

Table S1 Studies not included in the Meta-analysis. Footnote: AD: Autoimmune Disease; C-C: Case Control Study; OS: Organic Solvent; SSc: Systemic Sclerosis or Scleroderma; SLE: Systemic Lupus Erythematous; MS: Multiple Sclerosis; PSV: Primary systemic vasculitis; RA: Rheumatoid Arthritis; PBC: Primary Biliary Cirrhosis; GN: Glomerulonephritis; y/o: years old; VC: vinyl chloride; TCE: trichloroethylene; PCE: Perchlorethylene; RDX: Royal Demolition explosive; EEG: electroencephalographic study; PVC: polyvinyl chloride; ESRD: End Stage Renal Disease.
(DOCX)

Table S2 Case reports and case series. Footnote: AD: Autoimmune Disease; OS: Organic Solvent; SSc: Systemic Sclerosis or Scleroderma; SLE: Systemic Lupus Erythematous; MS: Multiple Sclerosis; PSV: Primary systemic vasculitis; RA: Rheumatoid Arthritis; RD: Raynaud Disease; PBC: Primary Biliary Cirrhosis; GN: Glomerulonephritis; Anti- GBM: Anti-glomerular basement membrane antibody; PM/DM: Polimiositis/Dermatomiositis; y/o: years old; VC: vinyl chloride; TCE: trichloroethylene; PCE: Perchlorethylene; Jo-1: anti-histidyl-t-RNA synthetase.
(DOCX)

Table S3 Search strategy related to solvent exposure and immune alterations.
(DOCX)

Table S4 Effects of the exposition to organic solvents on experimental models.
(DOCX)
