# Supplementary figures and images for: Organic Solvents as Risk Factor for Autoimmune Diseases: A Systematic Review and Meta-Analysis
Source: PLoS One. 2012 Dec 19;7(12):e51506. doi: 10.1371/journal.pone.0051506 (PMC3526640; doi:10.1371/journal.pone.0051506)

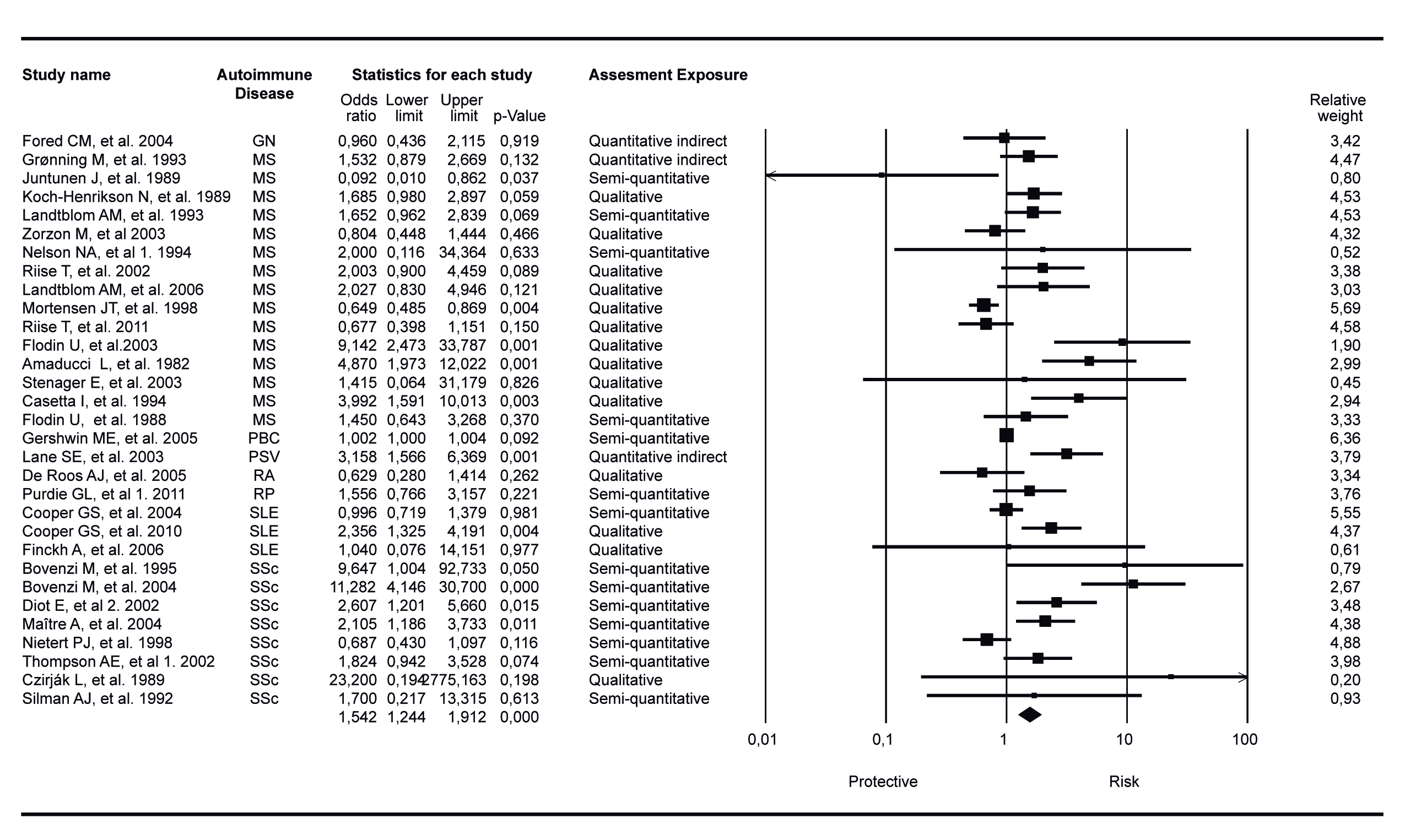

Supplement: Figure S1 — Forest plot of supplementary meta-analyses. Footnote: final common effect size based on a random model. Odds Ratio (95%CI) with raw data from case control and cohort designed studies were included. Studies that provided uniquely RR data were not included for statistical reasons. Each different outcome of the studies with complex data structure was included.GN: glomerulonephritis; MS: multiple sclerosis; PBC: primary biliary cirrhosis; PSV: primary systemic vasculitis; RA: rheumatoid arthritis; RP: Raynaud disease; SLE: systemic lupus erythematosus; SSc: systemic sclerosis. The complex data structure and non-cumulative results of articles showing multiple independent or dependent subgroups included in the analysis was S1 Diot E, et al.2 2002 Exposition to chlorinate. (TIF) [file pone.0051506.s001.tif]

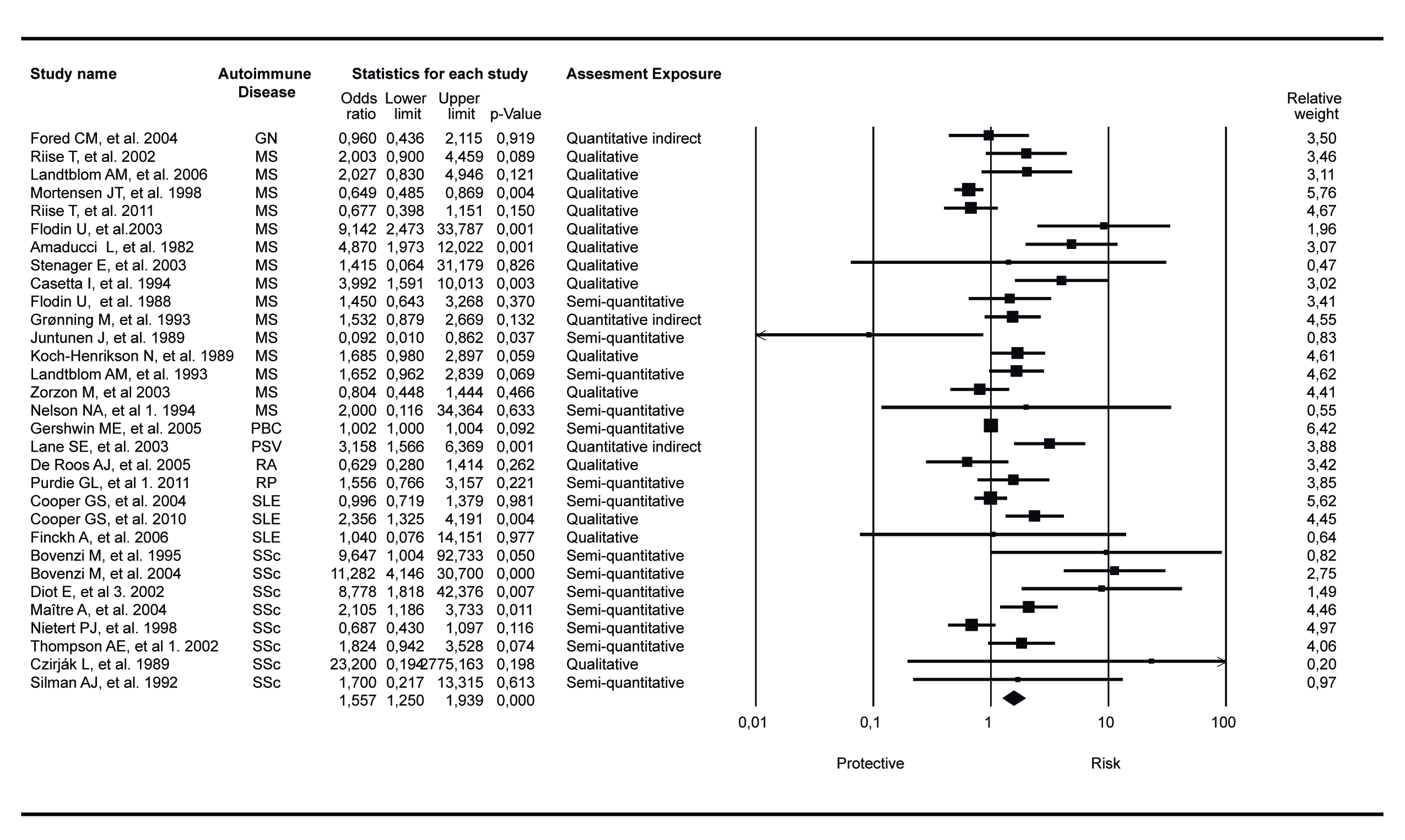

Supplement: Figure S2 — Forest plot of supplementary meta-analyses. Final common effect size based on a random model. The studies included and abbreviations are the same as in Figure S1 with the exception of Diot E, et al.3 2002. Exposition to ketones. (TIF) [file pone.0051506.s002.tif]

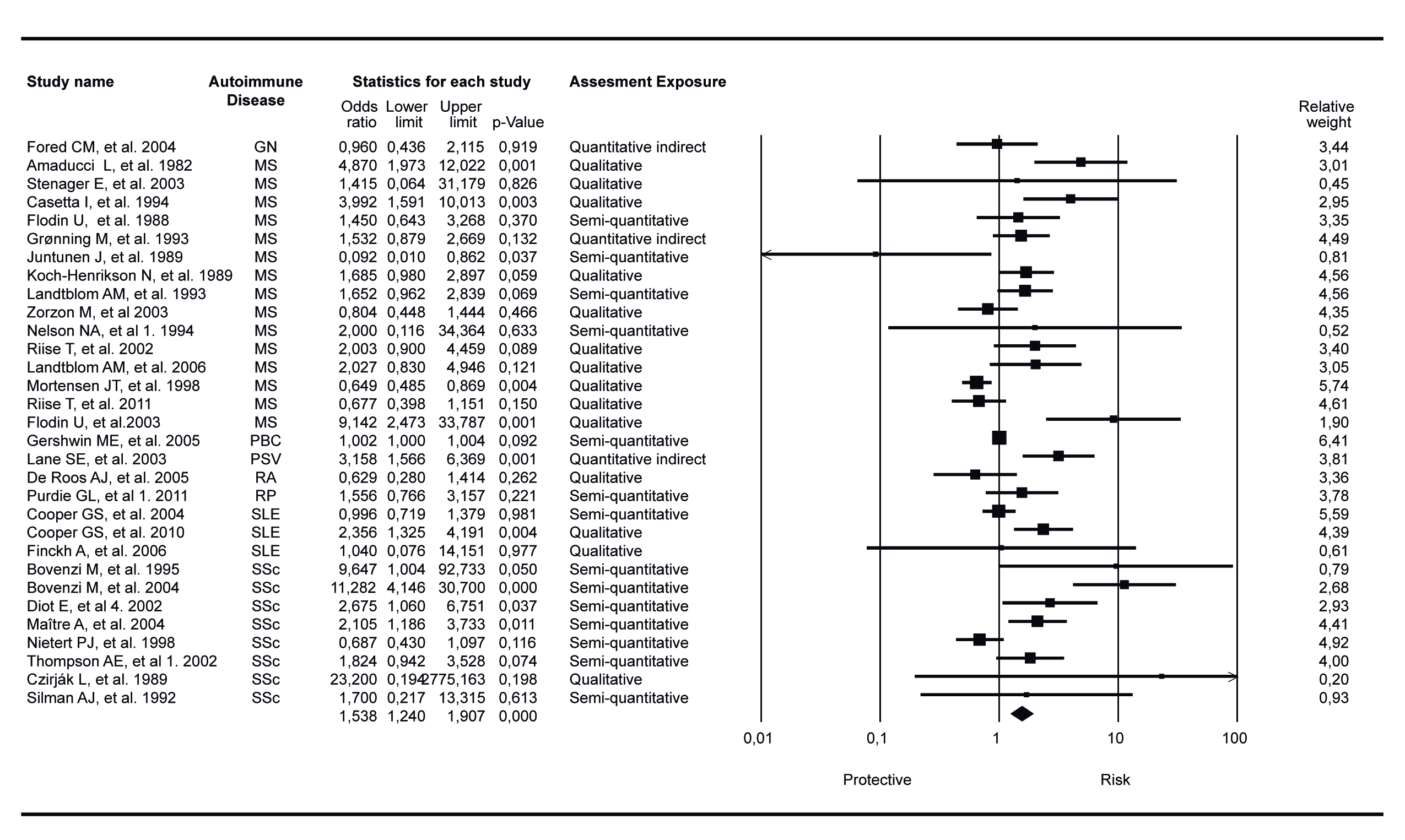

Supplement: Figure S3 — Forest plot of supplementary meta-analyses. Final common effect size based on a random model. The studies included and abbreviations are the same as in Figure S1 with the exception of Diot E, et al.4 2002. Exposition to aromatic. (TIF) [file pone.0051506.s003.tif]

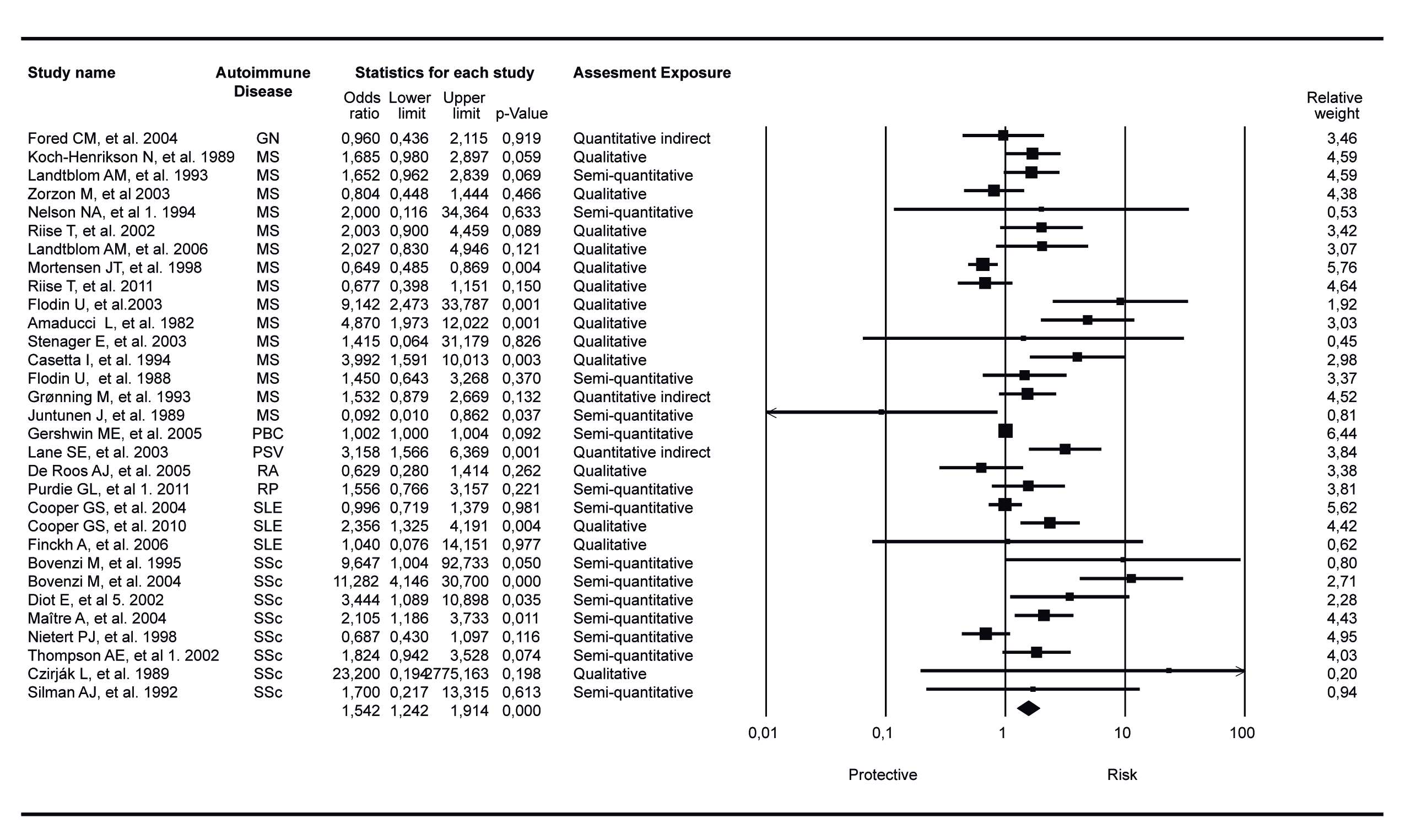

Supplement: Figure S4 — Forest plot of supplementary meta-analyses. Final common effect size based on a random model. The studies included and abbreviations are the same as in Figure S1 with the exception of Diot E, et al.5 2002. Exposition to toluene. (TIF) [file pone.0051506.s004.tif]

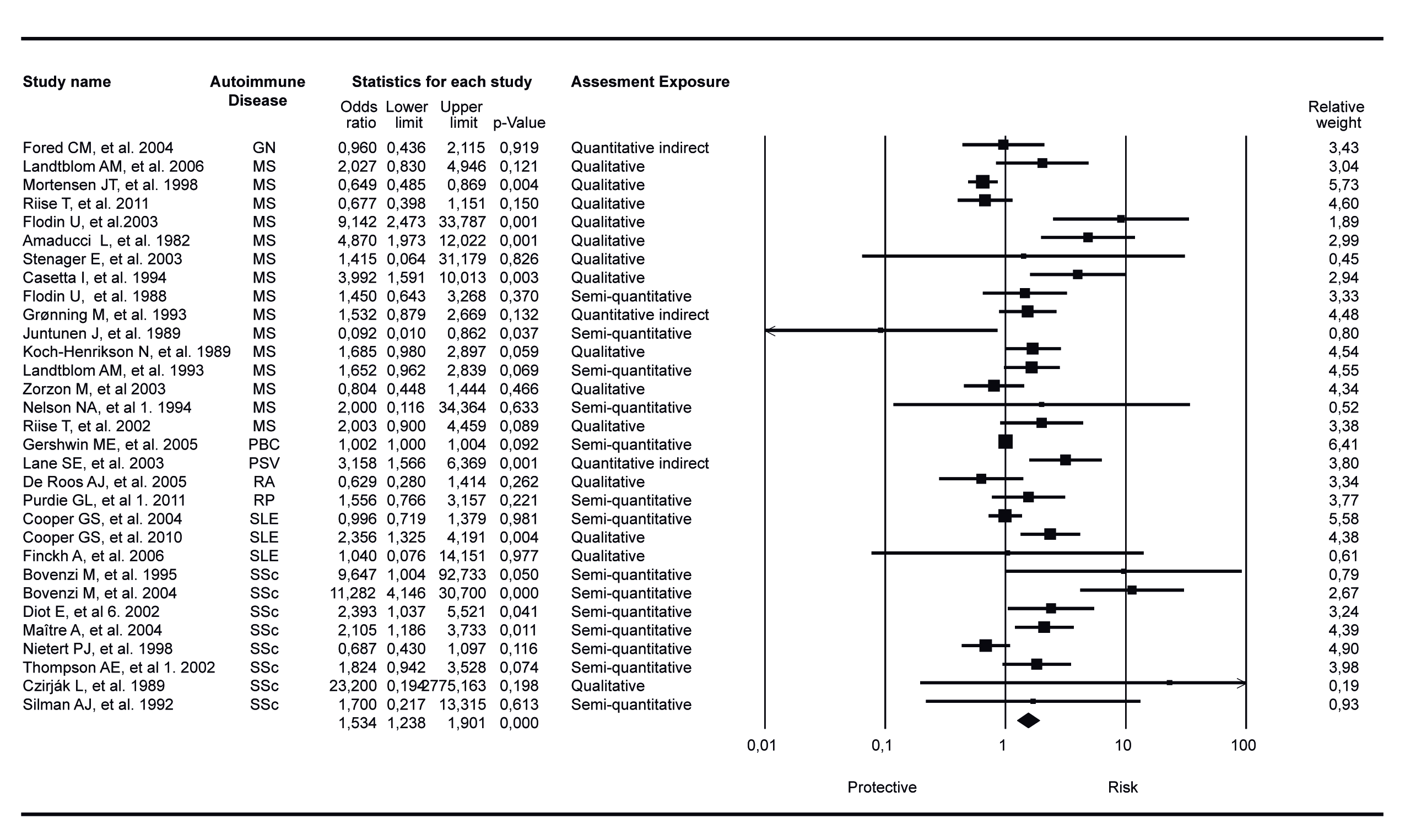

Supplement: Figure S5 — Forest plot of supplementary meta-analyses. Final common effect size based on a random model. The studies included and abbreviations are the same as in Figure S1 with the exception of Diot e, et al.6. Exposition to TCE. (TIF) [file pone.0051506.s005.tif]

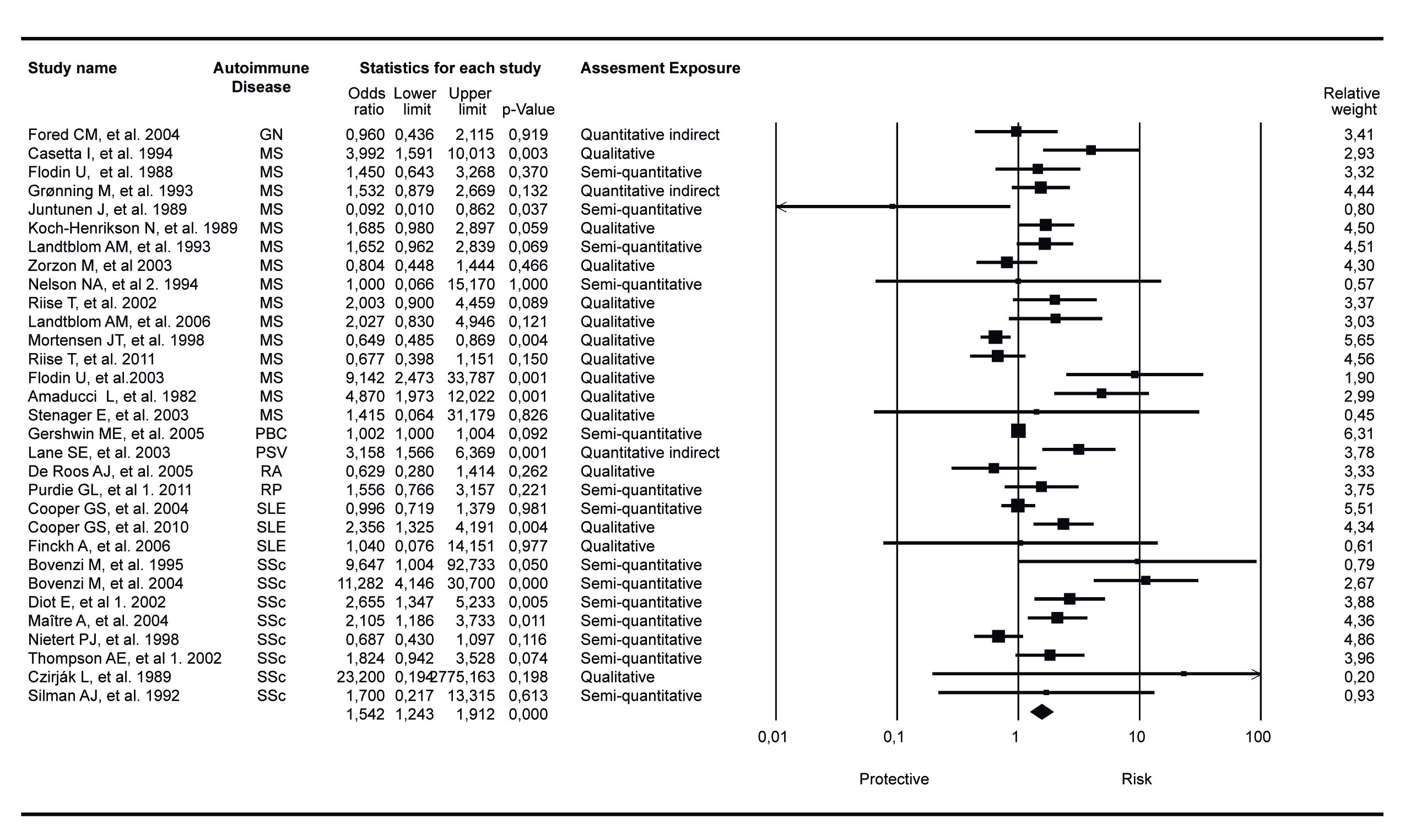

Supplement: Figure S6 — Forest plot of supplementary meta-analyses. Final common effect size based on a random model. The studies included and abbreviations are the same as in Figure S1 with the exception of Nelson NA, et al 2. 1994 control population. (TIF) [file pone.0051506.s006.tif]

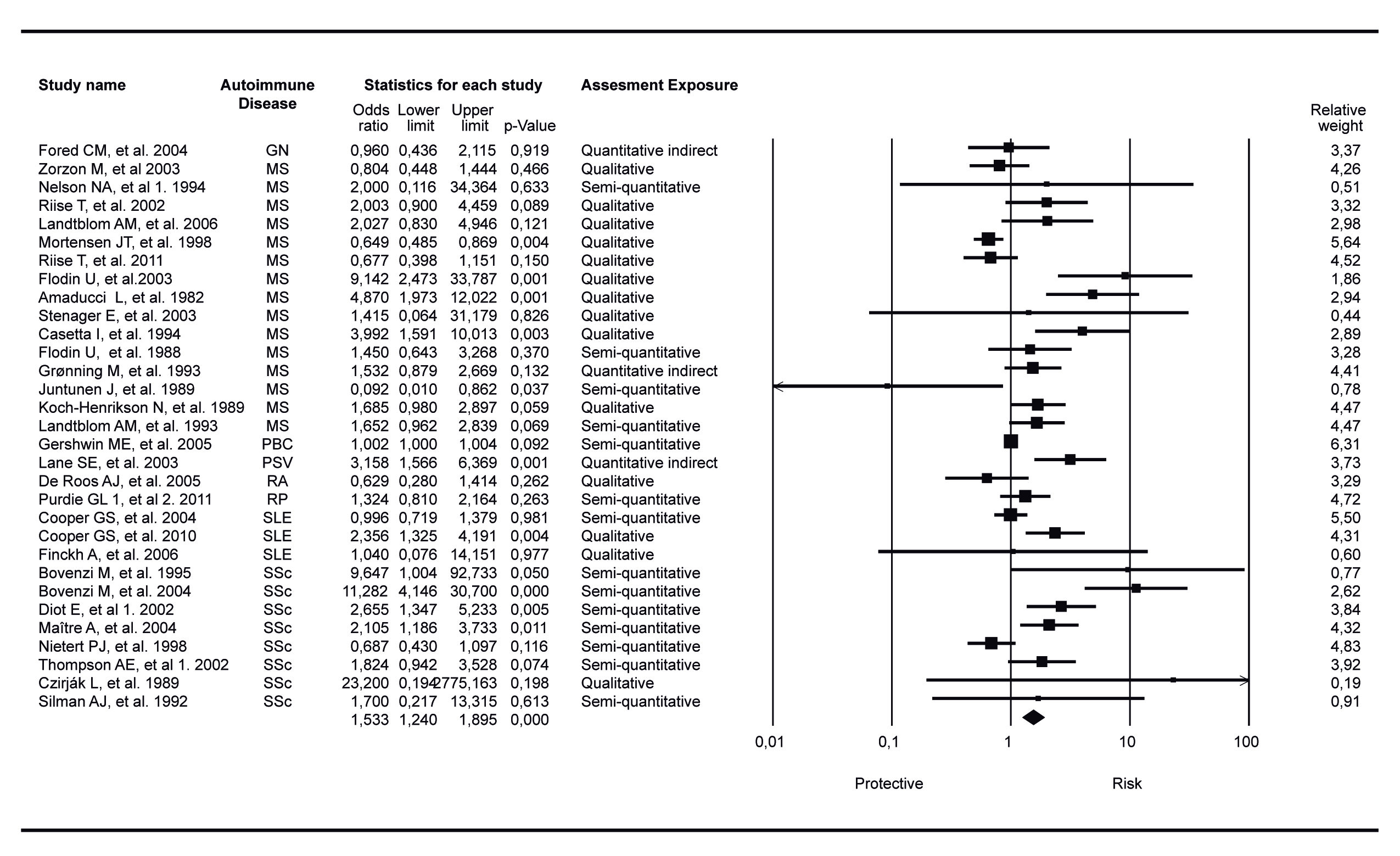

Supplement: Figure S7 — Forest plot of supplementary meta-analyses. Final common effect size based on a random model. The studies included and abbreviations are the same as in Figure S1 with the exception of Purdie GL, et al 2. 2011 including confirmed and possible Raynaud. (TIF) [file pone.0051506.s007.tif]

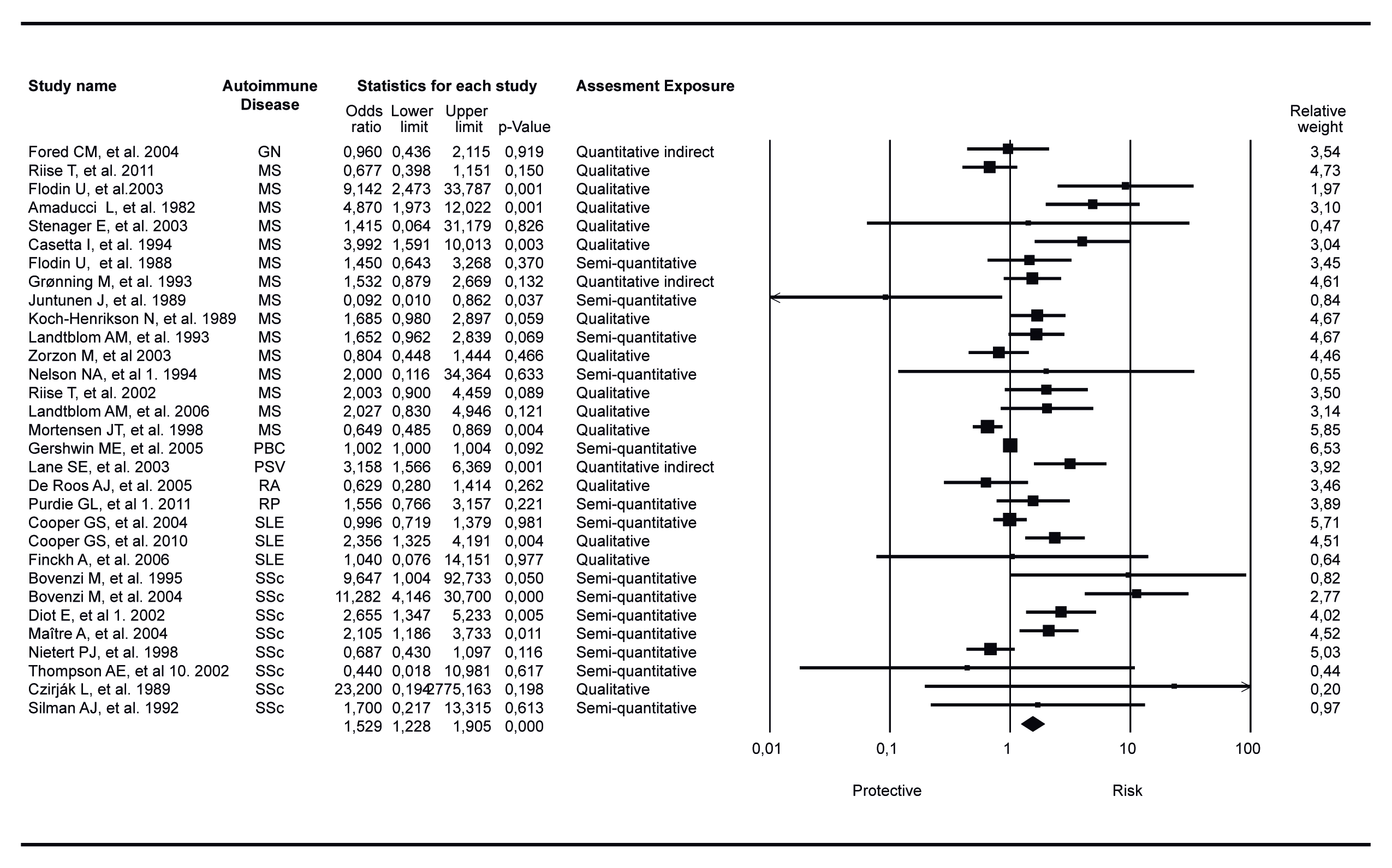

Supplement: Figure S8 — Forest plot of supplementary meta-analyses. Final common effect size based on a random model. The studies included and abbreviations are the same as in Figure S1 with the exception of Thompson AE, et al. 2002 10. Exposition to Bicromade. (TIF) [file pone.0051506.s008.tif]

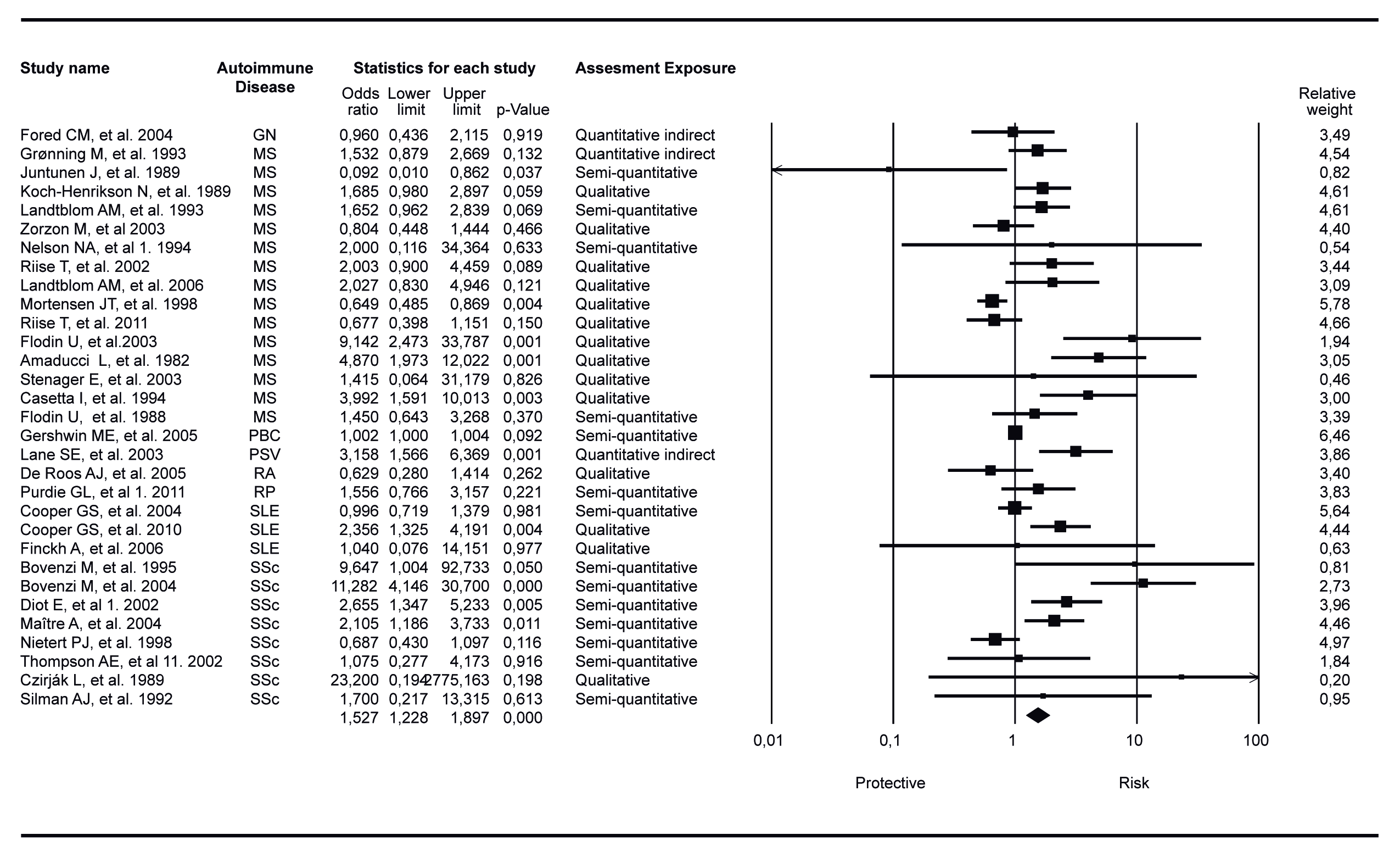

Supplement: Figure S9 — Forest plot of supplementary meta-analyses. Final common effect size based on a random model. The studies included and abbreviations are the same as in Figure S1 with the exception of Thompson AE, et al. 2002 11. Exposition to Toluene. (TIF) [file pone.0051506.s009.tif]

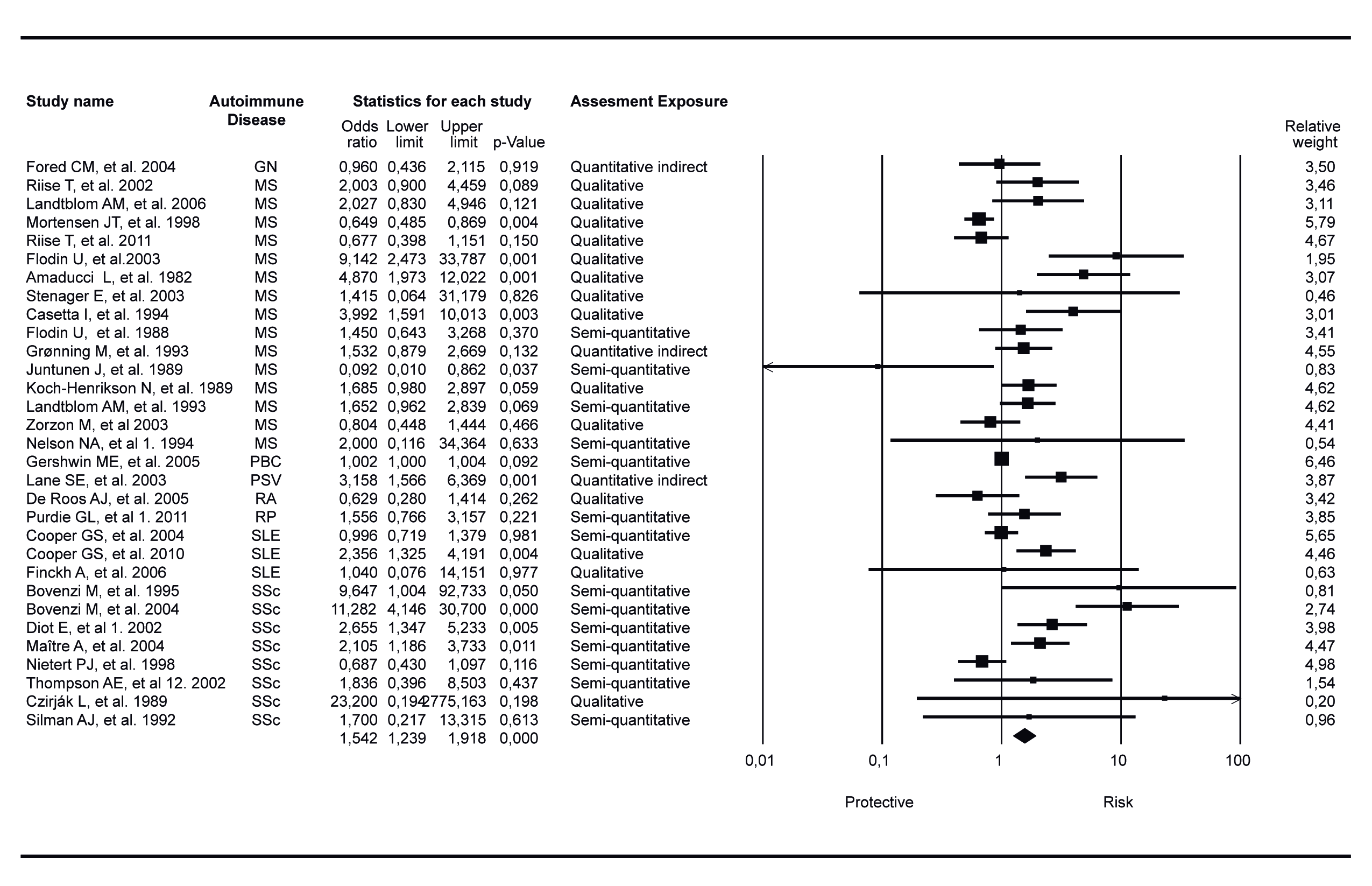

Supplement: Figure S10 — Forest plot of supplementary meta-analyses. Final common effect size based on a random model. The studies included and abbreviations are the same as in Figure S1 with the exception of Thompson AE, et al. 2002 12. Exposition to Aromatic hydrocarbons. (TIF) [file pone.0051506.s010.tif]

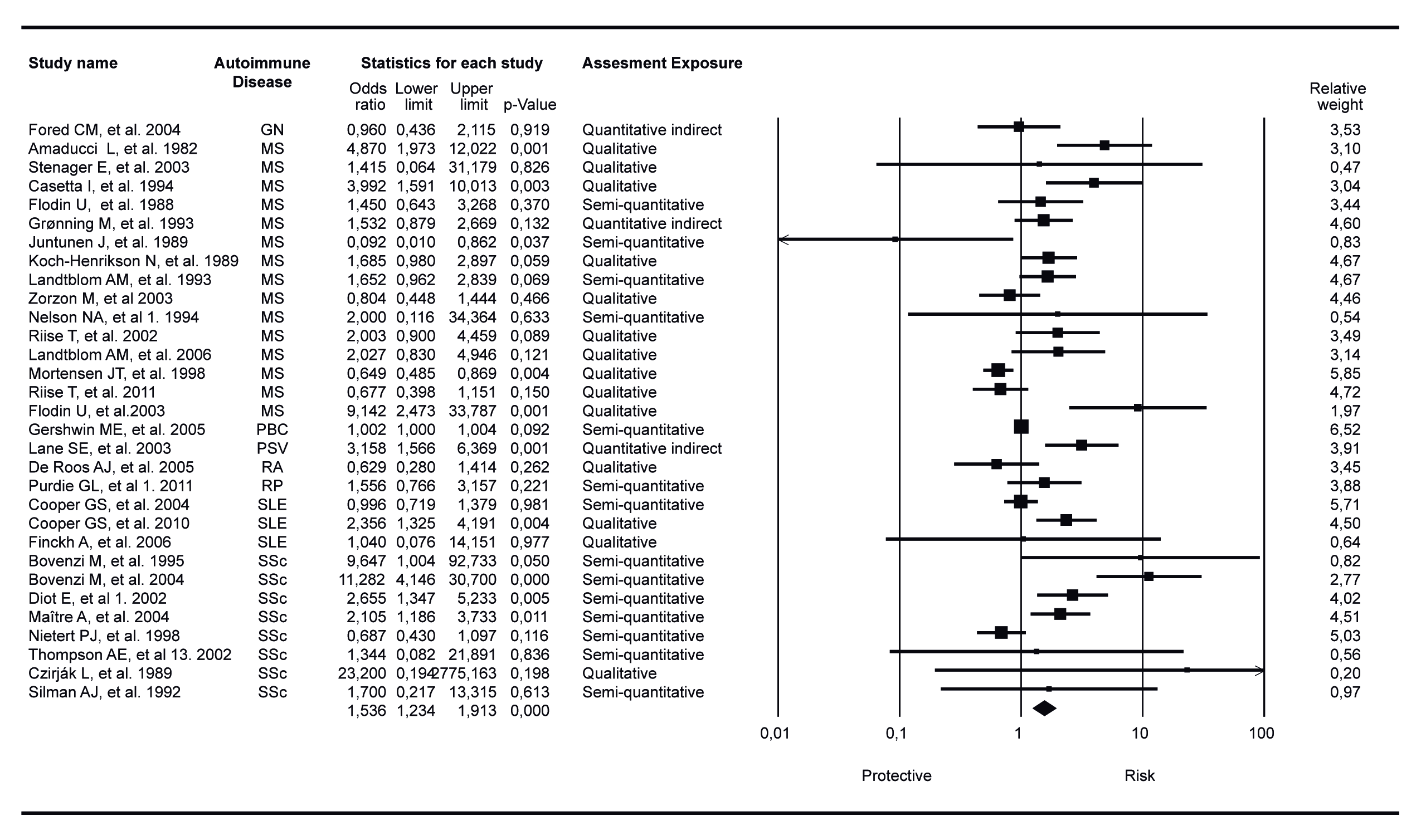

Supplement: Figure S11 — Forest plot of supplementary meta-analyses. Final common effect size based on a random model. The studies included and abbreviations are the same as in Figure S1 with the exception of Thompson AE, et al. 2002 13. Exposition to Aliphatic hydrocarbons. (TIF) [file pone.0051506.s011.tif]

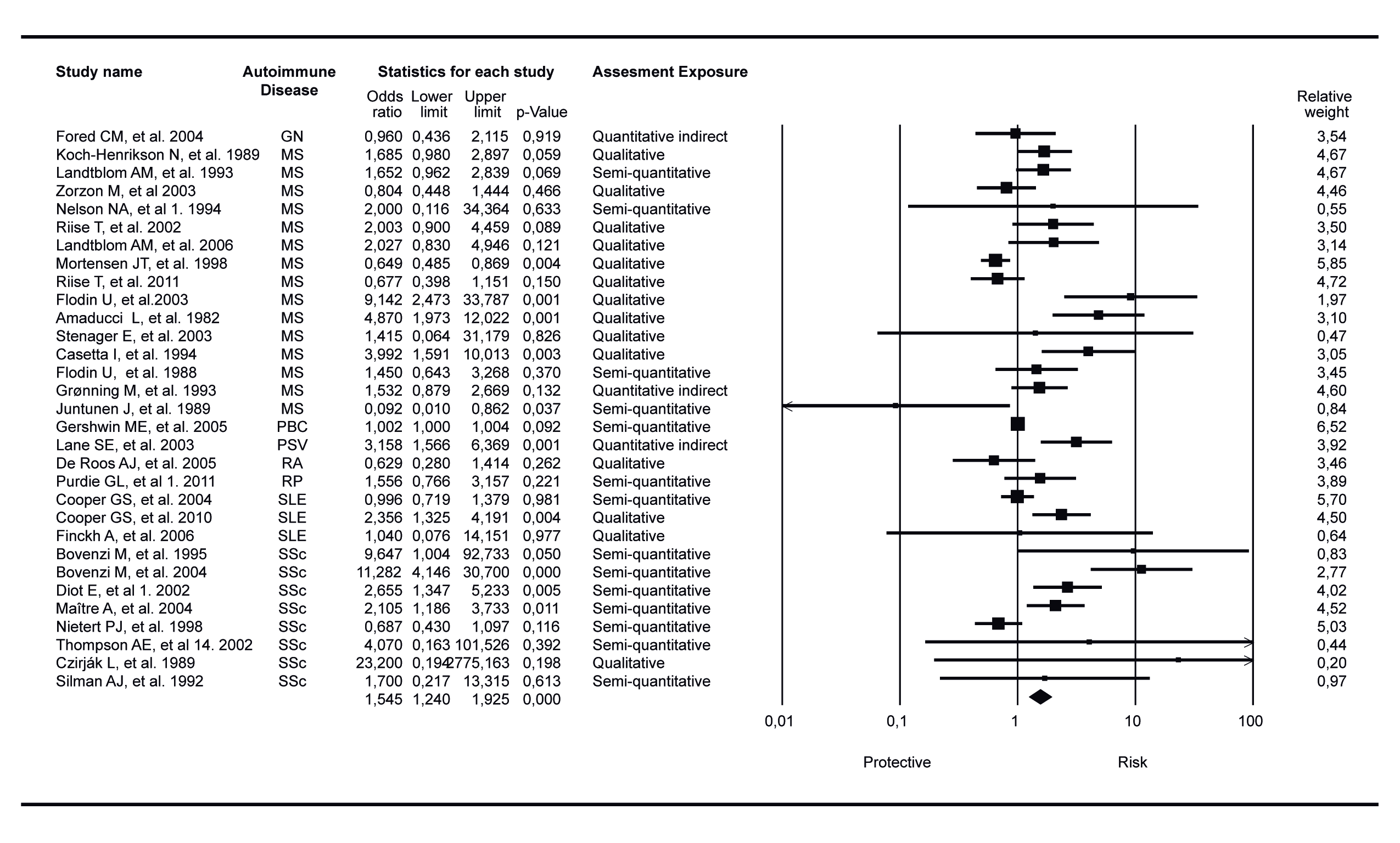

Supplement: Figure S12 — Forest plot of supplementary meta-analyses. Final common effect size based on a random model. The studies included and abbreviations are the same as in Figure S1 with the exception of Thompson AE, et al. 2002 14. Exposition to Fenfluramine. (TIF) [file pone.0051506.s012.tif]

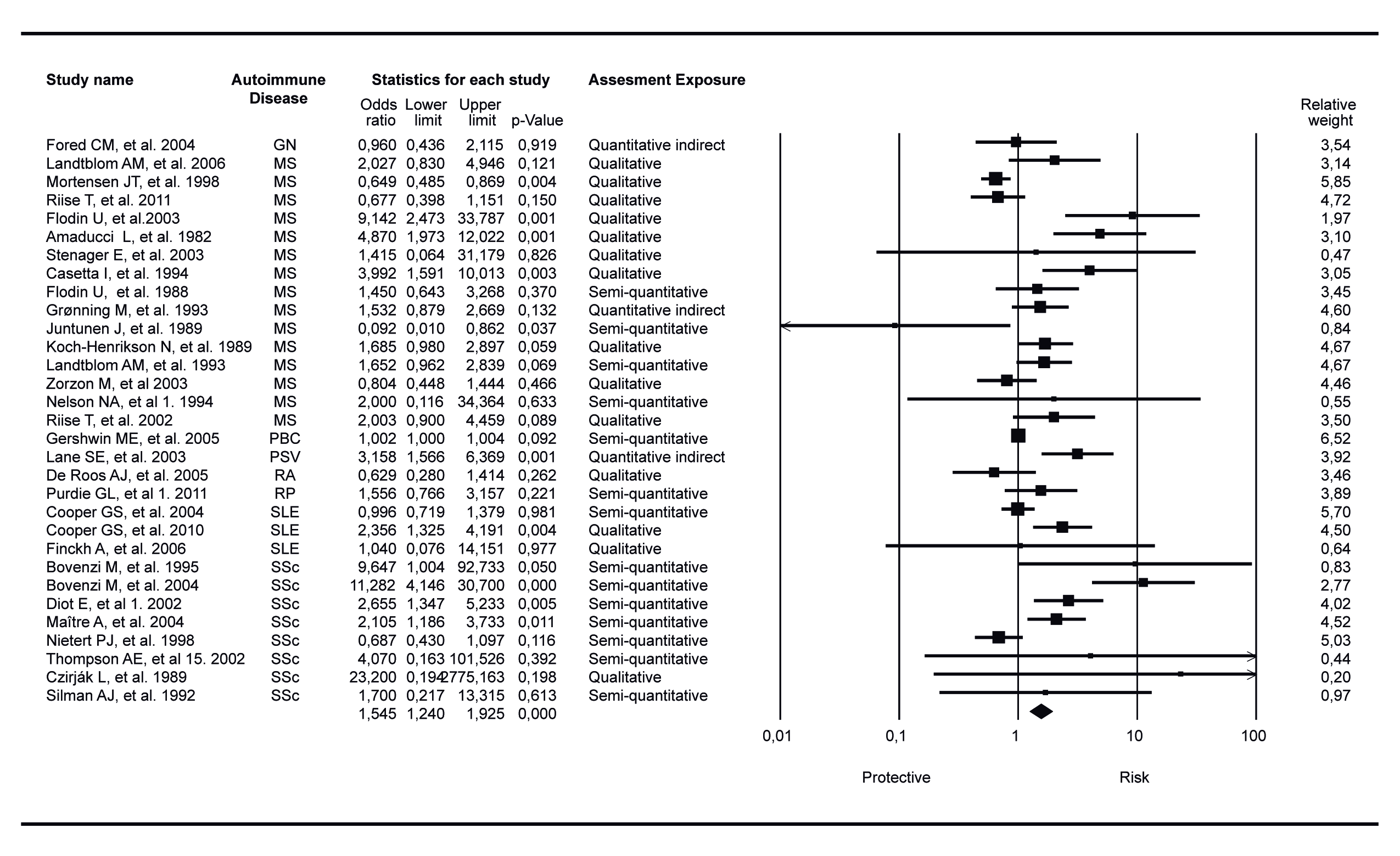

Supplement: Figure S13 — Forest plot of supplementary meta-analyses. Final common effect size based on a random model. The studies included and abbreviations are the same as in Figure S1 with the exception of Thompson AE, et al. 2002 15. Exposition to Diethylpropion. (TIF) [file pone.0051506.s013.tif]

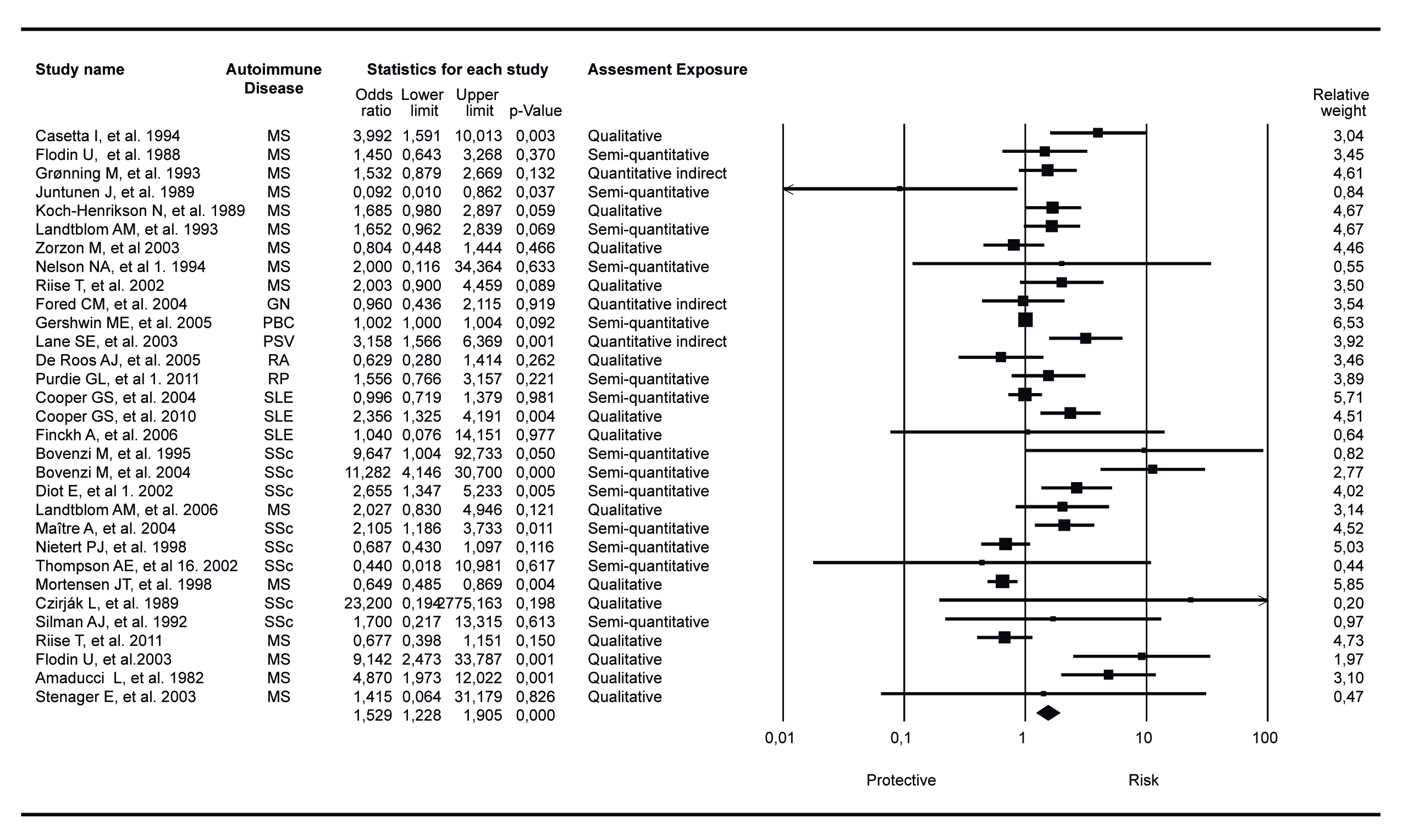

Supplement: Figure S14 — Forest plot of supplementary meta-analyses. Final common effect size based on a random model. The studies included and abbreviations are the same as in Figure S1 with the exception of Thompson AE, et al. 2002 16. Exposition to L5 Ohtryptophan. (TIF) [file pone.0051506.s014.tif]

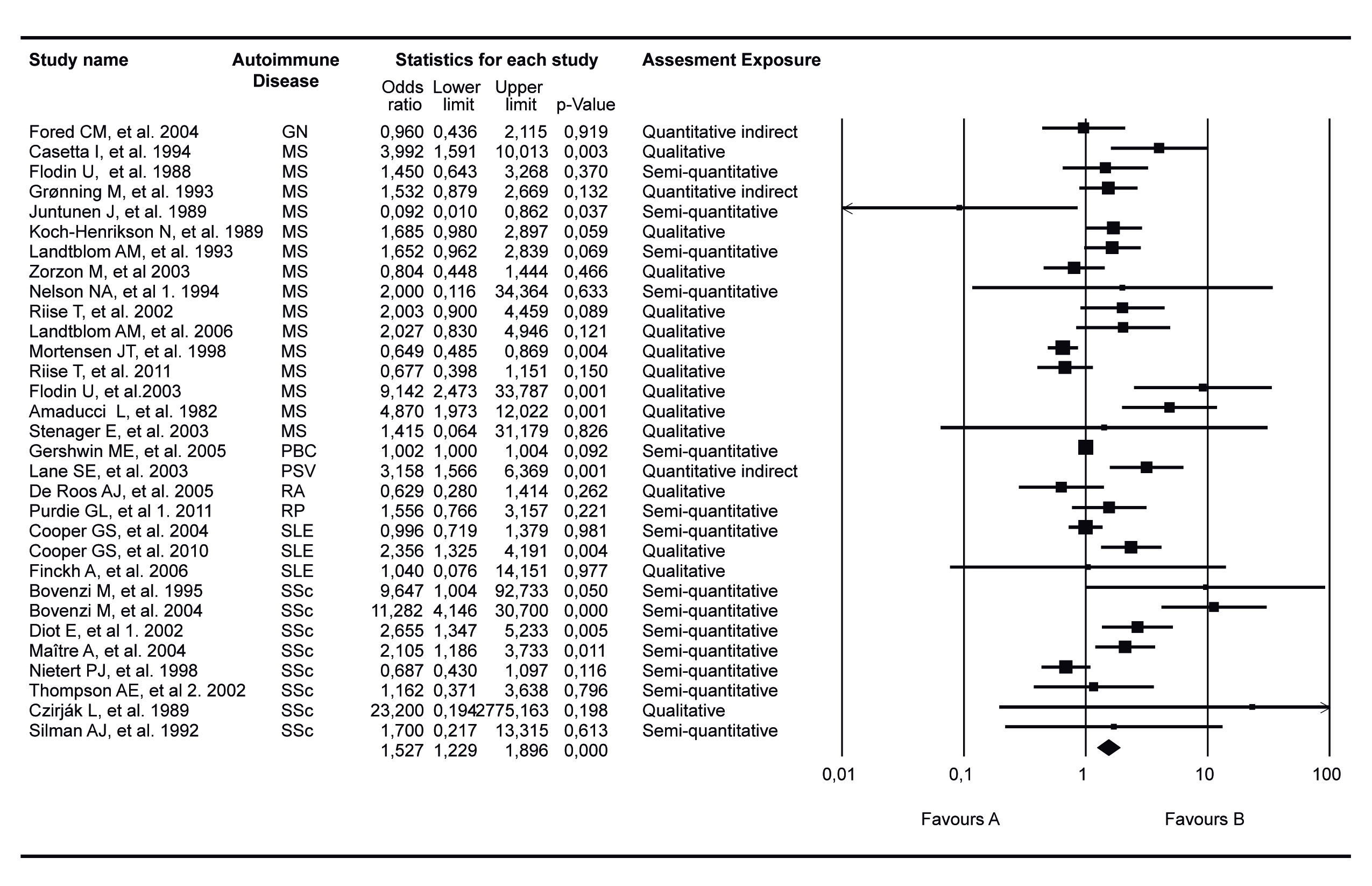

Supplement: Figure S15 — Forest plot of supplementary meta-analyses. Final common effect size based on a random model. The studies included and abbreviations are the same as in Figure S1 with the exception of Thompson AE, et al. 2002 2. Exposition to Benzene. (TIF) [file pone.0051506.s015.tif]

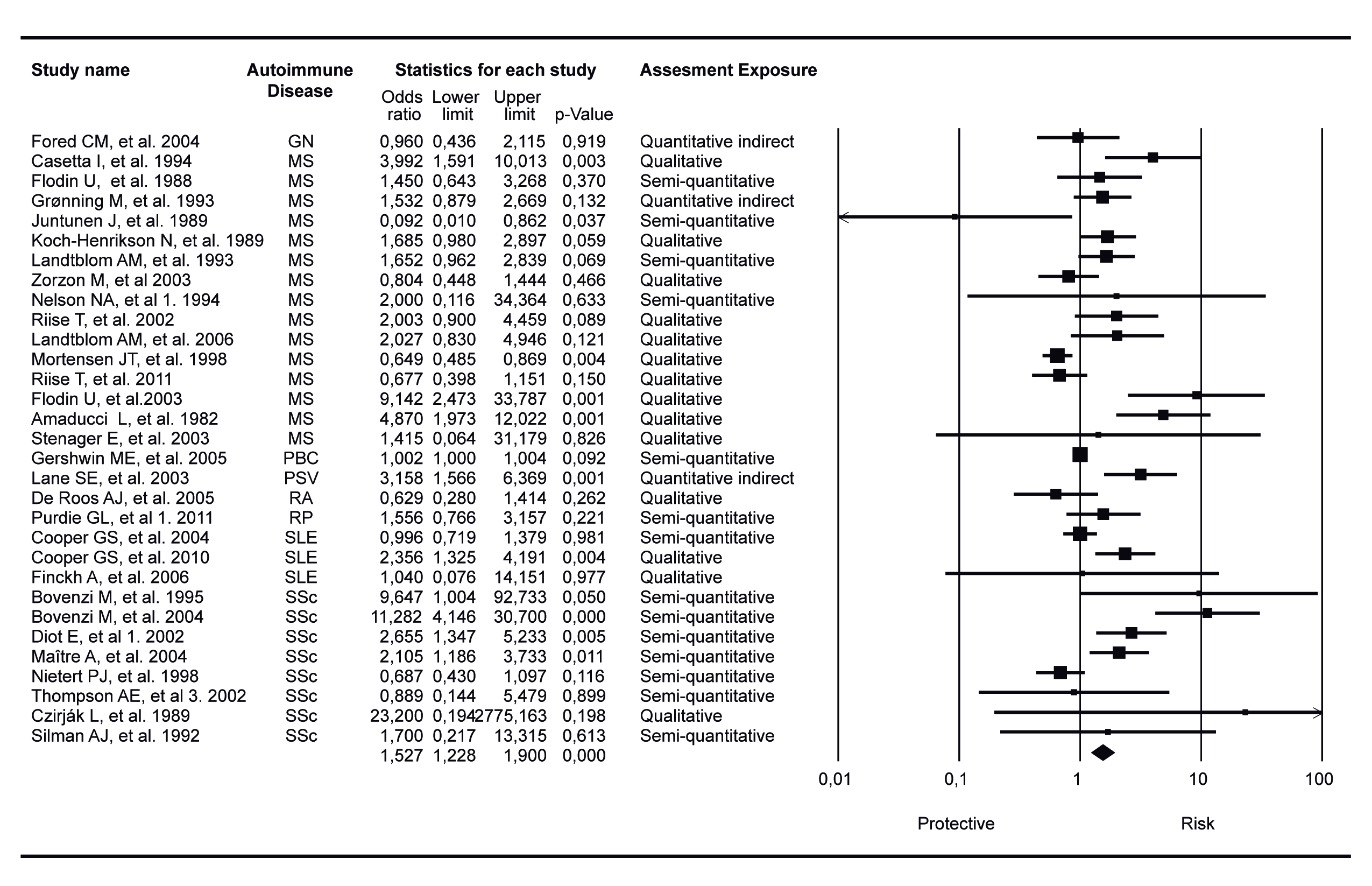

Supplement: Figure S16 — Forest plot of supplementary meta-analyses. Final common effect size based on a random model. The studies included and abbreviations are the same as in Figure S1 with the exception of Thompson AE, et al. 2002 3. Exposition to White spirit. (TIF) [file pone.0051506.s016.tif]

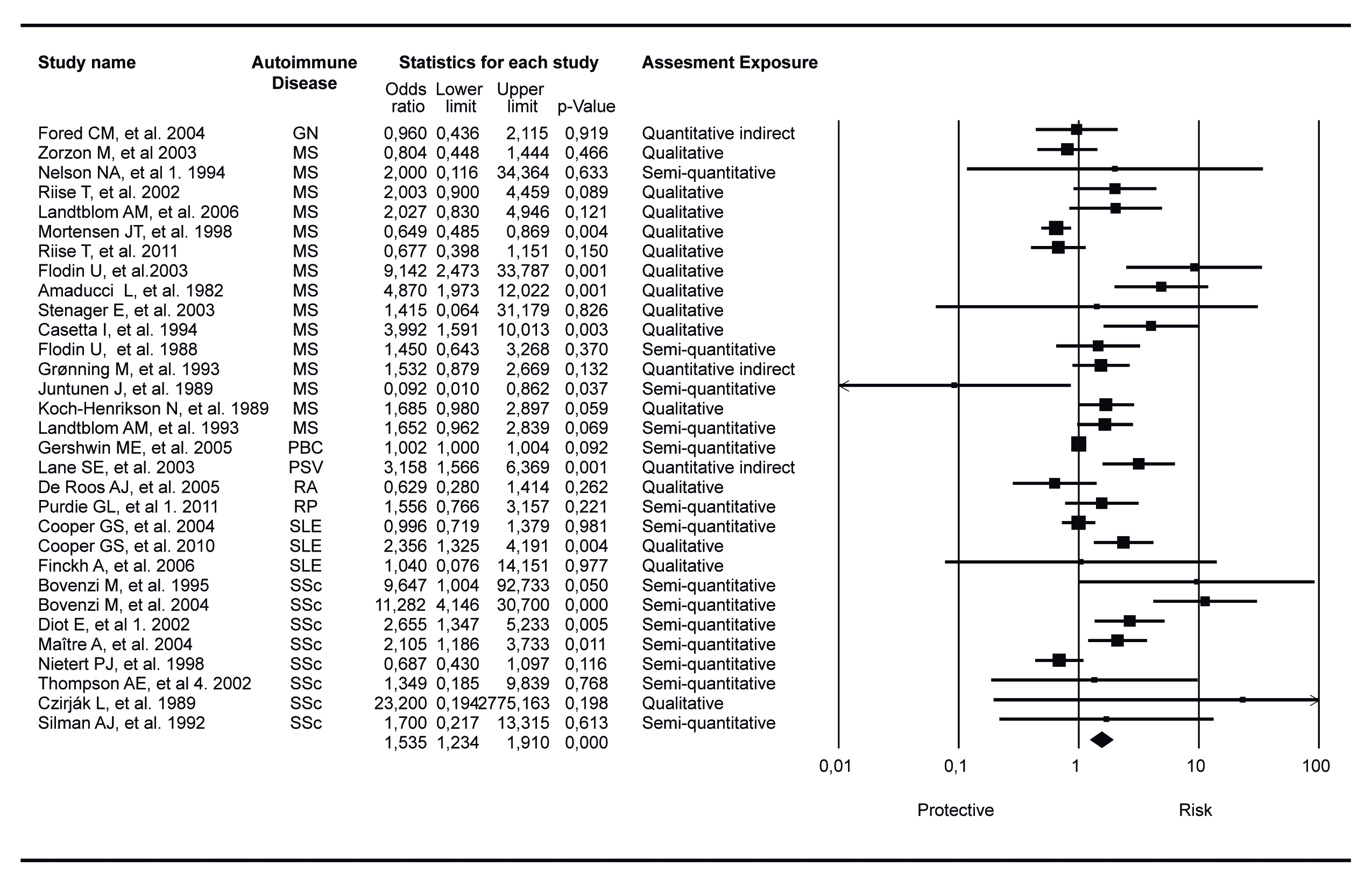

Supplement: Figure S17 — Forest plot of supplementary meta-analyses. Final common effect size based on a random model. The studies included and abbreviations are the same as in Figure S1 with the exception of Thompson AE, et al. 2002 4. Exposition to Perchlorethylene. (TIF) [file pone.0051506.s017.tif]

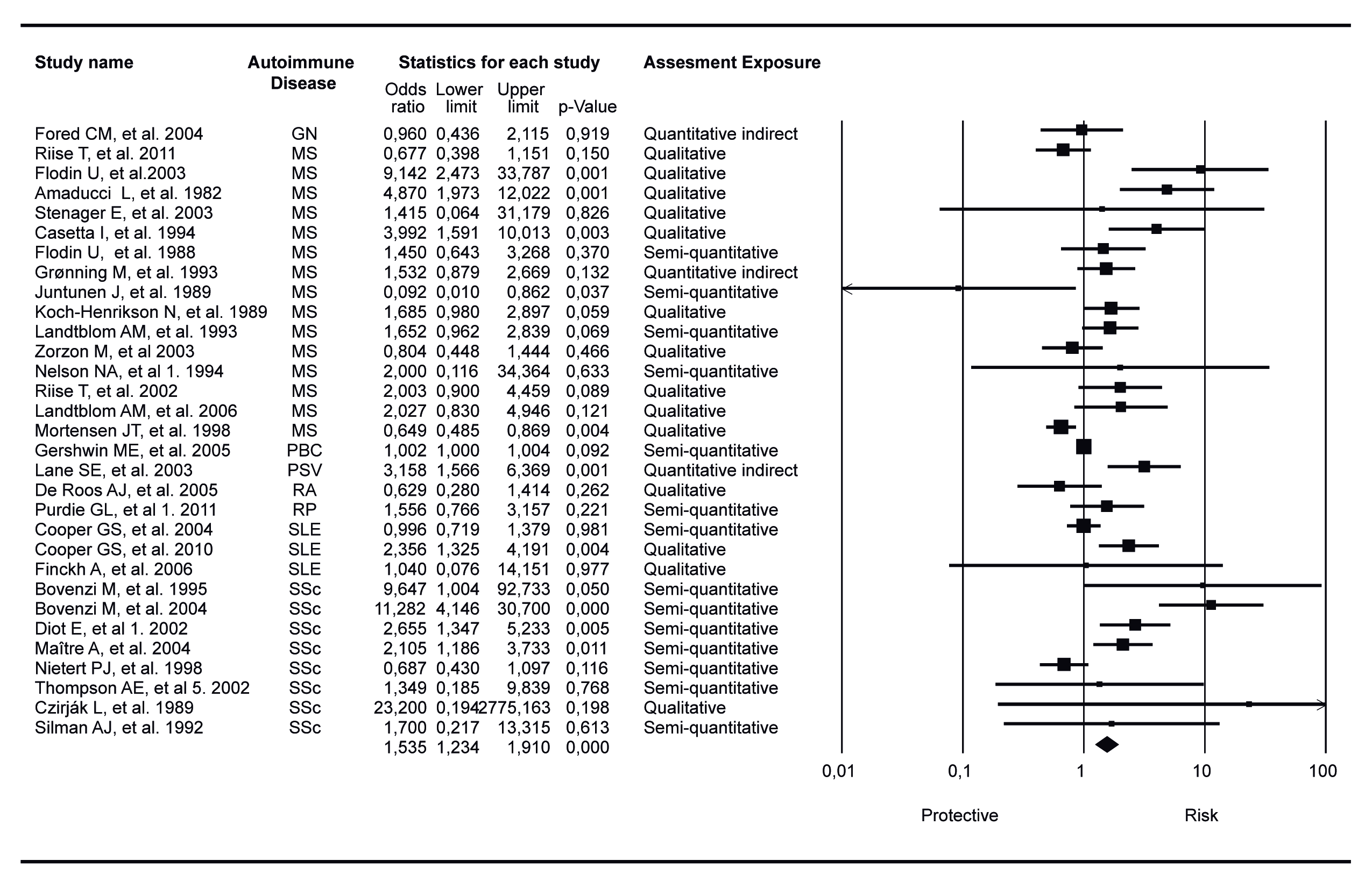

Supplement: Figure S18 — Forest plot of supplementary meta-analyses. Final common effect size based on a random model. The studies included and abbreviations are the same as in Figure S1 with the exception of Thompson AE, et al. 2002 5. Exposition to Trichlorethylene. (TIF) [file pone.0051506.s018.tif]

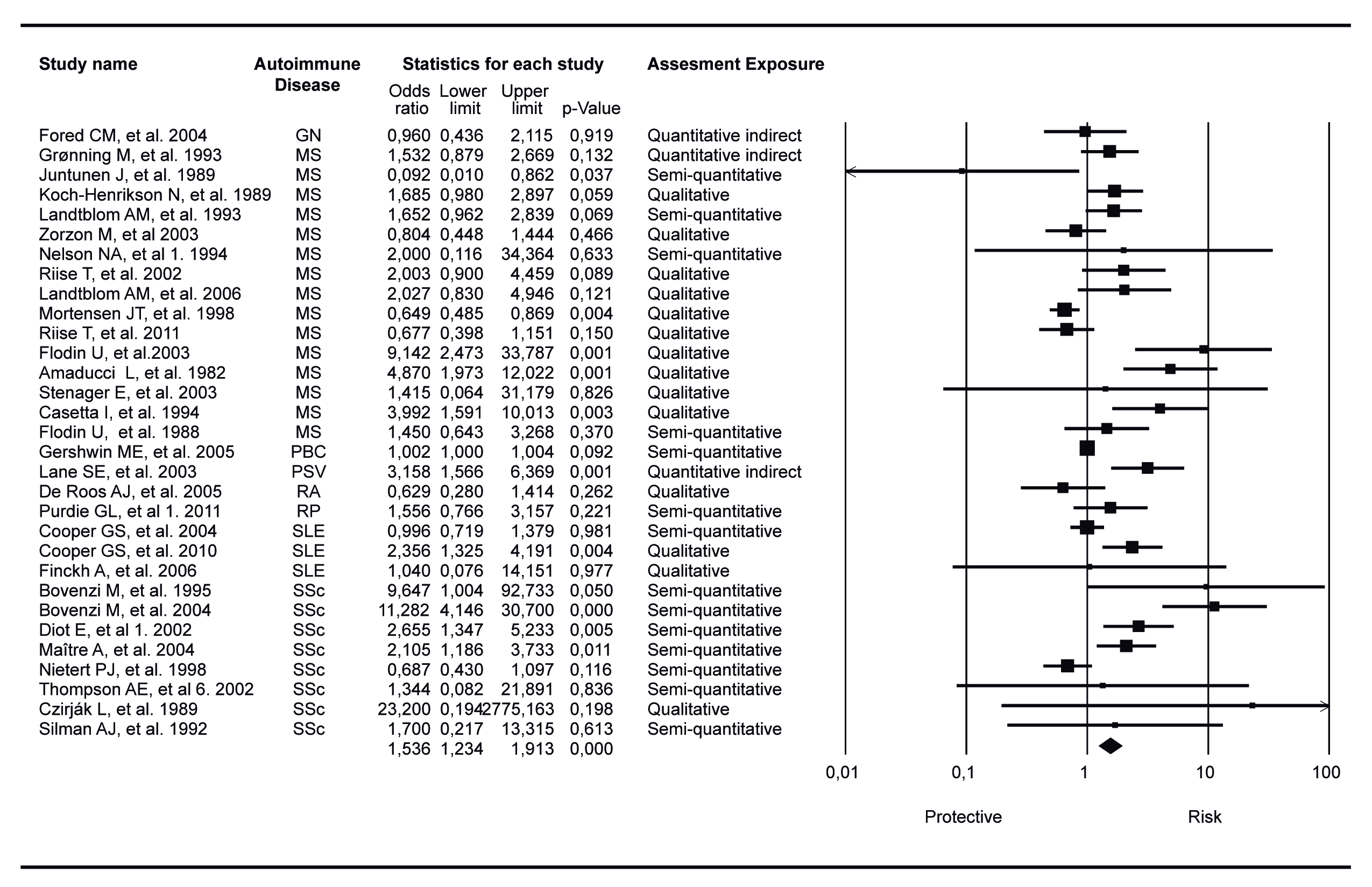

Supplement: Figure S19 — Forest plot of supplementary meta-analyses. Final common effect size based on a random model. The studies included and abbreviations are the same as in Figure S1 with the exception of Thompson AE, et al. 2002 6. Exposition to Trichlorethane. (TIF) [file pone.0051506.s019.tif]

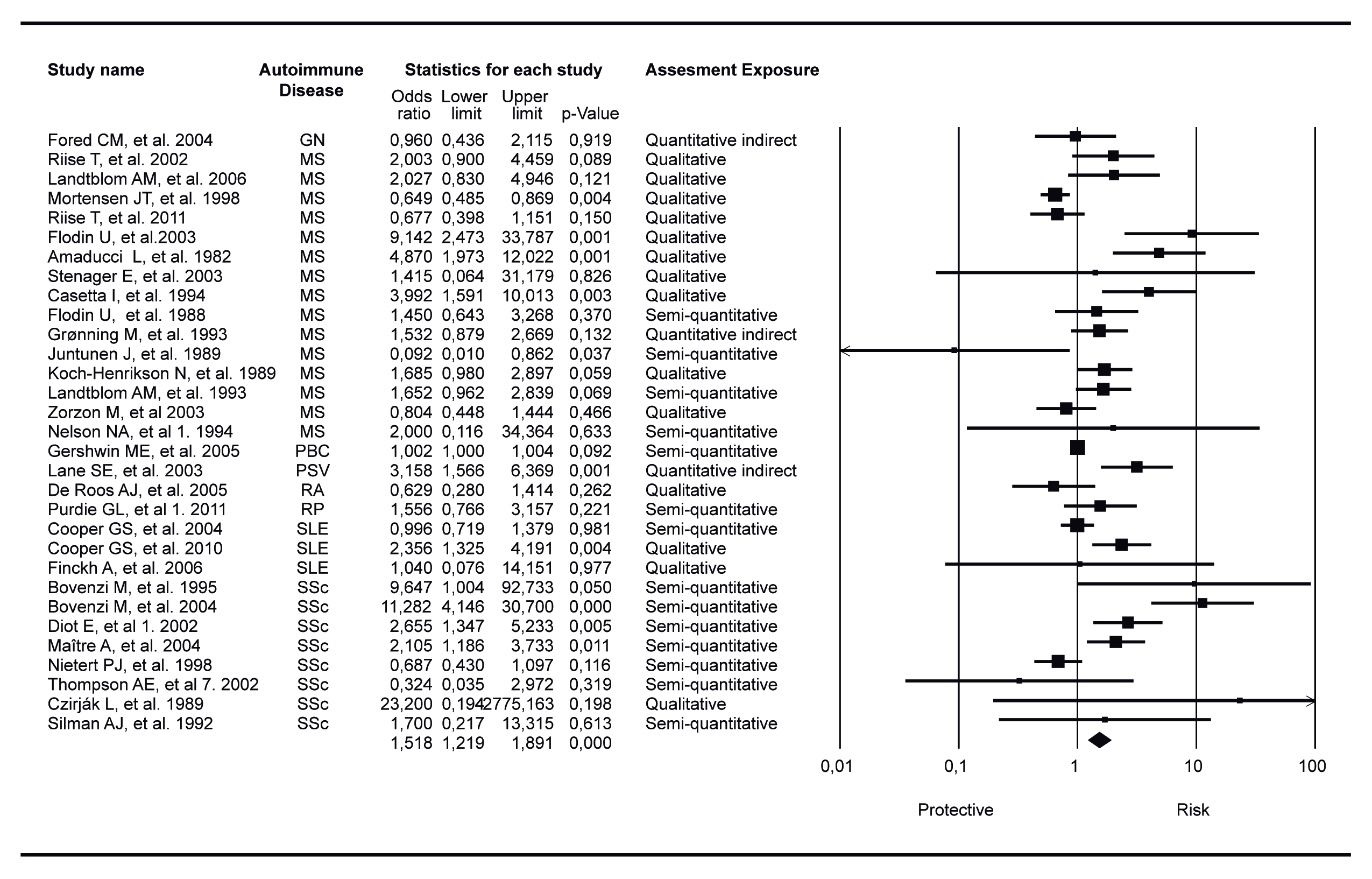

Supplement: Figure S20 — Forest plot of supplementary meta-analyses. Final common effect size based on a random model. The studies included and abbreviations are the same as in Figure S1 with the exception of Thompson AE, et al. 2002 7. Exposition to vinyl chloride. (TIF) [file pone.0051506.s020.tif]

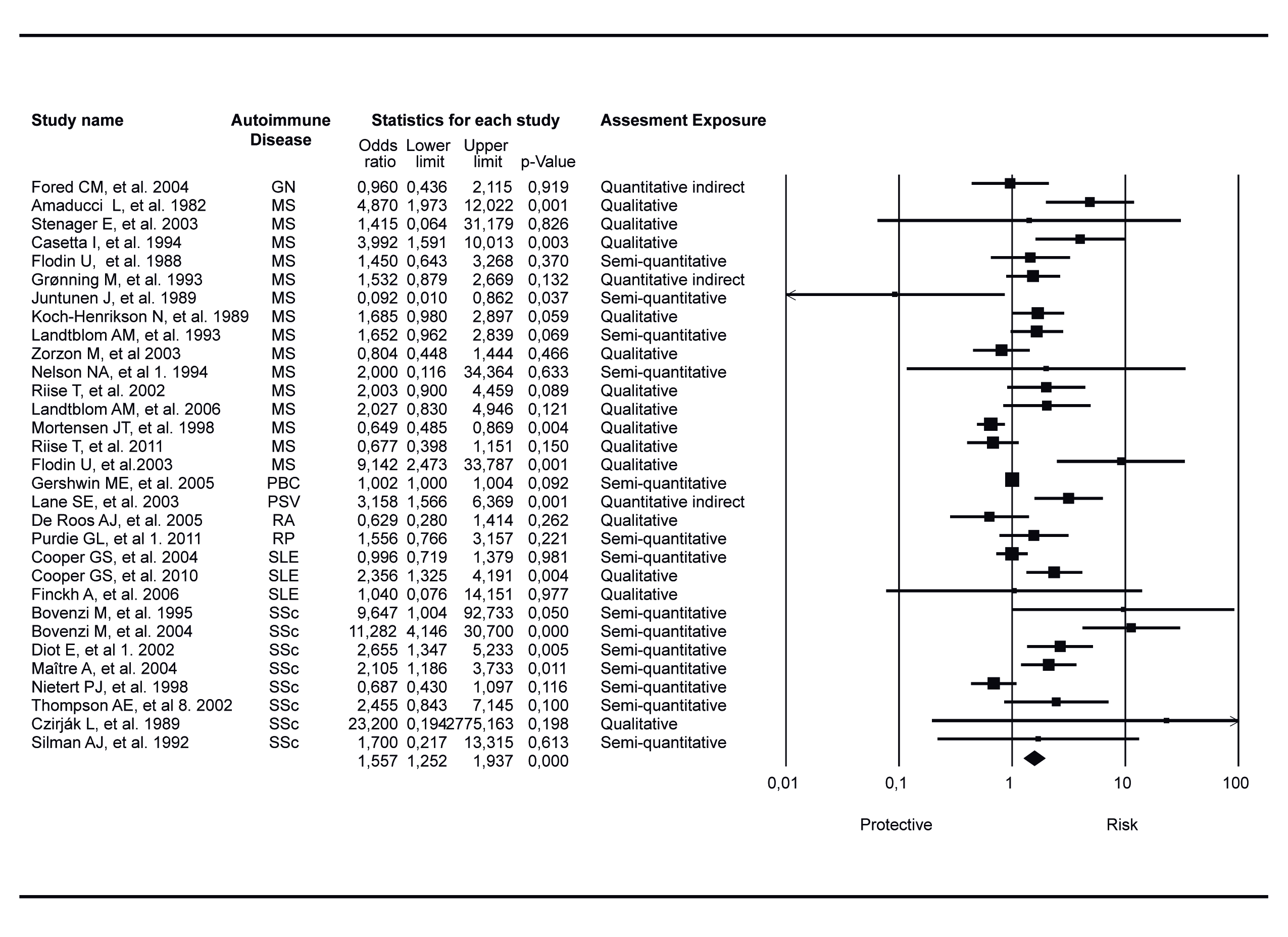

Supplement: Figure S21 — Forest plot of supplementary meta-analyses. Final common effect size based on a random model. The studies included and abbreviations are the same as in Figure S1 with the exception of Thompson AE, et al. 2002 8. Exposition to Urea formaldehyde. (TIF) [file pone.0051506.s021.tif]

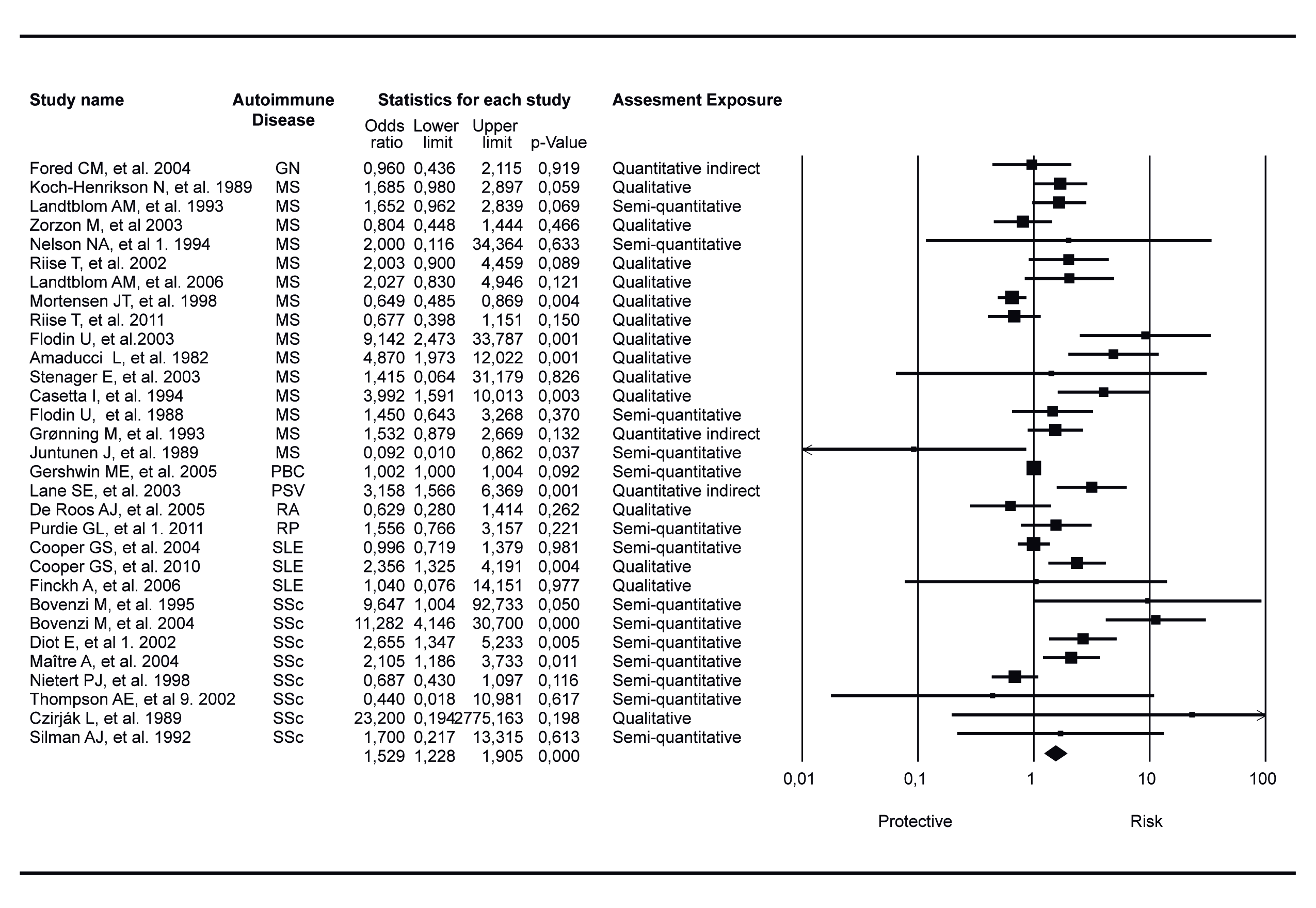

Supplement: Figure S22 — Forest plot of supplementary meta-analyses. Final common effect size based on a random model. The studies included and abbreviations are the same as in Figure S1 with the exception of Thompson AE, et al. 2002 9. Exposition to Meta-phenylenediamene. (TIF) [file pone.0051506.s022.tif]

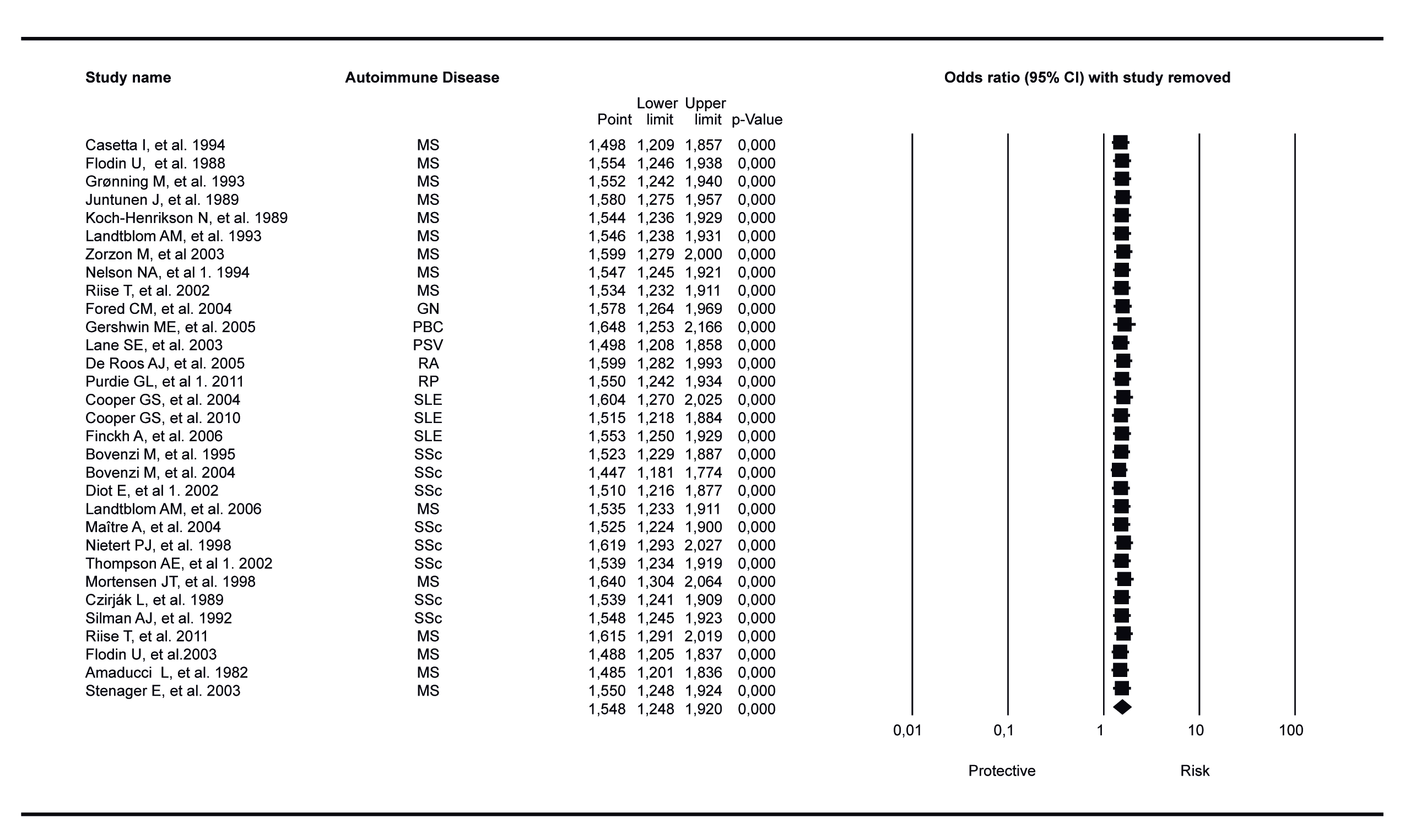

Supplement: Figures S23 — Sensitivity analysis. Footnote: Odds Ratio (95%CI) excluding one study at a time. CI: confidence interval. Diot, et al 1: organic solvent as a whole; Thompson AE, et al 1: turpentine exposure (the most significant result); Purdie GL, et al 1: confirmed RP population; Nelson NA, et al 1. 1994: disabled population. (TIF) [file pone.0051506.s023.tif]

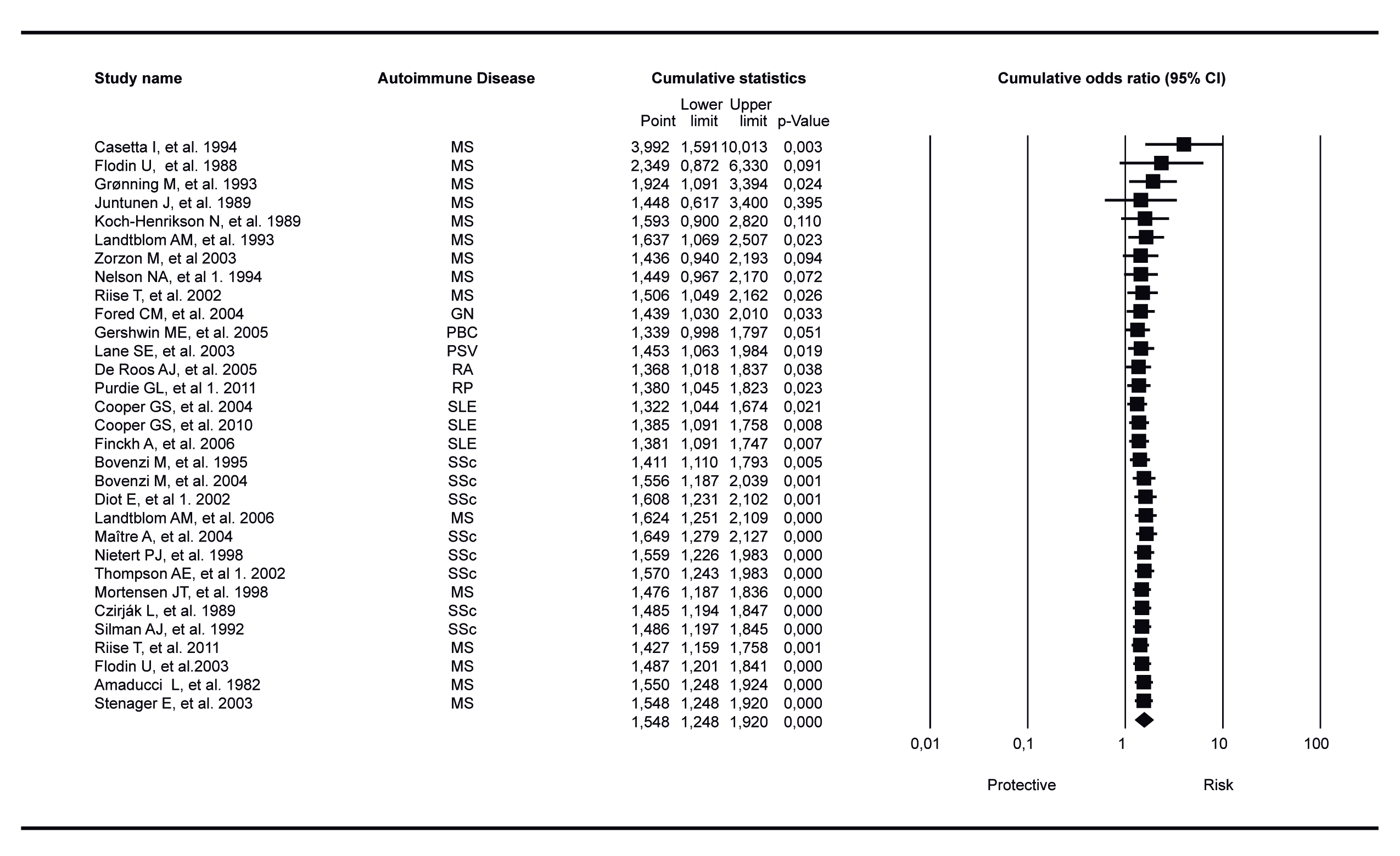

Supplement: Figures S24 — Cumulative analysis. Footnote: Odds Ratio (95%CI) The most relevant outcome per author was included. CI: confidence interval. Diot, et al 1: organic solvent as a whole; Thompson AE, et al 1: turpentine exposure (the most significant result); Purdie GL, et al 1: confirmed RP population; Nelson NA, et al 1. 1994: disabled population. (TIF) [file pone.0051506.s024.tif]

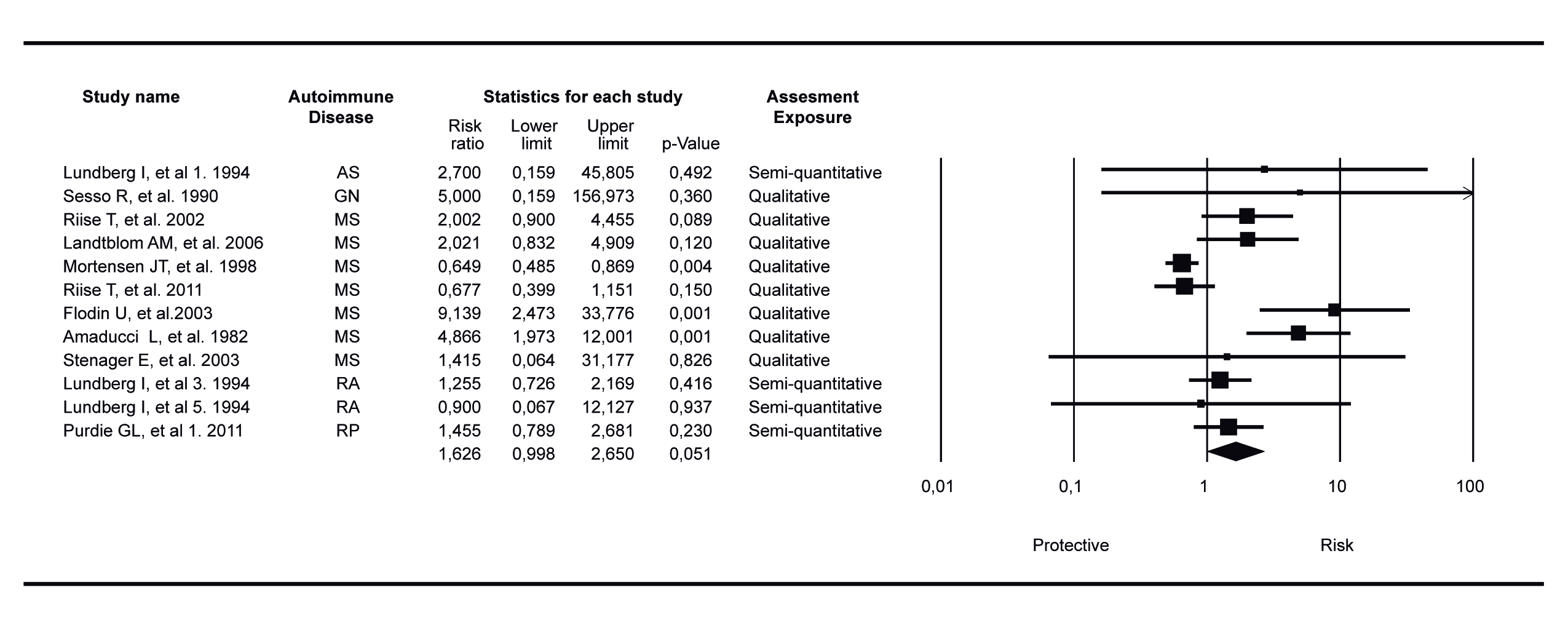

Supplement: Figure S25 — Forest plot of studies showing RR data and raw data from cohort studies. Footnote: final common effect size based on a random model. Risk Ratio (95%CI). CI: confidence interval; AS: Ankylosing spondylitis; GN: glomerulonephritis; MS: multiple sclerosis; RA: rheumatoid arthritis; RP: Raynaud disease; SLE: systemic lupus erythematosus; SSc: systemic sclerosis. Lundberg I, et al. 1 1994 painters AS; Lundberg I, et al. 3 1994 Substantial RA men; Lundberg I, et al. 5 1994 substantial RA women; Purdie GL, et al 1: confirmed RP population. (TIF) [file pone.0051506.s025.tif]

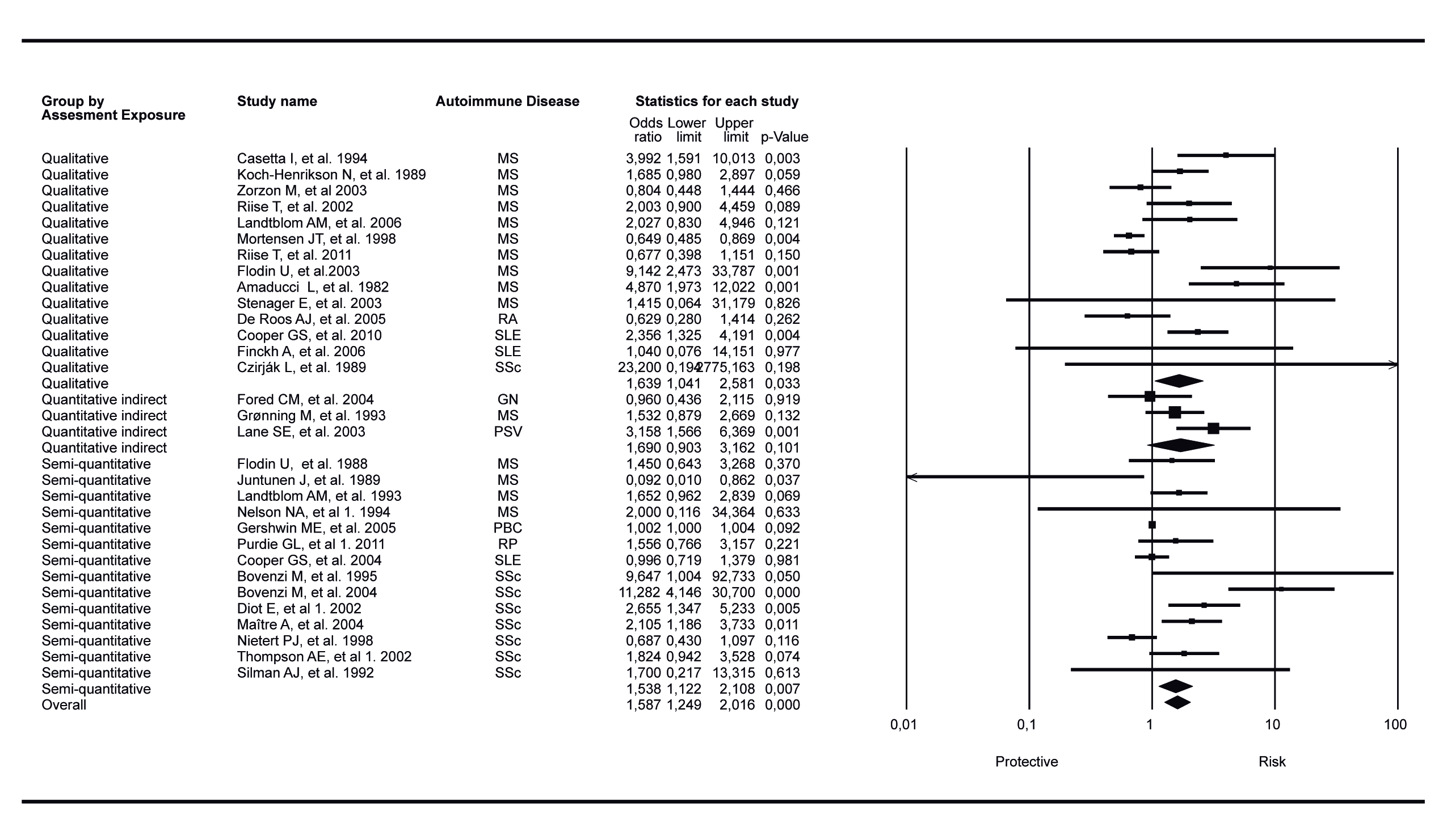

Supplement: Figure S26 — Forest plot of studies showing OR data according to the exposure assessment category. Footnote: Odds Ratio (95%CI). The most relevant outcome per author was included. GN: glomerulonephritis; MS: multiple sclerosis; PBC: primary biliary cirrhosis; PSV: primary systemic vasculitis; RA: rheumatoid arthritis; RP: Raynaud disease; SLE: systemic lupus erythematosus; SSc: systemic sclerosis. Diot, et al 1: organic solvent as a whole; Thompson AE, et al 1: turpentine exposure (the most significant result); Purdie GL, et al 1: confirmed RP population; Nelson NA, et al 1. 1994: disabled population. (TIF) [file pone.0051506.s026.tif]

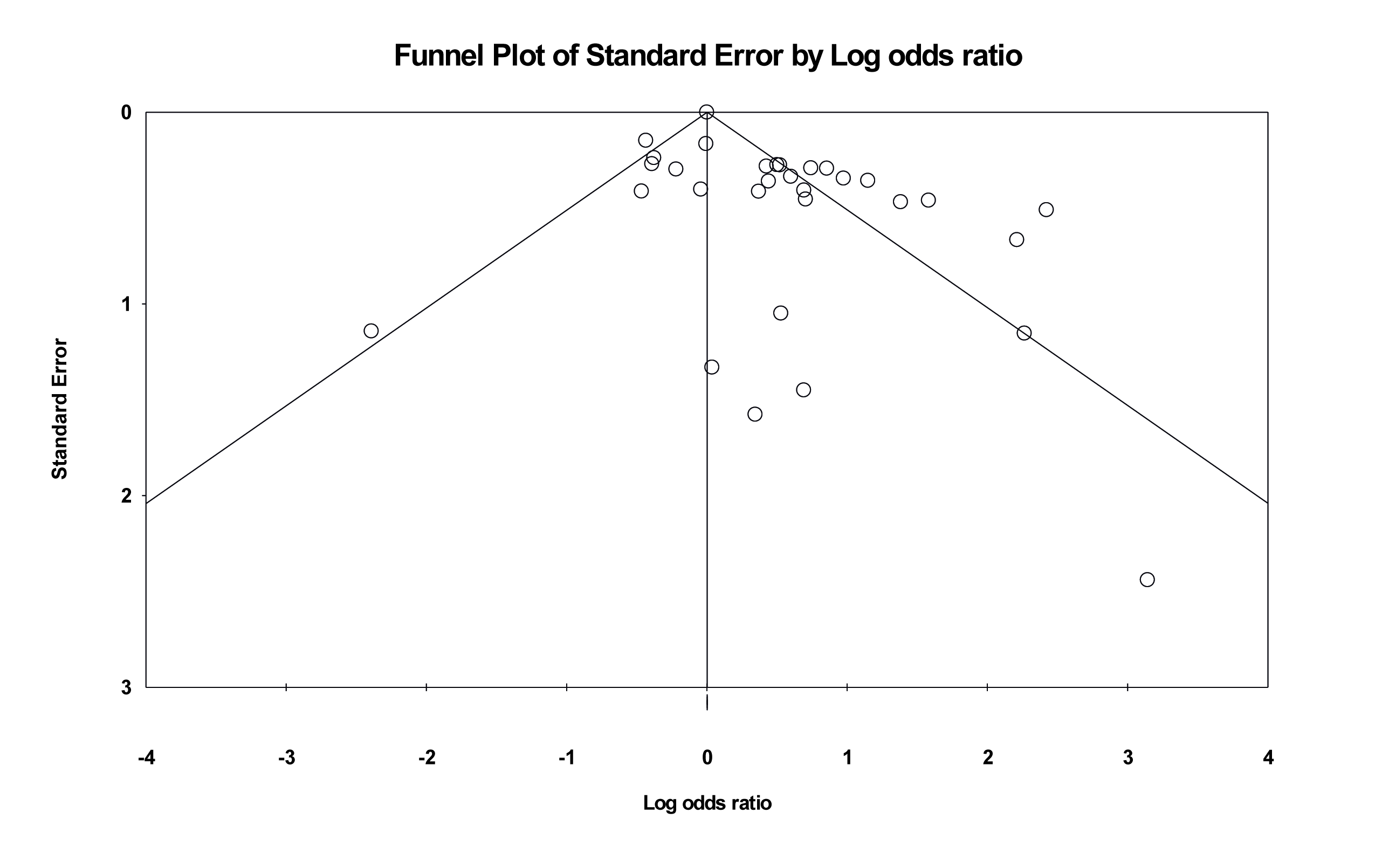

Supplement: Figure S27 — Funnel Plot of standard error by log odds ratio. Footnote: X-axis: Log odds ratio. Y-axis: Standard Error. (TIF) [file pone.0051506.s027.tif]

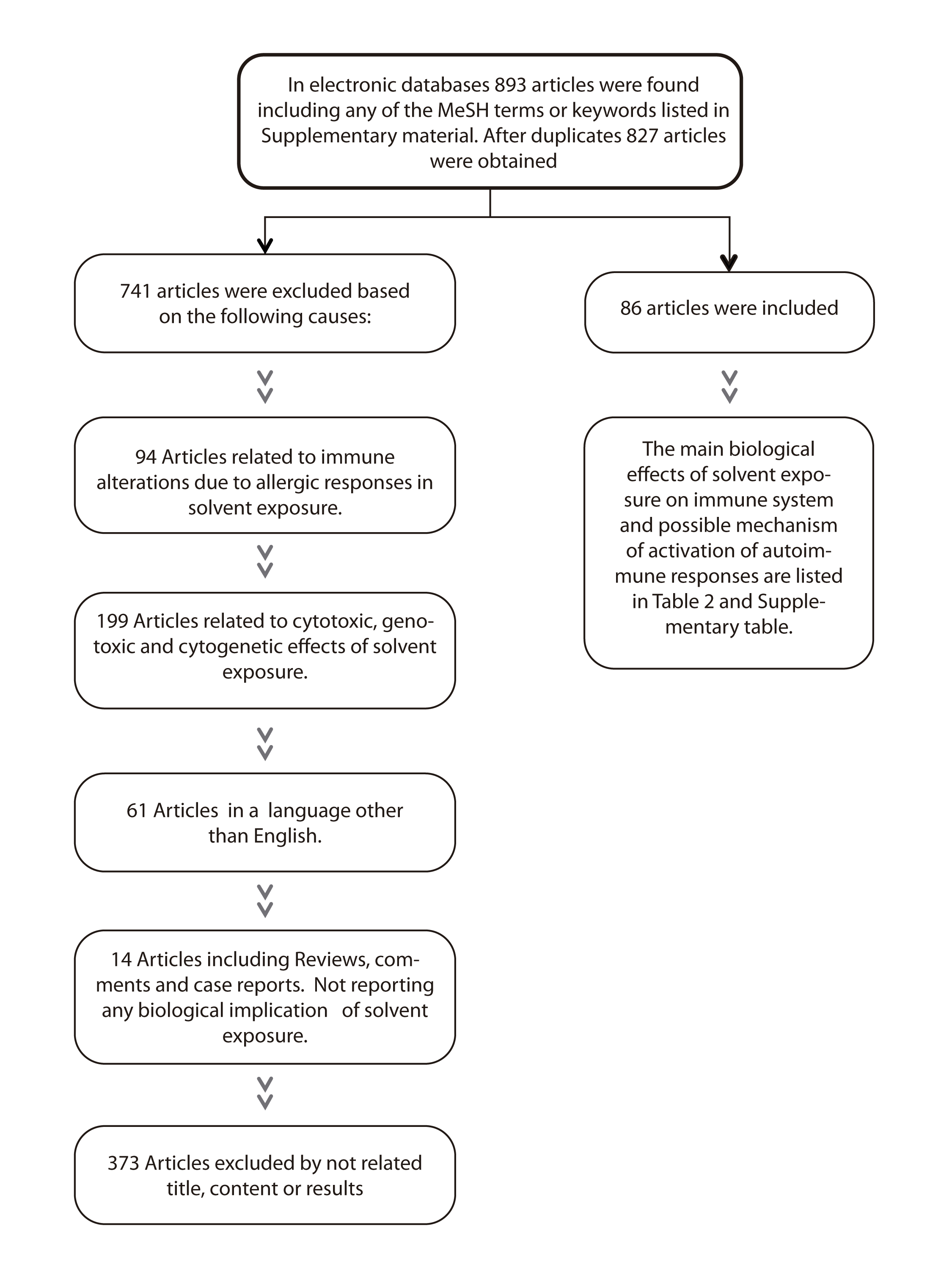

Supplement: Figure S28 — Systematic Review Results for OSs molecular mechanisms related to responses of immune system and ADs. Footnote: ADs: Autoimmune Diseases. (TIF) [file pone.0051506.s028.tif]
