# Supplementary material for: Organic Solvents as Risk Factor for Autoimmune Diseases: A Systematic Review and Meta-Analysis
Source: PLoS One. 2012 Dec 19;7(12):e51506. doi: 10.1371/journal.pone.0051506 (PMC3526640; doi:10.1371/journal.pone.0051506)
Supplement: Table S1 — Studies not included in the Meta-analysis. Footnote: AD: Autoimmune Disease; C-C: Case Control Study; OS: Organic Solvent; SSc: Systemic Sclerosis or Scleroderma; SLE: Systemic Lupus Erythematous; MS: Multiple Sclerosis; PSV: Primary systemic vasculitis; RA: Rheumatoid Arthritis; PBC: Primary Biliary Cirrhosis; GN: Glomerulonephritis; y/o: years old; VC: vinyl chloride; TCE: trichloroethylene; PCE: Perchlorethylene; RDX: Royal Demolition explosive; EEG: electroencephalographic study; PVC: polyvinyl chloride; ESRD: End Stage Renal Disease. (DOCX) [file pone.0051506.s030.docx]

**Table S1**. Studies not included in the Meta-analysis

| **AUTHOR** | **STUDY TYPE** | **AD** | **SUMMARY** | **Cause of exclusion for Meta-analysis** |
| --- | --- | --- | --- | --- |
| Hsieh, Hui-I , et al. 2007[93] | COHORT | AIH | Effect of the CYP2E1 genotype on VC monomer-induced liver fibrosis among PVC workers. | No data on how many exposed met criteria for AIH |
| Brogren CH, et al. 1986[88] | COHORT | GN | Occupational exposure to OS and its effect on the renal excretion of N-acetyl-beta-D-glucosaminidase. | No data on how many exposed met criteria for autoimmune glomerulonephritis |
| Hotz P, et al. 1993[92] | COHORT | GN | Occupational exposure to hydrocarbons and glomerular damage | No data on how many exposed met criteria for autoimmune glomerulonephritis |
| Jacob S, et al. 2007[95] | COHORT | GN | solvent-related ESRD | No data on how many exposed met criteria for autoimmune glomerulonephritis |
| Jacob S,et al. 2007[94] | COHORT | GN | OS exposure on chronic kidney disease progression | No data on how many exposed met criteria for autoimmune glomerulonephritis |
| Landtblom et al. 2003[82] | C-C | MS | Neurodegeneration in images of the basal ganglia in solvent-exposed patients with MS | No data about controls exposure |
| Hopkins RS, et al. 1991[25] | C-C | MS | MS in Galion, Ohio | No data of the OS exposure |
| Souberbielle BE, et al. 1990[84] | C-C | MS | MS in Paris area | No data of the OS exposure |
| Prince M, et al. 2010[96] | C-C | PBC | Case-control studies of risk factors for primary biliary cirrhosis in two United Kingdom populations | No data of the OS exposure |
| Koischwitz D, et al. 1980[90] | COHORT | RD | Changes of hand angyography in the VC industry workers | Lack of data for the unexposed cohort |
| Sińczuk-Walczak H et al. 1982[91] | COHORT | RD | EEG studies in workers chronically exposed to VC | Lack of data for the unexposed cohort |
| Kilburn KH, et al. 1992[83] | C-C | SLE | Prevalence of symptoms of SLE with chronic exposure to TCE and other chemicals in well water. | No data on how many cases met criteria for AD |
| Hathaway et al. 1977[85] | C-C | SLE | No excess of FANA associated with RDX manufacture and use | No data on how many cases met criteria for AD |
| Povey A, et al. 2001[86] | C-C | SSc | To determine a specific cytochrome P450 allele that increase susceptibility to SSc in subjects exposed to OS. | Lack of data regarding population controls exposure to OS. |
| Goldman JA. 1996[89] | C-C | SSc | 279 consecutive patients with various connective tissue diseases. 12 of 33 with SSc support the role of occupational exposure. | Controls had an AD |
| Magnant J, et al. 2005[97] | C-C | SSc | Relationship between occupational risk factors and severity markers of systemic sclerosis | No data of OS exposure |
| C.M Black, et al. 1983[81] | C-C | SSc-LIKE | As part of a 5-year follow-up study HLA A, B, and DR antigens and anti-centromere and anti-scleroderma-70 antibodies were determined in 44 workers exposed to vinyl chloride | No data on how many cases met criteria for AD |
| Albert DA, et al. 2005[87] | COHORT | WG | Analysis of a cluster of cases | Lack of data for the unexposed cohort |

AD: Autoimmune Disease; C-C: Case Control Study; OS: Organic Solvent; SSc: Systemic Sclerosis or Scleroderma; SLE: Systemic Lupus Erythematous; MS: Multiple Sclerosis; PSV: Primary systemic vasculitis; RA: Rheumatoid Arthritis; PBC: Primary Biliary Cirrhosis; GN: Glomerulonephritis; y/o: years old; VC: vinyl chloride; TCE: trichloroethylene; PCE: Perchlorethylene; RDX: Royal Demolition explosive; EEG: electroencephalographic study; PVC: polyvinyl chloride; ESRD: End Stage Renal Disease
