# Supplementary material for: Organic Solvents as Risk Factor for Autoimmune Diseases: A Systematic Review and Meta-Analysis
Source: PLoS One. 2012 Dec 19;7(12):e51506. doi: 10.1371/journal.pone.0051506 (PMC3526640; doi:10.1371/journal.pone.0051506)
Supplement: Table S2 — Case reports and case series. Footnote: AD: Autoimmune Disease; OS: Organic Solvent; SSc: Systemic Sclerosis or Scleroderma; SLE: Systemic Lupus Erythematous; MS: Multiple Sclerosis; PSV: Primary systemic vasculitis; RA: Rheumatoid Arthritis; RD: Raynaud Disease; PBC: Primary Biliary Cirrhosis; GN: Glomerulonephritis; Anti- GBM: Anti-glomerular basement membrane antibody; PM/DM: Polimiositis/Dermatomiositis; y/o: years old; VC: vinyl chloride; TCE: trichloroethylene; PCE: Perchlorethylene; Jo-1: anti-histidyl-t-RNA synthetase. (DOCX) [file pone.0051506.s031.docx]

**Table S2**. Case reports and case series

| **AUTHOR** | **AD** | **Nº OF CASES** | **SUMMARY** |
| --- | --- | --- | --- |
| Czirják L,1994 [51] | CREST | 1 | 26 y/o female. |
| Fernández J, et al. 2010 [32] | GN | 2 | Two patients with mesangial IgA glomerulonephritis, one worked 12 years as a welder, and the other 23 years exposed to a wide number of OS |
| Savige JA, et al. 1989 [33] | GN | 3 | 3 of 6 patients with anti-GBM glomerulonephritis and immune complex were exposed to OS |
| Reis J, et al. 2001 [37] | MS | 1 | A case of multiple sclerosis triggered by OS |
| Amaducci L, et al 1978. [38] | MS | 1 | A case of MS possibly precipitated by OS |
| Ohtsuka T. 2009 [40] | PM/DM | 1 | OS-induced myopathy simulating eosinophilic fasciitis and/or DM. |
| Serratrice J, et al. 2001[41] | PM/DM | 1 | A case of PM with Jo-1 antibody syndrome following extensive VC exposure. |
| Brautbar N, et al. 2004 [34] | PSV | 2 | A 44 y/o electrician with Takayasu Arteritis, and a 51 y/o painter and construction worker with microscopic polyangiitis following exposure to OS |
| Magnavita N, et al. 1986 [39] | PSV | 1 | A case of "vinylic purpur" with increased circulating immune complexes and anti-smooth muscle autoantibodies |
| Sparrow GP. 1977.[50] | RP | 1 | A 19 year old male worked cleaning the drum which had contained PCE with polymyopathy, acrocyanosis and mild hepatic disorder. |
| Petkova V, 1992[36] | RP | 12 | Observed clinical picture of vinyl-chloride disease after exposure from 5 to 34 years to toxic concentrations |
| A. Benzarti, et al. 2010 [42] | SSc | 1 | After 6 years of exposition, an industry covering flying worker, developed SSc |
| Hinnen U, et AL. 1995 [43] | SSc | 1 | A patient who exposed to PCE for one year while operating a metal degreaser developed morphea-like scleroderma suspected. |
| Calvani N, et al. 2001 [35] | SSc | 3 | Familial SSc: 3 sisters working in a dry cleaner's shop and exposed to occupational inhalation of OS |
| Garcia-Zamalloa AM, 1994.[44] | SSc | 1 | A 56 y/o male developed SSc after 23 years working in the rubber transformation section of a tire factory |
| Bottomley WW,1993[45] | SSc | 1 | A sclerodermatous syndrome with unusual features following prolonged occupational exposure to OS. |
| Tibon-Fisher O, 1992[46] | SSc | 1 | Occupational SSc due to OS exposure during 13 years of work renovating carburetors |
| Brasington RD Jr,1991.[47] | SSc | 1 | SSc associated with 15 years in a foundry, cutaneous contact with multiple OS |
| Karamfilov T, 2003.[48] | SSc | 1 | Pansclerotic Porphyria Cutanea Tarda after chronic exposure. |
| Pralong P, et al. 2009.[49] | SSc | 1 | Diffuse SSc after occupational exposure. |

AD: Autoimmune Disease; OS: Organic Solvent; SSc: Systemic Sclerosis or Scleroderma; SLE: Systemic Lupus Erythematous; MS: Multiple Sclerosis; PSV: Primary systemic vasculitis; RA: Rheumatoid Arthritis; RD: Raynaud Disease; PBC: Primary Biliary Cirrhosis; GN: Glomerulonephritis; Anti- GBM: Anti-glomerular basement membrane antibody; PM/DM: Polimiositis/ Dermatomiositis; y/o: years old; VC: vinyl chloride; TCE: trichloroethylene; PCE: Perchlorethylene; Jo-1: anti-histidyl-t-RNA synthetase
