# Supplementary material for: Organic Solvents as Risk Factor for Autoimmune Diseases: A Systematic Review and Meta-Analysis
Source: PLoS One. 2012 Dec 19;7(12):e51506. doi: 10.1371/journal.pone.0051506 (PMC3526640; doi:10.1371/journal.pone.0051506)
Supplement: Table S3 — Search strategy related to solvent exposure and immune alterations. (DOCX) [file pone.0051506.s032.docx]

**Supplementary Table S3: Search strategy related to solvent exposure and immune alterations**

**Search Organic Solvent Add data Pubmed Search Articles retrieved**

1 Tetrachloroethylene[Mesh] solvent exposure ((Tetrachloroethylene[Mesh]) AND

and immune alterations solvent exposure and immune alterations) 9

2 Trichloroethylene[Mesh] Immune System[Mesh] ((Trichloroethylene[Mesh]) AND Immune System[Mesh]) 43

3 Trichloroethanes[Mesh] Immune System[Mesh] ((Trichloroethanes[Mesh]) AND Immune System[Mesh]) 0

4 Perchlorethylene[Mesh] Immune System[Mesh] ((Perchlorethylene[Mesh]) AND Immune System[Mesh]) 9

5 Toluene[Mesh] Immune System[Mesh] ((Toluene[Mesh]) AND Immune System[Mesh]) 331

6 Vinyl Chloride[Mesh] Immune System[Mesh] ((Vinyl Chloride[Mesh]) AND Immune System[Mesh]) 19

7 Acetone[Mesh] Immune System[Mesh] ((Acetone[Mesh]) AND Immune System[Mesh]) 72

8 Ethylacetate[Mesh] Immune System[Mesh] ((Ethylacetate[Mesh]) AND Immune System[Mesh]) 0

9 Turpentine[Mesh] Immune System[Mesh] ((Turpentine[Mesh]) AND Immune System[Mesh]) 30

10 Benzene[Mesh] Immune System[Mesh] ((Benzene[Mesh]) AND Immune System[Mesh]) 268

11 5-Hydroxytryptophan[Mesh] Immune System[Mesh] ((5-Hydroxytryptophan[Mesh]) AND Immune System[Mesh]) 25

12 Diethylpropion[Mesh] Immune System[Mesh] ((Diethylpropion[Mesh]) AND Immune System[Mesh]) 0

13 Fenfluramine[Mesh] Immune System[Mesh] ((Fenfluramine[Mesh]) AND Immune System[Mesh]) 11

14 Hair Dyes[Mesh] Immune System[Mesh] ((Hair Dyes[Mesh]) AND Immune System[Mesh]) 12

15 Hexane[Mesh] Immune System[Mesh] ((Hexane[Mesh]) AND Immune System[Mesh]) 0

16 Urea Formaldehyde Immune System[Mesh] ((Urea Formaldehyde) AND Immune System[Mesh]) 11

17 White Spirit Immune System[Mesh] ((White Spirit) AND Immune System[Mesh]) 0

18 Nail Polish Immune System[Mesh] ((Nail Polish) AND Immune System[Mesh]) 0

19 Tetrachloroethylene[Mesh] Autoimmunity[Mesh] ((Tetrachloroethylene[Mesh]) AND Autoimmunity[Mesh]) 0

20 Trichloroethylene[Mesh] Autoimmunity[Mesh] ((Trichloroethylene[Mesh]) AND Autoimmunity[Mesh]) 8

21 Trichloroethanes[Mesh] Autoimmunity[Mesh] ((Trichloroethanes[Mesh]) AND Autoimmunity[Mesh]) 0

22 Perchlorethylene[Mesh] Autoimmunity[Mesh] ((Perchlorethylene[Mesh]) AND Autoimmunity[Mesh]) 0

23 Toluene[Mesh] Autoimmunity[Mesh] ((Toluene[Mesh]) AND Autoimmunity[Mesh]) 0

24 Vinyl Chloride[Mesh] Autoimmunity[Mesh] ((Vinyl Chloride[Mesh]) AND Autoimmunity[Mesh]) 0

25 Acetone[Mesh] Autoimmunity[Mesh] ((Acetone[Mesh]) AND Autoimmunity[Mesh]) 0

26 Ethylacetate[Mesh] Autoimmunity[Mesh] ((Ethylacetate[Mesh]) AND Autoimmunity[Mesh]) 0

27 Turpentine[Mesh] Autoimmunity[Mesh] ((Turpentine[Mesh]) AND Autoimmunity[Mesh]) 0

28 Benzene[Mesh] Autoimmunity[Mesh] ((Benzene[Mesh]) AND Autoimmunity[Mesh]) 2

29 5-Hydroxytryptophan[Mesh] Autoimmunity[Mesh] ((5-Hydroxytryptophan[Mesh]) AND Autoimmunity[Mesh]) 0

30 Diethylpropion[Mesh] Autoimmunity[Mesh] ((Diethylpropion[Mesh]) AND Autoimmunity[Mesh]) 0

31 Fenfluramine[Mesh] Autoimmunity[Mesh] ((Fenfluramine[Mesh]) AND Autoimmunity[Mesh]) 0

32 Hair Dyes[Mesh] Autoimmunity[Mesh] ((Hair Dyes[Mesh]) AND Autoimmunity[Mesh]) 0

33 Hexane[Mesh] Autoimmunity[Mesh] ((Hexane[Mesh]) AND Autoimmunity[Mesh]) 0

34 Urea Formaldehyde Autoimmunity[Mesh] ((Urea Formaldehyde) AND Autoimmunity[Mesh]) 0

35 White Spirit Autoimmunity[Mesh] ((White Spirit) AND Autoimmunity[Mesh]) 0

36 Nail Polish Autoimmunity[Mesh] ((Nail Polish) AND Autoimmunity[Mesh]) 0

37 Tetrachloroethylene[Mesh] "Autoimmune Diseases" ((Tetrachloroethylene[Mesh]) AND "Autoimmune Diseases") 1

38 Trichloroethylene[Mesh] "Autoimmune Diseases" ((Trichloroethylene[Mesh]) AND "Autoimmune Diseases") 18

39 Trichloroethanes[Mesh] "Autoimmune Diseases" ((Trichloroethanes[Mesh]) AND "Autoimmune Diseases") 0

40 Perchlorethylene[Mesh] "Autoimmune Diseases" ((Perchlorethylene[Mesh]) AND "Autoimmune Diseases") 1

41 Toluene[Mesh] "Autoimmune Diseases" ((Toluene[Mesh]) AND "Autoimmune Diseases") 3

42 Vinyl Chloride[Mesh] "Autoimmune Diseases" ((Vinyl Chloride[Mesh]) AND "Autoimmune Diseases") 6

43 Acetone[Mesh] "Autoimmune Diseases" ((Acetone[Mesh]) AND "Autoimmune Diseases") 2

44 Ethylacetate[Mesh] "Autoimmune Diseases" ((Ethylacetate[Mesh]) AND "Autoimmune Diseases") 0

45 Turpentine[Mesh] "Autoimmune Diseases" ((Turpentine[Mesh]) AND "Autoimmune Diseases") 2

46 Benzene[Mesh] "Autoimmune Diseases" ((Benzene[Mesh]) AND "Autoimmune Diseases") 5

47 5-Hydroxytryptophan[Mesh] "Autoimmune Diseases" ((5-Hydroxytryptophan[Mesh]) AND "Autoimmune Diseases") 0

48 Diethylpropion[Mesh] "Autoimmune Diseases" ((Diethylpropion[Mesh]) AND "Autoimmune Diseases") 0

49 Fenfluramine[Mesh] "Autoimmune Diseases" ((Fenfluramine[Mesh]) AND "Autoimmune Diseases") 0

50 Hair Dyes[Mesh] "Autoimmune Diseases" ((Hair Dyes[Mesh]) AND "Autoimmune Diseases") 3

51 Hexane[Mesh] "Autoimmune Diseases" ((Hexane[Mesh]) AND "Autoimmune Diseases") 0

52 Urea Formaldehyde "Autoimmune Diseases" ((Urea Formaldehyde) AND "Autoimmune Diseases") 0

53 White Spirit "Autoimmune Diseases" ((White Spirit) AND "Autoimmune Diseases") 0

54 Nail Polish "Autoimmune Diseases" ((Nail Polish) AND "Autoimmune Diseases") 2

**TOTAL 893**
