# Supplementary material for: Organic Solvents as Risk Factor for Autoimmune Diseases: A Systematic Review and Meta-Analysis
Source: PLoS One. 2012 Dec 19;7(12):e51506. doi: 10.1371/journal.pone.0051506 (PMC3526640; doi:10.1371/journal.pone.0051506)
Supplement: Table S4 — Effects of the exposition to organic solvents on experimental models. (DOCX) [file pone.0051506.s033.docx]

**Supplementary Table S4: Effects of the exposition to organic solvents on experimental models and on humans**

**Organic Solvent Effect on Phenotype Model References**

TCE Proliferation of CD8+ T cells rather CD4+ T cells Mice (BALB/c) [1]

Benzene-Toluene-Xylene High exposure decrease levels of IL-10, TNFα and IL-12 Human [2]

Benzene Impaired function of T cell by alters CD3 expression Human [3]

Toluene Early exposure leads to T cells reduction and suppressed of IL-12, Tbet, FOXP3 production. Mice (C3H/HeN) [4]

Benzene "Effects on TLR signaling pathway, oxidative

phosphorylation, B-cell receptor signaling pathway,

apoptosis and T-cell receptor signaling" Human [5]

Toluene Thymocytes activation and production of IL-12 Mice (C3H/HeN) [6]

Benzene Elevated production of ROS and increased levels

of CD4+ T cells subset and immunoglobulins. Human [7]

Hair dye Induced local inflammation and cellular infiltration.

Exposure promotes T-cell and B cell activation and

production of IFNγ, TNFα, IL-1β and IL-17. Later

a Treg responses was observed. Mice (C57BL/6 and CBA/Ca) [8, 9]

Toluene Low exposure increased FOXP3, STAT5 and STAT6

mRNA expression. Mice (C57BL/10) [10]

TCE Reduction of T cell, B cell, NK cell population. Human [11]

Benzene Formation of proteins adducts and ROS production Rats (Sprague-Dawley) [12]

Toluene Early postnatal exposure leads to suppression of

Th1/Th2 responses. Mice (BALB/c) [13]

TCE Presence of autoantigens in serum of exposed workers Human [14]

TCE Exposure promotes protein oxidation, iNOS production

and autoimmune exacerbation. Mice (MRL +/+) [15]

Benzene Exposure in workers leads to a particular clonal

expansion of TCRVbeta subfamilies. Human [16]

TCE Alteration in expression of hepatic genes associated

with immunity and inflammation. Promotion of

autoimmune hepatitis. Mice (MRL +/+) [17]

TCE The exposure not promotes progression of autoimmunity

rather increased the expression of markers associated

with autoimmunity. Mice (NZBWF1 and B6C3F1) [18]

Benzene Decrease in T-cell receptor excision DNA circles impaired

the T-cells immune function. Human [19]

Benzene Changes in expression of genes involved in apoptosis and

lipid metabolism (CXCL16, ZNF331, JUN and PF4). Human [20, 21]

Hydroquinone Inhibition of inflammatory responses by suppression of

proinflammatory cytokines (TNFα, IL-1β, IL-6), NOS and Mice (C57BL/6) and Cell Line

ROS production. (RAW 264.7 and U937) [22, 23]

TCE Exposure to TCE does not accelerate autoimmune responses Mice (MRL +/+) [24]

TCE Prenatal and early exposure modulate T cell developmental,

inflammatory responses and neurobehavioral deficiencies. Mice (MRL +/+) [25]

Benzene Promotes expression of proinflammatory cytokines

(TNFα,IL-6) and suppression of IL-10. Human [26]

Turpentine Upregulation of expression of proinflammatory cytokines

IL-6, TNFα and IL-1β Rat (Wistar) [27]

Benzene Suppression of IgM and IgG production and reduction of

CD4+ T cells Human [28]

TCE Chronic exposure generates skin inflammation, alopecia

and proinflammatory cytokines production Mice (MRL +/+) [29]

Benzene Apoptosis induction of lymphoyites Rat (Wistar) [30]

Benzene Marked DNA damage in T and B cells Rats (Sprague-Dawley) [31]

Benzene Changes in expression of several genes involve in

metabolism pathways Rat (F344/CrlBR) [32]

Toluene Decrease of lymphocyte life span Human [33]

TCAA Activation of TFs involves in Th1 activation and proliferation Mice (MRL +/+) [34]

Hair Dye Acute exposure generate DNA damages in peripheral

lymphocytes Human [35]

Benzene Formation of DNA and protein adducts Mice (B6C3F1, DBA/2, C57BL/6)

and Rats (Fischer) [36]

Hair dye No evidence between the use of hair dyes and autoimmune

diseases such as SLE Human [37]

Styrene-Benzene-Polyciclic Changes in expression of surface antigens on lymphocytes

Hidrocarbons mainly in Treg cells Human [38]

Toluene Decrease in cellular number of T cells and NK cells and

increase in B cells population with variables levels among

exposure days. Human [39]

PCE Presence of protein adducts in the liver Mice (MRL-lpr/lpr and MRL +/+ ) [40]

Diesel Exhaust Particles Increase in the expression of inflammatory cytokines and

(Benzene-derived compounds) chemokines in the lung. Human bronchial epithelial cell (BEAS-2B) [41]

DCAC Production of ROS leads to increases of lipid-peroxidation

and presence of anti-malondialdehyde autoantibodies Mice (MRL+/+ ) [42]

Hydroquinone Stimulation of myeloid lineage differentiation Myeloblast Cells [43]

Vinyl Chloride Activation of microchimeric cells derived of maternal or

fetal sources promotes cell division and this may be related

to dermal inflammation and fibrosis in SSc Mice (BALB/cJ) [44]

Toluene Presence of T-cell-derived antigen-binding molecules

associated to Toluene metabolism Human [45]

Benzene Depression in the number of B cells in worker exposed Human [46]

TCE The alteration of CD4+ T cells responses is dependent on

metabolism of TCE by CYP2E1 Mice (MRL+/+) [47]

TCE and PCE Increase of the oxidative DNA damage Rat (Fischer) [48]

Hexane-Toluene-Methyl No changes in the activity of NK cells or production

Ethyl Ketone of IL-12 cytokine Human [49]

Hydroquinone and Benzoquinone This metabolites can act as hapten and stimulate a

inflammatory immune response Mice (C57BL/6J and BALB/c) [50]

Benzene Stimulation in vitro with PMA and in vivo with LPS in

mice treated with benzene leads to increase ROS/RNS Mice

production and inflammatory responses [51]

Benzene Presence of autoantibodies against Heat shock or stress

proteins (Hsps) in patients with benzene poising Human [52]

Benzene The hydroxylation and nitration of benzene promotes the

toxic effects of this compound Human Neutrophils [53]

Hydroquinone Repress transcriptional activities of NF-κB and inhibit IL-2

cytokine production in CD4+ T cells Human CD4+ T cells [54]

Arsenic-Cadmium-Lead-Benzene The concomitant administration of contaminant water

and TCE with a poor vitamin dietary leads to decrease in weight

gain and cellular and humoral immunity Chicken (Peterson/Avian fast-feathering) [55]

Benzene Low exposure generates reduction of B cell

(Femoral and splenic) and T cell (Splenic and Thymic)

subpopulations Mice (B6C3F1/CrlBR and B6C3F1) [56,57]

Hydroquinone Stimulation of granulocyte differentiation by activation

of granulocyte-macrophage progenitor cells Mice (C57BL6) [58,59]

Benzoquinone Prevents the processing of preIL-1α y β to active cytokines Mice (C57BL/6J), B1 Cell Line and

in monocytes and also cause apoptosis of the hematopoietic Human PBMCs [60,61,62,63]

progenitor cells

Xylene and Benzene Xylene induce an increase in neutrophils levels while

benzene cause a reduction in lymphocyte population Rat (Wistar) [64]

Benzene Stimulates the production of NOS by leukocytes and

potentiates to inflammatory responses against with reactive

intermediaries Mice (BALB/c) [65]

Benzene Reduced the expression of IL-12 cytokine Mice (C57 BL/6) [66]

TCE Inhibit cytotoxic activities by NK cells Rat (Sprague-Dawley) and Mice (B6C3F1) [67]

Benzene Stimulate the production of TNFα and slightly increase

IL-1 production Mice (BALB/c) [68]

TCE Reduction of lymphocytes, monocytes and neutrophils Mice ( C57/BL/6 x DBA/2) [69]

**TCE:** Trichloroethylene, **IL:** Interleukin, **TNFα:** Tumor, Necrosis Factor α, **T-bet:** T-cell-specific T-box transcription factor, **TLR:** Toll Like Receptor, **IFNγ:** Interferon γ, **ROS:** Reactive Oxygen Species, **NO:** Nitric oxide, **iNOS:** inducible Nitric Oxide Synthase, **CD:** Cluster of differentiation, **FOXP3:** Forkhead box protein P3, **STAT:** Signal Transducer and Activator of Transcription, **NK:** Natural killer, **CXCL16:** Chemokine (C-X-C motif) ligand 16, **ZNF331:** Zinc finger protein 331, **PF4:** Platelet factor-4, **TCAA:** Trichloroacetaldehyde, **SLE:** Sytemic Lupus Erythematosus, **DCAC:** Dichloroacetyl chloride, **SSc:** Systemic Sclerosis, **CYP2E1:** Cytochrome P450 2E1, **PCE:** Perchlorethylene, **PMA:** Phorbol myristate acetate, **LPS:** Lipopolysaccharide, **NF-κB:** Nuclear Factor κappa B, **TCR:** T cell Receptor.

**References Supplementary Table S4**

1. Kobayashi R, Nakanishi T, Nagase H (2012) Trichloroethylene enhances TCR-CD3-induced proliferation of CD8(+) rather than CD4(+) T cells. The Journal of toxicological sciences 37: 381–387. Available: http://www.ncbi.nlm.nih.gov/pubmed/22467029. Accessed 19 October 2012.

2. Haro-García LC, Juárez-Pérez CA, Aguilar-Madrid G, Vélez-Zamora NM, Muñoz-Navarro S, et al. (2012) Production of IL-10, TNF and IL-12 by peripheral blood mononuclear cells in Mexican workers exposed to a mixture of benzene-toluene-xylene. Archives of medical research 43: 51–57. Available: http://www.ncbi.nlm.nih.gov/pubmed/22300681. Accessed 19 October 2012.

3. Li B, Niu Y, Liu S, Yu W, Chen J, et al. (n.d.) A change in CD3γ, CD3δ, CD3ϵ, and CD3ζ gene expression in T-lymphocytes from benzene-exposed and benzene-poisoned workers. Journal of immunotoxicology 9: 160–167. Available: http://www.ncbi.nlm.nih.gov/pubmed/22214187. Accessed 19 October 2012.

4. Win-Shwe T-T, Kunugita N, Nakajima D, Yoshida Y, Fujimaki H (2012) Developmental stage-specific changes in immunological biomarkers in male C3H/HeN mice after early life toluene exposure. Toxicology letters 208: 133–141. Available: http://www.ncbi.nlm.nih.gov/pubmed/22057034. Accessed 19 October 2012.

5. McHale CM, Zhang L, Lan Q, Vermeulen R, Li G, et al. (2011) Global gene expression profiling of a population exposed to a range of benzene levels. Environmental health perspectives 119: 628–634. Available: http://www.pubmedcentral.nih.gov/articlerender.fcgi?artid=3094412&tool=pmcentrez&rendertype=abstract. Accessed 19 October 2012.

6. Liu J, Yoshida Y, Kunugita N, Noguchi J, Sugiura T, et al. (2010) Thymocytes are activated by toluene inhalation through the transcription factors NF-κB, STAT5 and NF-AT. Journal of applied toxicology : JAT 30: 656–660. Available: http://www.ncbi.nlm.nih.gov/pubmed/20981857. Accessed 19 October 2012.

7. Uzma N, Kumar BS, Hazari MAH (2010) Exposure to benzene induces oxidative stress, alters the immune response and expression of p53 in gasoline filling workers. American journal of industrial medicine 53: 1264–1270. Available: http://www.ncbi.nlm.nih.gov/pubmed/20886531. Accessed 19 October 2012.

8. Rubin IMC, Dabelsteen S, Nielsen MM, White IR, Johansen JD, et al. (2010) Repeated exposure to hair dye induces regulatory T cells in mice. The British journal of dermatology 163: 992–998. Available: http://www.ncbi.nlm.nih.gov/pubmed/20716223. Accessed 19 October 2012.

9. Bonefeld CM, Larsen JM, Dabelsteen S, Geisler C, White IR, et al. (2010) Consumer available permanent hair dye products cause major allergic immune activation in an animal model. The British journal of dermatology 162: 102–107. Available: http://www.ncbi.nlm.nih.gov/pubmed/19785606. Accessed 19 October 2012.

10. Fujimaki H, Win-Shwe T-T, Yamamoto S, Kunugita N, Yoshida Y, et al. (2010) Different sensitivity in expression of transcription factor mRNAs in congenic mice following exposure to low-level toluene. Inhalation toxicology 22: 903–909. Available: http://www.ncbi.nlm.nih.gov/pubmed/20583897. Accessed 19 October 2012.

11. Lan Q, Zhang L, Tang X, Shen M, Smith MT, et al. (2010) Occupational exposure to trichloroethylene is associated with a decline in lymphocyte subsets and soluble CD27 and CD30 markers. Carcinogenesis 31: 1592–1596. Available: http://www.pubmedcentral.nih.gov/articlerender.fcgi?artid=2930801&tool=pmcentrez&rendertype=abstract. Accessed 19 October 2012.

12. Lau SS, Kuhlman CL, Bratton SB, Monks TJ (2010) Role of hydroquinone-thiol conjugates in benzene-mediated toxicity. Chemico-biological interactions 184: 212–217. Available: http://www.pubmedcentral.nih.gov/articlerender.fcgi?artid=2846198&tool=pmcentrez&rendertype=abstract. Accessed 19 October 2012.

13. Yamamoto S, Win-Shwe T-T, Tin-Tin-Win-Shwe, Yoshida Y, Kunugita N, et al. (2009) Suppression of Th1- and Th2-type immune responses in infant mouse spleen after prenatal and postnatal exposure to low-level toluene and peptidoglycan. Inhalation toxicology 21: 793–802. Available: http://www.ncbi.nlm.nih.gov/pubmed/19645570. Accessed 19 October 2012.

14. Liu J, Xing X, Huang H, Jiang Y, He H, et al. (2009) Identification of antigenic proteins associated with trichloroethylene-induced autoimmune disease by serological proteome analysis. Toxicology and applied pharmacology 240: 393–400. Available: http://www.ncbi.nlm.nih.gov/pubmed/19647757. Accessed 19 October 2012.

15. Wang G, Wang J, Ma H, Khan MF (2009) Increased nitration and carbonylation of proteins in MRL+/+ mice exposed to trichloroethene: potential role of protein oxidation in autoimmunity. Toxicology and applied pharmacology 237: 188–195. Available: http://www.pubmedcentral.nih.gov/articlerender.fcgi?artid=2734328&tool=pmcentrez&rendertype=abstract. Accessed 19 October 2012.

16. Li B, Li Y, Chen S, Yang L, Yu W, et al. (2009) The T-cell receptor Vbeta gene repertoire and clonal expansion from peripheral blood T cells in benzene-exposed workers in China. Hematology (Amsterdam, Netherlands) 14: 106–110. Available: http://www.ncbi.nlm.nih.gov/pubmed/19298723. Accessed 19 October 2012.

17. Gilbert KM, Przybyla B, Pumford NR, Han T, Fuscoe J, et al. (2009) Delineating liver events in trichloroethylene-induced autoimmune hepatitis. Chemical research in toxicology 22: 626–632. Available: http://www.ncbi.nlm.nih.gov/pubmed/19254012. Accessed 19 October 2012.

18. Keil DE, Peden-Adams MM, Wallace S, Ruiz P, Gilkeson GS (2009) Assessment of trichloroethylene (TCE) exposure in murine strains genetically-prone and non-prone to develop autoimmune disease. Journal of environmental science and health Part A, Toxic/hazardous substances & environmental engineering 44: 443–453. Available: http://www.ncbi.nlm.nih.gov/pubmed/19241258. Accessed 19 October 2012.

19. Li B, Li YQ, Yang LJ, Chen SH, Yu W, et al. (2009) Decreased T-cell receptor excision DNA circles in peripheral blood mononuclear cells among benzene-exposed workers. International journal of immunogenetics 36: 107–111. Available: http://www.ncbi.nlm.nih.gov/pubmed/19228219. Accessed 19 October 2012.

20. McHale CM, Zhang L, Lan Q, Li G, Hubbard AE, et al. (2009) Changes in the peripheral blood transcriptome associated with occupational benzene exposure identified by cross-comparison on two microarray platforms. Genomics 93: 343–349. Available: http://www.pubmedcentral.nih.gov/articlerender.fcgi?artid=2693268&tool=pmcentrez&rendertype=abstract. Accessed 19 October 2012.

21. Forrest MS, Lan Q, Hubbard AE, Zhang L, Vermeulen R, et al. (2005) Discovery of novel biomarkers by microarray analysis of peripheral blood mononuclear cell gene expression in benzene-exposed workers. Environmental health perspectives 113: 801–807. Available: http://www.pubmedcentral.nih.gov/articlerender.fcgi?artid=1257610&tool=pmcentrez&rendertype=abstract. Accessed 19 October 2012.

22. Cho JY (2008) Suppressive effect of hydroquinone, a benzene metabolite, on in vitro inflammatory responses mediated by macrophages, monocytes, and lymphocytes. Mediators of inflammation 2008: 298010. Available: http://www.pubmedcentral.nih.gov/articlerender.fcgi?artid=2625402&tool=pmcentrez&rendertype=abstract. Accessed 19 October 2012.

23. Lee JY, Kim JY, Lee YG, Shin WC, Chun T, et al. (2007) Hydroquinone, a reactive metabolite of benzene, reduces macrophage-mediated immune responses. Molecules and cells 23: 198–206. Available: http://www.ncbi.nlm.nih.gov/pubmed/17464197. Accessed 19 October 2012.

24. Peden-Adams MM, Eudaly JG, Lee AM, Miller J, Keil DE, et al. (2008) Lifetime exposure to trichloroethylene (TCE) does not accelerate autoimmune disease in MRL +/+ mice. Journal of environmental science and health Part A, Toxic/hazardous substances & environmental engineering 43: 1402–1409. Available: http://www.ncbi.nlm.nih.gov/pubmed/18780217. Accessed 19 October 2012.

25. Blossom SJ, Doss JC, Hennings LJ, Jernigan S, Melnyk S, et al. (2008) Developmental exposure to trichloroethylene promotes CD4+ T cell differentiation and hyperactivity in association with oxidative stress and neurobehavioral deficits in MRL+/+ mice. Toxicology and applied pharmacology 231: 344–353. Available: http://www.ncbi.nlm.nih.gov/pubmed/18579175. Accessed 19 October 2012.

26. Gillis B, Gavin IM, Arbieva Z, King ST, Jayaraman S, et al. (2007) Identification of human cell responses to benzene and benzene metabolites. Genomics 90: 324–333. Available: http://www.ncbi.nlm.nih.gov/pubmed/17572062. Accessed 19 October 2012.

27. Sheikh N, Dudas J, Ramadori G (2007) Changes of gene expression of iron regulatory proteins during turpentine oil-induced acute-phase response in the rat. Laboratory investigation; a journal of technical methods and pathology 87: 713–725. Available: http://www.ncbi.nlm.nih.gov/pubmed/17417667. Accessed 18 October 2012.

28. Kirkeleit J, Ulvestad E, Riise T, Bråtveit M, Moen BE (2006) Acute suppression of serum IgM and IgA in tank workers exposed to benzene. Scandinavian journal of immunology 64: 690–698. Available: http://www.ncbi.nlm.nih.gov/pubmed/17083627. Accessed 19 October 2012.

29. Blossom SJ, Doss JC, Gilbert KM (2007) Chronic exposure to a trichloroethylene metabolite in autoimmune-prone MRL+/+ mice promotes immune modulation and alopecia. Toxicological sciences : an official journal of the Society of Toxicology 95: 401–411. Available: http://www.ncbi.nlm.nih.gov/pubmed/17077186. Accessed 19 October 2012.

30. Martínez-Velázquez M, Maldonado V, Ortega A, Meléndez-Zajgla J, Albores A (2006) Benzene metabolites induce apoptosis in lymphocytes. Experimental and toxicologic pathology : official journal of the Gesellschaft für Toxikologische Pathologie 58: 65–70. Available: http://www.ncbi.nlm.nih.gov/pubmed/16713212. Accessed 19 October 2012.

31. Lee E, Im H, Oh E, Jung W-W, Kang H-S, et al. (n.d.) DNA damage in T and B lymphocytes, bone marrow, spleens, and livers of rats exposed to benzene. Inhalation toxicology 17: 401–408. Available: http://www.ncbi.nlm.nih.gov/pubmed/16020036. Accessed 19 October 2012.

32. Heijne WHM, Jonker D, Stierum RH, van Ommen B, Groten JP (2005) Toxicogenomic analysis of gene expression changes in rat liver after a 28-day oral benzene exposure. Mutation research 575: 85–101. Available: http://www.ncbi.nlm.nih.gov/pubmed/15878777. Accessed 19 October 2012.

33. Akbaş E, Derici E, Söylemez F, Kanik A, Polat F (2004) An investigation of effects of toluene and cigarette smoking on some blood parameters and lymphocyte life span. Cell biology and toxicology 20: 33–40. Available: http://www.ncbi.nlm.nih.gov/pubmed/15119846. Accessed 19 October 2012.

34. Gilbert KM, Whitlow AB, Pumford NR (2004) Environmental contaminant and disinfection by-product trichloroacetaldehyde stimulates T cells in vitro. International immunopharmacology 4: 25–36. Available: http://www.ncbi.nlm.nih.gov/pubmed/14975357. Accessed 19 October 2012.

35. Cho J-A, Oh E, Lee E, Sul D (2003) Effects of hair dyeing on DNA damage in human lymphocytes. Journal of occupational health 45: 376–381. Available: http://www.ncbi.nlm.nih.gov/pubmed/14676417. Accessed 19 October 2012.

36. Turteltaub KW, Mani C (2003) Benzene metabolism in rodents at doses relevant to human exposure from urban air. Research report (Health Effects Institute): 1–26; discussion 27–35. Available: http://www.ncbi.nlm.nih.gov/pubmed/12675491. Accessed 19 October 2012.

37. Jiménez-Alonso J, Sabio JM, Pérez-Alvarez F, Reche I, Hidalgo C, et al. (2002) Hair dye treatment use and clinical course in patients with systemic lupus erythematosus and cutaneous lupus. Lupus 11: 430–434. Available: http://www.ncbi.nlm.nih.gov/pubmed/12195784. Accessed 19 October 2012.

38. Biró A, Pállinger E, Major J, Jakab MG, Klupp T, et al. (2002) Lymphocyte phenotype analysis and chromosome aberration frequency of workers occupationally exposed to styrene, benzene, polycyclic aromatic hydrocarbons or mixed solvents. Immunology letters 81: 133–140. Available: http://www.ncbi.nlm.nih.gov/pubmed/11852118. Accessed 19 October 2012.

39. Tanigawa T, Araki S, Nakata A, Yokoyama K, Sakai T, et al. (n.d.) Decreases of natural killer cells and T-lymphocyte subpopulations and increases of B lymphocytes following a 5-day occupational exposure to mixed organic solvents. Archives of environmental health 56: 443–448. Available: http://www.ncbi.nlm.nih.gov/pubmed/11777026. Accessed 19 October 2012.

40. Green SM, Khan MF, Kaphalia BS, Ansari GA (2001) Immunohistochemical localization of trichloroacylated protein adducts in tetrachloroethene-treated mice. Journal of toxicology and environmental health Part A 63: 145–157. Available: http://www.ncbi.nlm.nih.gov/pubmed/11393800. Accessed 19 October 2012.

41. Kawasaki S, Takizawa H, Takami K, Desaki M, Okazaki H, et al. (2001) Benzene-extracted components are important for the major activity of diesel exhaust particles: effect on interleukin-8 gene expression in human bronchial epithelial cells. American journal of respiratory cell and molecular biology 24: 419–426. Available: http://www.ncbi.nlm.nih.gov/pubmed/11306435. Accessed 19 October 2012.

42. Khan MF, Wu X, Ansari GA (2001) Anti-malondialdehyde antibodies in MRL+/+ mice treated with trichloroethene and dichloroacetyl chloride: possible role of lipid peroxidation in autoimmunity. Toxicology and applied pharmacology 170: 88–92. Available: http://www.ncbi.nlm.nih.gov/pubmed/11162772. Accessed 19 October 2012.

43. Kalf GF (2000) Utility of a mouse model for studying the effects of benzene on the myeloid lineage: effects of hydroquinone on a model myeloid system. Journal of toxicology and environmental health Part A 61: 399–411. Available: http://www.ncbi.nlm.nih.gov/pubmed/11086946. Accessed 19 October 2012.

44. Christner PJ, Artlett CM, Conway RF, Jiménez SA (2000) Increased numbers of microchimeric cells of fetal origin are associated with dermal fibrosis in mice following injection of vinyl chloride. Arthritis and rheumatism 43: 2598–2605. Available: http://www.ncbi.nlm.nih.gov/pubmed/11083286. Accessed 19 October 2012.

45. Khalil Z, Georgiou GM, Ogedegbe H, Cone RE, Simpson F, et al. (n.d.) Immunological and in-vivo neurological studies on a benzoic acid-specific T cell-derived antigen-binding molecule from the serum of a toluene-sensitive patient. Archives of environmental health 55: 304–318. Available: http://www.ncbi.nlm.nih.gov/pubmed/11063405. Accessed 19 October 2012.

46. Bogadi-Sare A, Zavalic M, Trosić I, Turk R, Kontosić I, et al. (2000) Study of some immunological parameters in workers occupationally exposed to benzene. International archives of occupational and environmental health 73: 397–400. Available: http://www.ncbi.nlm.nih.gov/pubmed/11007343. Accessed 19 October 2012.

47. Griffin JM, Gilbert KM, Pumford NR (2000) Inhibition of CYP2E1 reverses CD4+ T-cell alterations in trichloroethylene-treated MRL+/+ mice. Toxicological sciences : an official journal of the Society of Toxicology 54: 384–389. Available: http://www.ncbi.nlm.nih.gov/pubmed/10774820. Accessed 19 October 2012.

48. Toraason M, Clark J, Dankovic D, Mathias P, Skaggs S, et al. (1999) Oxidative stress and DNA damage in Fischer rats following acute exposure to trichloroethylene or perchloroethylene. Toxicology 138: 43–53. Available: http://www.ncbi.nlm.nih.gov/pubmed/10566590. Accessed 19 October 2012.

49. Yücesoy B, Yücel A, Erdem O, Burgaz S, Imir T, et al. (1999) Effects of occupational chronic co-exposure to n-hexane, toluen, and methyl ethyl ketone on NK cell activity and some immunoregulatory cytokine levels in shoe workers. Human & experimental toxicology 18: 541–546. Available: http://www.ncbi.nlm.nih.gov/pubmed/10523867. Accessed 19 October 2012.

50. Ewens S, Wulferink M, Goebel C, Gleichmann E (n.d.) T cell-dependent immune reactions to reactive benzene metabolites in mice. Archives of toxicology 73: 159–167. Available: http://www.ncbi.nlm.nih.gov/pubmed/10401682. Accessed 19 October 2012.

51. Tuo J, Loft S, Poulsen HE (1999) Enhanced benzene-induced DNA damage in PMA-stimulated cells in vitro and in LPS-treated animals. Free radical biology & medicine 26: 801–808. Available: http://www.ncbi.nlm.nih.gov/pubmed/10232822. Accessed 19 October 2012.

52. Wu T, Yuan Y, Wu Y, He H, Zhang G, et al. (1998) Presence of antibodies to heat stress proteins in workers exposed to benzene and in patients with benzene poisoning. Cell stress & chaperones 3: 161–167. Available: http://www.pubmedcentral.nih.gov/articlerender.fcgi?artid=312960&tool=pmcentrez&rendertype=abstract. Accessed 19 October 2012.

53. Tuo J, Wolff SP, Loft S, Poulsen HE (1998) Formation of nitrated and hydroxylated aromatic compounds from benzene and peroxynitrite, a possible mechanism of benzene genotoxicity. Free radical research 28: 369–375. Available: http://www.ncbi.nlm.nih.gov/pubmed/9684981. Accessed 19 October 2012.

54. Pyatt DW, Stillman WS, Irons RD (1998) Hydroquinone, a reactive metabolite of benzene, inhibits NF-kappa B in primary human CD4+ T lymphocytes. Toxicology and applied pharmacology 149: 178–184. Available: http://www.ncbi.nlm.nih.gov/pubmed/9571986. Accessed 19 October 2012.

55. Vodela JK, Renden JA, Lenz SD, McElhenney WH, Kemppainen BW (1997) Drinking water contaminants (arsenic, cadmium, lead, benzene, and trichloroethylene). 1. Interaction of contaminants with nutritional status on general performance and immune function in broiler chickens. Poultry science 76: 1474–1492. Available: http://www.ncbi.nlm.nih.gov/pubmed/9355140. Accessed 19 October 2012.

56. Farris GM, Robinson SN, Wong BA, Wong VA, Hahn WP, et al. (1997) Effects of benzene on splenic, thymic, and femoral lymphocytes in mice. Toxicology 118: 137–148. Available: http://www.ncbi.nlm.nih.gov/pubmed/9129168. Accessed 19 October 2012.

57. Farris GM, Robinson SN, Gaido KW, Wong BA, Wong VA, et al. (1996) Effects of low concentrations of benzene on mouse hematopoietic cells in vivo: a preliminary report. Environmental health perspectives 104 Suppl: 1275–1276. Available: http://www.pubmedcentral.nih.gov/articlerender.fcgi?artid=1469735&tool=pmcentrez&rendertype=abstract. Accessed 19 October 2012.

58. Henschler R, Glatt HR, Heyworth CM (1996) Hydroquinone stimulates granulocyte-macrophage progenitor cells in vitro and in vivo. Environmental health perspectives 104 Suppl: 1271–1274. Available: http://www.pubmedcentral.nih.gov/articlerender.fcgi?artid=1469771&tool=pmcentrez&rendertype=abstract. Accessed 19 October 2012.

59. Hazel BA, O’Connor A, Niculescu R, Kalf GF (1996) Induction of granulocytic differentiation in a mouse model by benzene and hydroquinone. Environmental health perspectives 104 Suppl: 1257–1264. Available: http://www.pubmedcentral.nih.gov/articlerender.fcgi?artid=1469738&tool=pmcentrez&rendertype=abstract. Accessed 19 October 2012.

60. Kalf GF, Renz JF, Niculescu R (1996) p-Benzoquinone, a reactive metabolite of benzene, prevents the processing of pre-interleukins-1 alpha and -1 beta to active cytokines by inhibition of the processing enzymes, calpain, and interleukin-1 beta converting enzyme. Environmental health perspectives 104 Suppl: 1251–1256. Available: http://www.pubmedcentral.nih.gov/articlerender.fcgi?artid=1469767&tool=pmcentrez&rendertype=abstract. Accessed 19 October 2012.

61. Niculescu R, Renz JF, Kalf GF (1996) Benzene-induced bone marrow cell depression caused by inhibition of the conversion of pre-interleukins-1alpha and -1beta to active cytokines by hydroquinone, a biological reactive metabolite of benzene. Advances in experimental medicine and biology 387: 329–337. Available: http://www.ncbi.nlm.nih.gov/pubmed/8794226. Accessed 19 October 2012.

62. Niculescu R, Bradford HN, Colman RW, Kalf GF (1995) Inhibition of the conversion of pre-interleukins-1 alpha and 1 beta to mature cytokines by p-benzoquinone, a metabolite of benzene. Chemico-biological interactions 98: 211–222. Available: http://www.ncbi.nlm.nih.gov/pubmed/8548860. Accessed 19 October 2012.

63. Carbonnelle P, Lison D, Leroy JY, Lauwerys R (1995) Effect of the benzene metabolite, hydroquinone, on interleukin-1 secretion by human monocytes in vitro. Toxicology and applied pharmacology 132: 220–226. Available: http://www.ncbi.nlm.nih.gov/pubmed/7540334. Accessed 19 October 2012.

64. d’ Azevedo PA, Tannhauser M, Tannhauser SL, Barros HM (1996) Hematological alterations in rats from xylene and benzene. Veterinary and human toxicology 38: 340–344. Available: http://www.ncbi.nlm.nih.gov/pubmed/8888536. Accessed 20 October 2012.

65. Laskin JD, Rao NR, Punjabi CJ, Laskin DL, Synder R (1995) Distinct actions of benzene and its metabolites on nitric oxide production by bone marrow leukocytes. Journal of leukocyte biology 57: 422–426. Available: http://www.ncbi.nlm.nih.gov/pubmed/7884313. Accessed 19 October 2012.

66. Fan XH (1992) Effect of exposure to benzene on natural killer (NK) cell activity and interleukin-2 (IL-2) production of C57BL/6 mice. Nihon Ika Daigaku zasshi 59: 393–399. Available: http://www.ncbi.nlm.nih.gov/pubmed/1430111. Accessed 19 October 2012.

67. Schlichting LM, Wright PF, Stacey NH (n.d.) Effects of tetrachloroethylene on hepatic and splenic lymphocytotoxic activities in rodents. Toxicology and industrial health 8: 255–266. Available: http://www.ncbi.nlm.nih.gov/pubmed/1455436. Accessed 19 October 2012.

68. MacEachern L, Laskin DL (1992) Increased production of tumor necrosis factor-alpha by bone marrow leukocytes following benzene treatment of mice. Toxicology and applied pharmacology 113: 260–266. Available: http://www.ncbi.nlm.nih.gov/pubmed/1561635. Accessed 19 October 2012.

69. Seidel HJ, Weber L, Barthel E (1992) Hematological toxicity of tetrachloroethylene in mice. Archives of toxicology 66: 228–230. Available: http://www.ncbi.nlm.nih.gov/pubmed/1497490. Accessed 19 October 2012.
